# Uncovering New Drug Properties in Target-Based Drug–Drug Similarity Networks

**DOI:** 10.3390/pharmaceutics12090879

**Published:** 2020-09-16

**Authors:** Lucreţia Udrescu, Paul Bogdan, Aimée Chiş, Ioan Ovidiu Sîrbu, Alexandru Topîrceanu, Renata-Maria Văruţ, Mihai Udrescu

**Affiliations:** 1 Department I-Drug Analysis, “Victor Babeş” University of Medicine and Pharmacy Timişoara, 2 Eftimie Murgu Sq., 300041 Timişoara, Romania; udrescu.lucretia@umft.ro; 2 Ming Hsieh Department of Electrical Engineering, University of Southern California, 3740 McClintock Ave., Los Angeles, CA 90089-2563, USA; pbogdan@usc.edu; 3 Department of Biochemistry and Pharmacology-Biochemistry, “Victor Babeş” University of Medicine and Pharmacy Timişoara, 2 Eftimie Murgu Sq., 300041 Timişoara, Romania; chis.aimee@umft.ro (A.C.); ovidiu.sirbu@umft.ro (I.O.S.); 4 Timişoara Institute of Complex Systems, 18 Vasile Lucaciu Str., 300044 Timişoara, Romania; 5 Department of Computer and Information Technology, University Politehnica of Timişoara, 2 Vasile Pârvan Blvd., 300223 Timişoara, Romania; alexandru.topirceanu@cs.upt.ro; 6 Pharmacy Department I, University of Medicine and Pharmacy, 2-4 Petru Rares Str., 200349 Craiova, Romania; rennata_maria@yahoo.com

**Keywords:** drug repurposing, drug–target interactions, drug–drug similarity network, network clustering, network centrality, molecular docking

## Abstract

Despite recent advances in bioinformatics, systems biology, and machine learning, the accurate prediction of drug properties remains an open problem. Indeed, because the biological environment is a complex system, the traditional approach—based on knowledge about the chemical structures—can not fully explain the nature of interactions between drugs and biological targets. Consequently, in this paper, we propose an unsupervised machine learning approach that uses the information we know about drug–target interactions to infer drug properties. To this end, we define drug similarity based on drug–target interactions and build a weighted Drug–Drug Similarity Network according to the drug–drug similarity relationships. Using an energy-model network layout, we generate drug communities associated with specific, dominant drug properties. DrugBank confirms the properties of 59.52% of the drugs in these communities, and 26.98% are existing drug repositioning hints we reconstruct with our DDSN approach. The remaining 13.49% of the drugs seem not to match the dominant pharmacologic property; thus, we consider them potential drug repurposing hints. The resources required to test all these repurposing hints are considerable. Therefore we introduce a mechanism of prioritization based on the betweenness/degree node centrality. Using betweenness/degree as an indicator of drug repurposing potential, we select Azelaic acid and Meprobamate as a possible antineoplastic and antifungal, respectively. Finally, we use a test procedure based on molecular docking to analyze Azelaic acid and Meprobamate’s repurposing.

## 1. Introduction

**Figure 1:**
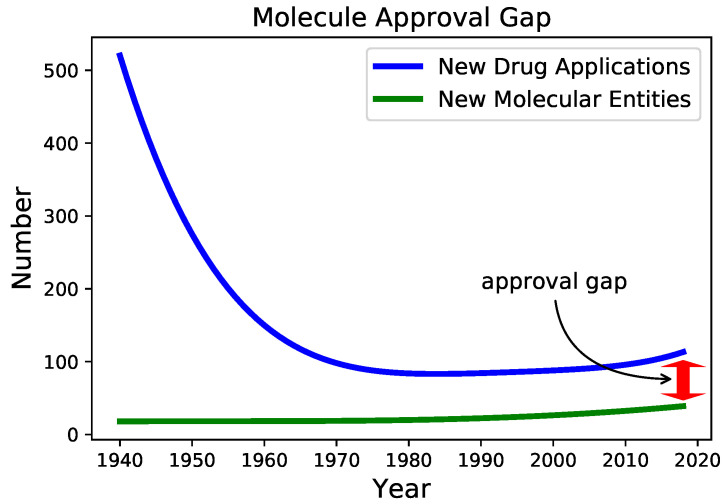
The evolution of New Drug Applications (NDAs) and New Molecular Entities (NME) during 1940–2017. We used the FDA’s annual reports data [6] and removed local oscillations by plotting a polynomial data fitting.

Conventional drug design has become expensive and cumbersome, as it requires large amounts of resources and faces serious challenges [1,2]. Consequently, although the number of new FDA drug applications (NDAs) has significantly increased during the last decade—due to a spectacular accumulation of multi-omics data and the appearance of increasingly complex bioinformatics tools—the number of approved drugs has only marginally grown (see Figure 1) [3,4], calling for more robust alternative strategies [5].

One of the most effective alternative strategies is drug repositioning (or drug repurposing) [7,8], namely finding new pharmaceutical functions for already used drugs. The extensive medical and pharmaceutical experience reveals a surprising propensity towards multiple indications for many drugs [9], and the examples of successful drug repositioning are steadily accumulating. Out of the 90 newly approved drugs in 2016 (a 10% decrease from 2015), 25% are repositionings in formulations, combinations, and indications [4]. Furthermore, drug repositioning reduces the incurred research and development (R&D) time and costs and medication risks [9,10].

The recent developments confirm computational methods as powerful tools for drug repositioning:

- The trivialization/spread of omics analytical approaches have generated significant volumes of useful multi-omics data (genomics, proteomics, metabolomics, and others) [11,12].
- The ubiquity of digitalization in everyday life, including social media, has tremendously expanded the amplitude of the process of gathering data on drug–drug interactions and drug side-effects [13,14].
- The recent developments in physics, computer science, and computer engineering have created efficient methods and technologies for data exploration and mining, such as complex network analysis, machine learning, or deep learning [12,15,16,17,18,19].

Complex network analysis has proven to be a useful tool for predicting unaccounted drug–target interactions. Indeed, several state-of-the-art network-based computational drug repurposing approaches use data on confirmed drug–target interactions to predict new such interactions, thus leading to new repositioning hints [20,21]. Some approaches build drug–drug similarity networks, where the similarity is defined based on transcriptional responses [22,23]. These repositioning approaches analyze the network parameters and the node centrality distributions in either drug–drug or drug–target networks, using statistical analysis [11,12,24,25] and machine learning (i.e., graph convolutional networks) [26,27,28,29] to link certain drugs to new pharmacological properties. However, conventional statistics can be misleading when used to predict extreme centrality values, such as degree and betweenness (which particularly indicate nodes/drugs with a high potential for repositioning) [30]. Nonetheless, other previous network-related approaches introduce useful repositioning pipelines [31,32], but they are mostly based on multi-partite and multilayered unweighted networks, challenging to process and interpret.

To overcome these challenges, we developed a novel, network-based, computational approach to drug repositioning. To this end, we build a weighted drug–drug network, i.e., a complex network where the nodes are drugs, and the weighted links represent relationships between drugs, using information from the accurate DrugBank [33]. In our drug–drug similarity network (DDSN), a link is placed between two drugs if their interaction with at least one target is of the same type (either agonistic or antagonistic). The link weight represents the number of biological targets that interact in the same way with the two drugs.

Our methodology for analyzing the drug–drug similarity network (DDSN) consists of the following steps:

1. Generate (using the Force Atlas 2 layout and modularity classes) [34,35] both topological clusters and network communities.
2. Relate each cluster and each community to a pharmacological property or pharmacological action (i.e., label communities and clusters according to the dominant property or pharmacological action), using expert analysis.
3. Identify and select (by betweenness divided by degree, b/d) within each topological cluster/modularity class community, the top drugs not compliant with the cluster/community label. Network analysis uses centralities to rank nodes (i.e., drugs); we opt for the b/d centrality to find this centrality’s distribution more stable in the DDSN.
4. Validate the hinted repositionings by searching the new versions of DrugBank, the electronic records containing the relevant scientific literature (for merely reconstructed repositionings), and by analyzing molecular docking parameters [36] for previously unaccounted repositionings.

This way, we assessed our method’s ability to uncover new repositionings by confronting the results with the latest (version 5.1.4) Drug Bank and data compiled from interrogating scientific literature databases.

## 2. Materials and Methods

### 2.1. Building the DDSN

**Figure 2:**
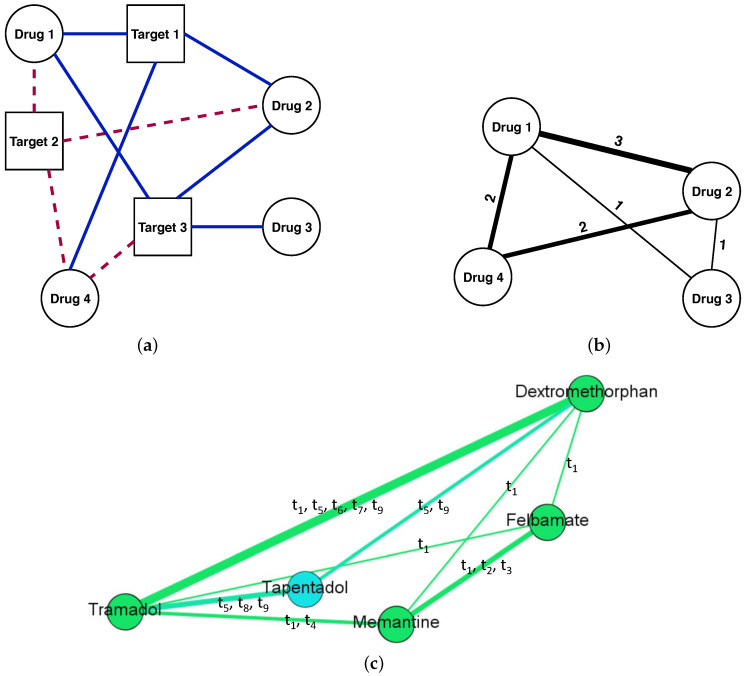
An illustrative example of using drug–target interaction information to build a weighted drug–drug similarity network. In panel (**a**), we consider the drug–target interactions between four drugs (i.e., round nodes labeled 1 to 4) and three targets (i.e., square nodes labeled 1 to 3). The dashed red links represent agonist drug–target interactions, whereas the solid blue links represent antagonist drug–target interactions. In panel (**b**), we show the DDSN corresponding to the interactions in (**a**). For instance, a link of weight 3 connects the nodes 1 and 2 because Drug 1 and Drug 2 interact in the same way for the three targets, i.e., agonist on Target 2 and antagonist on Targets 1 and 3. Furthermore, a link with weight 2 connects Drug 2 and Drug 4 because they both interact agonistically on Target 2 and antagonistically on Target 1, but they do not interact in the same way with Target 3. In panel (**c**), we show a DDSN sub-network example, according to drug–target interactions from DrugBank 4.2, containing drugs Dextromethorphan, Felbamate, Tapentadol, Tramadol, and Memantine. We shape the link thickness according to the weight and specify the list of common targets for each link. The weight equals the number of targets in the list, where $t_{1}$ = Glutamate receptor ionotropic NMDA 3A, $t_{2}$ = Glutamate receptor ionotropic NMDA 2A, $t_{3}$ = Glutamate receptor ionotropic NMDA 2B, $t_{4}$ = Alpha-7 nicotinic cholinergic receptor subunit, $t_{5}$ = Mu-type opioid receptor, $t_{6}$ = Kappa-type opioid receptor, $t_{7}$ = Delta-type opioid receptor, $t_{8}$ = Sodium-dependent noradrenaline transporter, and $t_{9}$ = Sodium-dependent serotonin transporter.

We built our DDSN as a weighted graph $G=\left(V,E\right)$, where *V* is the vertex (or node) set, and *E* is the edge (or link) set; the vertices (nodes) represent drugs and the edges (links) represent drug–drug similarity relationships based on drug–target interactions. *G* has |V| vertices $v_{i}\in V$ and |E| edges $e_{j,k}\in E$, with $i,j,k\in \left\{1,2,\cdots |V|\right\}$ and j≠k. Each edge $e_{j,k}$ is characterized by a weight $w(e_{j,k})\neq 0$ (in an unweighted network, $w(e_{j,k})=1$, $\forall e_{j,k}\in E$). In our weighted DDSN, the weight represents the degree of target action similarity between drugs $v_{j}$ and $v_{k}$, and it is equal with the number of common biological targets for $v_{j}$ and $v_{k}$. Consequently, $w\left(e_{j,k}\right)\in N^{*}$, $\forall e_{j,k}\in E$. If $e_{j,k}=0$, then there is no target similarity between $v_{j}$ and $v_{k}$, therefore no edge between these nodes. A common biological target is a target $t_{k}\in T$ (*T* is the set of targets) on which drugs $v_{j}$ and $v_{k}$ act in the same way, either both agonistically or both antagonistically. Figure 2 illustrates the building of the DDSN with information on drug–target interactions.

For the DDSN graph *G*, we use the drug–target interaction information from Drug Bank 4.2 [33]. We base our analysis on the largest connected component of the DDSN, consisting of |V|=1008 drugs/nodes and |E|=17963 links resulted from the analysis of the drug–target interactions with |T|=516 targets. We opted for the older Drug Bank version 4.2 [33], to be able to use the latest Drug Bank 5.1.4 [37] for testing the accuracy of our drug property prediction.

### 2.2. Network Analysis

This paper uses complex network analysis tools to uncover new drug properties from the drug–target data. We employ network clustering (i.e., network community detection) to associate drugs with previously unaccounted drug properties and network centralities to prioritize the uncovered drug repurposing hints.

#### 2.2.1. Network Clustering

The network clustering classifies each node $v_{i}\in V$ in one of the disjoint sets of nodes (cluster $C_{i}\subset V$, with $i=\bar{1..m}$, $C_{1}⋃C_{2}\cdots ⋃C_{m}=V$). In [35], the authors use modularity to define the node membership to one of the clusters. To this end, the modularity of a clustering $C_{m}=\left\{C_{1},C_{2},\cdots C_{m}\right\}$ is

(1) $$M_{m}=\sum_{C_{i}\in C_{m}}\left(\frac{|E_{C_{i}}|}{\left|E\right|}-\frac{\frac{1}{2}{d_{C_{i}}}^{2}}{\frac{1}{2}d^{2}}\right)$$

where |E| is the total number of edges in *G*, $|E_{C_{i}}|$ is the total number of edges between nodes in cluster $C_{i}$, *d* is the total degree of nodes in *G*, and $d_{C_{i}}$ is the total degree of nodes in cluster $C_{i}$. Thus, $\frac{|E_{C_{i}}|}{\left|E\right|}$ represents the edge density of cluster $C_{i}$ relative to the entire network *G* density, whereas $\frac{\frac{1}{2}{d_{C_{i}}}^{2}}{\frac{1}{2}d^{2}}$ is the $C_{i}$’s expected relative density.

We perform clustering using the software package Gephi [38], by maximizing the modularity from Equation (1) with the method introduced and analyzed in references [39,40]. The approach is to divide a graph into two communities, such that we get maximum modularity. The binary method can then be applied recursively on each resulted community, thus dividing them further; the entire process comes to an end when we cannot further increase the overall modularity. To describe the division algorithm, we write the graph modularity as

(2) $$\begin{array}{c} M=\frac{1}{4k}\sum_{ij}\left(A_{ij}-\frac{d_{i}d_{j}}{2k}\right)\left(s_{i}s_{j}+1\right). \end{array}$$

In Equation (2), $A_{ij}$ is the graph’s adjacency matrix, $d_{i}$ and $d_{j}$ are respectively the degrees of vertices/nodes $v_{i}$ and $v_{j}$, and *k* is the total number of edges in the network ($k=\left|E\right|=\frac{1}{2}\sum_{i}\;d_{i}$ for an unweighted network). Furthermore, $s_{i}=1$ if $v_{i}$ is classified in community 1 and $s_{i}=-1$ if $v_{i}$ is classified in community 2 [41]. Therefore, we have

(3) $$\begin{array}{c} \frac{1}{2}\left(s_{i}s_{j}+1\right)\;=\left\{\begin{array}{cc} 1 & \mathrm{if}\;v_{i}\;\mathrm{and}\;v_{j}\;\mathrm{are}\;\mathrm{in}\;\mathrm{the}\;\mathrm{same}\;\mathrm{community}\\ 0 & \mathrm{otherwise} \end{array}\right.. \end{array}$$

For a detailed description of the clustering algorithm, please refer to the pdf Supplementary Information, Section S1.

Because our network is weighted, each edge has a weight $w\left(e_{i,j}\right)=w_{i,j}\in R^{*}$, and we rewrite Equation (1) as

(4) $$M_{m}=\sum_{C_{i}\in C_{m}}\left(\frac{w_{E_{C_{i}}}}{w_{E}}-\frac{\frac{1}{2}w_{C_{i}}^{2}}{\frac{1}{2}w_{V}^{2}}\right).$$

In Equation (4), $w_{E}$ is the total edge weight of edges *E* in *G*, $w_{E_{C_{i}}}$ is the total edge weight of edges in cluster $C_{i}$, $w_{V}$ is the total edge weight of all vertices *V* in *G*, and $w_{C_{i}}$ is the total edge weight of vertices in cluster $C_{i}$.

A network layout algorithm places each vertex $v_{i}$ in a 2D space $R\times R=R^{2}$. Therefore, each node $v_{i}\in V$ has its 2D coordinates $\gamma_{i}=\left(x_{i},y_{i}\right)\in R^{2}$, and each edge $e_{i,j}\in E$ has a Euclidian distance $\delta_{i,j}=\left|\gamma_{i}-\gamma_{j}\right|$. In an energy-model, force-directed layout, we have a force of attraction between any two adjacent nodes $v_{i}$ and $v_{j}$, and a repulsion force between any two non-adjacent nodes. The expression of these forces is ${|\gamma_{i}-\gamma_{j}|}^{f}\vec{\gamma_{i}\gamma_{j}}$, where f=a for attraction and f=r for repulsion. The attraction force between adjacent nodes ($v_{i}$ and $v_{j}$ such that $\exists e_{i,j}\in E$) decreases, whereas the repulsion force between non-adjacent nodes ($v_{i},v_{j}$ such that $\exists !e_{i,j}\in E$) increases with the Euclidian distance. Therefore, we must have a≥0 and r≤0.

In this paper, we use the energy-model force-directed layout Force Atlas 2 [34] to assign node positions in the 2D (i.e., $R^{2}$) space, based on interactions between attraction and repulsion forces, such that we attain minimal energy in the network layout,

(5) $$E=\min\left\{\sum_{\left(v_{i},v_{j}\right),i\neq j}\left(\frac{{|\gamma_{i}-\gamma_{j}|}^{a}}{a+1}-\frac{{|\gamma_{i}-\gamma_{j}|}^{r}}{r+1}\right)\right\}.$$

The energy-based layouts generate topological communities because specific regions in the network have larger than average link densities. Noack [41] demonstrated that the energy-based topological communities are equivalent to the network clusters based on modularity classes [35], when a>−1 and r>−1. Furthermore, given that our DDSN is a weighted network, we rewrite Equation (5) accordingly, to maintain equivalency with Equation (4),

(6) $$E=\min\left\{\sum_{\left(v_{i},v_{j}\right),i\neq j}\left(w_{i,j}\frac{{\left|\gamma_{i}-\gamma_{j}\right|}^{a}}{a+1}-w_{i}w_{j}\frac{{\left|\gamma_{i}-\gamma_{j}\right|}^{r}}{r+1}\right)\right\}$$

where $w_{i}$ and $w_{j}$ represent the total weight of edges incident to nodes $v_{i}$ and $v_{j}$ (i.e., the weighted degree of vertices $v_{i}$ and $v_{j}$), respectively, while $w_{i,j}$ is the weight of edge $e_{i,j}$.

#### 2.2.2. Network Centralities

Node centralities are complex network parameters that characterize the vertex/node’s importance in a graph [42]. In our analysis, we considered the weighted degree, degree, betweenness, and betweenness/degree node centralities, to find that betweenness/degree is appropriate for the prioritizing of drug repositioning hint tests. Reference [43] shows that the betweenness/degree centrality is a crucial driver of complex network dynamics.

The weighted degree of a node $v_{i}$ is the sum of the weights characterizing the links/edges incident to $v_{i}$,

(7) $$d\left(v_{i}\right)=\sum_{j\in \left\{x|e_{i,x}\in E,v_{x},v_{i}\in V\right\}}w\left(e_{i,j}\right).$$

We compute the degree of a node $v_{i}$ with Equation (7), assuming that $w\left(e_{i,j}\right)=1$, $\forall e_{i,j}\in E$.

To compute the node betweenness, we must find the shortest paths between all node pairs $\left(v_{j},v_{k}\right)$ in graph *G*, namely $\sigma_{j,k}$. As such, the betweenness of node $v_{i}$ is the number of minimal paths in graph *G* that cross node $v_{i}$, divided by the total number of minimal paths in *G*,

(8) $$b\left(v_{i}\right)=\sum_{\left(j,k\right)\in \left\{\left(x,y\right)|v_{x}\neq v_{y}\neq v_{i};v_{x},v_{y},v_{i}\in V\right\}}\frac{\sigma_{j,k}\left(v_{i}\right)}{\sigma_{G}},$$

where the total number of shortest paths in *G* is the combinations of 2 vertices from *V*,

(9) σG=|V|2.

The betweenness/degree of node $v_{i}$ is the ratio

(10) $$b/d\left(v_{i}\right)=\frac{b\left(v_{i}\right)}{d\left(v_{i}\right)},$$

where Equation (7) computes $d\left(v_{i}\right)$ in the unweighted version (i.e., considering $w\left(e_{i,j}\right)=1$, $\forall e_{i,j}\in E$).

### 2.3. Molecular Docking for Repurposing Testing

The effectiveness of out network-based drug repurposing prediction method is emphasized by the fact that DrugBank 4.2 confirms the properties we predict for 59.52% of the drugs, and 26.98% are existing drug repositioning hints we reconstruct with our DDSN approach (confirmed by the later DrugBank 5.1.4 and recent scientific literature). The remaining 13.49% of the drugs seem not to match the predicted pharmacologic property; therefore, we consider them potential drug repurposing hints that need to be tested in silico, in vitro, and in vivo. Here, we propose a preliminary testing method based on molecular docking simulations.

#### 2.3.1. Testing Procedure

To verify the predicted properties of any repurposing hint, we perform molecular docking not only for the hint but also for the reference drugs (typical drugs having the predicted property) and some drugs with little probability of having the predicted property. To this end, we formalize the following testing procedure.

1. We define the drug sets to enter the docking process, consisting of drugs hinted as having the pharmacological property ϕ ($D_{h}^{\phi }$), well-documented drugs with property ϕ (reference drugs $D_{r}^{\phi }$), and drugs with little probability of having property ϕ ($D_{n}^{\phi }$). Our goal is to explore the similarity (in terms of relevant target activity) between the reference drugs $D_{r}^{\phi }$ and the tested drugs $D_{t}^{\phi }=D_{h}^{\phi }⋃D_{n}^{\phi }$.
  (a) $D_{h}^{\phi }$ consists of the drugs hinted as repurposed for property/properties ϕ.
  (b) $D_{r}^{\phi }$ consists of two subsets, reference drugs in the DDSN’s community $C_{x}$ ($D_{x}^{\phi }$) and reference drugs not in $C_{x}$ ($D_{\bar{x}}^{\phi }$), with $D_{r}^{\phi }=D_{x}^{\phi }⋃D_{\bar{x}}^{\phi }$.
  (c) $D_{n}^{\phi }$ contains typical drugs for other pharmacological properties, with little probability of having property ϕ.
2. We establish the target sets. Specifically, for pharmacological property ϕ, we take into consideration the targets from DrugBank that interact with the drugs in the hinted drug $d_{h}^{\phi }$ community $C_{x}$ having property ϕ ($T_{x}^{\phi }$), and the targets from DrugBank that interact with the drugs with property ϕ not included in DDSN’s $C_{x}$ ($T_{\bar{x}}^{\phi }$).
3. For the set of tested drugs $D_{t}^{\phi }$, we use molecular docking to check the interactions between all possible drug–target pairs, defined as the Cartesian product of sets $D_{t}^{\phi }$ and $T^{\phi }$ (with $T^{\phi }\;=\;T_{x}^{\phi }⋃T_{\bar{x}}^{\phi }$),
  (11) $$D_{t}^{\phi }\times T^{\phi }=\left\{\left(d_{i},t_{j}\right):d_{i}\in D_{t}^{\phi },t_{j}\in T^{\phi },\forall i,j\in N^{*},i\leq |D_{t}^{\phi }\left|,j\leq \right|T^{\phi }|\right\}.$$
4. For the set of reference drugs, we apply molecular docking on separately designed drug–target pairs for reference drugs in $C_{x}$ ($D_{x}^{\phi }$), and reference drugs not in $C_{x}$ ($D_{\bar{x}}^{\phi }$) respectively, such that any drug–target pair is well-documented in the literature,
  (12) $$\left\{\left(d_{i},t_{j}\right):d_{i}\in D_{x}^{\phi },t_{j}\in T_{x}^{\phi },\forall i,j\in N^{*},i\leq |D_{x}^{\phi }\left|,j\leq \right|T_{x}^{\phi }|,l\left(i,j\right)=1\right\}$$
  and
  (13) $$\left\{\left(d_{i},t_{j}\right):d_{i}\in D_{\bar{x}}^{\phi },t_{j}\in T_{\bar{x}}^{\phi },\forall i,j\in N^{*},i\leq |D_{\bar{x}}^{\phi }\left|,j\leq \right|T_{\bar{x}}^{\phi }|,l\left(i,j\right)=1\right\}.$$
  In Equations (12) and (13), Boolean function *l* is defined as
  (14) $$l\left(i,j\right)=\left\{\begin{array}{cc} 1 & \;\mathrm{if}\;\mathrm{the}\;\mathrm{interaction}\;\mathrm{between}\;\mathrm{drug}\;d_{i}\;\mathrm{and}\;\mathrm{target}\;t_{j}\;\mathrm{is}\;\mathrm{listed}\;\mathrm{in}\;\mathrm{DrugBank}\;\\ 0 & \;\mathrm{otherwise}\; \end{array}\right..$$

#### 2.3.2. Ligands and Targets Preparation

We generate all ligands’ three-dimensional coordinates using the Gaussian program suite with the DFT/B3LYP/6-311G optimization procedure.

We get the X-ray crystal structure of the targets as target.pdb files from the major protein databases Protein Data Bank [44] and optimize them with the ModRefiner software [45]. The targets and their corresponding codes are Lanosterol 14-alpha demethylase (4LXJ, resolution 1.9 Å), Intermediate conductance calcium-activated potassium channel protein 4 (6D42, resolution 1.75 Å), Lanosterol synthase (1W6K, resolution 2.1 Å), Squalene monooxygenase (6C6N, resolution 2.3 Å), Ergosterol (2AIB, resolution 1.1 Å), Sodium/potassium-transporting ATPase subunit alpha (2ZXE, resolution 2.4 Å), Tubulin (4U3J, resolution 2.81 Å), Progesterone receptor (1A28, resolution 1.8 Å), Androgen receptor (5JJM, resolution 2.15 Å), Estrogen receptor beta (3OLL, resolution 1.5 Å), Estrogen receptor alpha (1A52, resolution 2.8 Å), Steroid 17-alpha-hydroxylase/17,20 lyase (4NKV, resolution 2.646 Å), and Mineralocorticoid receptor (2OAX, resolution 2.29 Å). The preparation of targets also requires adding all polar hydrogens, removing the water, and computing the Gasteiger charge.

#### 2.3.3. Docking Protocol

We perform the molecular docking analysis using Autodock 4.2.6 with the molecular viewer and graphical support AutoDockTools [46].

In the docking protocol, for the protein targets, we create the grid box using Autogrid 4 with 120 Å × 120 Å × 120 Å in *x*, *y*, and *z* directions, and 1 Å spacing from the target molecule’s center. For steroidal target Ergosterol, the grid box is 30 Å × 30 Å × 30 Å in *x*, *y*, and *z* directions, with 0.375 Å spacing from the target molecule’s center.

For the docking process, we chose the Lamarckian genetic algorithm (Genetic Algorithm combined with a local search), with a population size of 150, a maximum of $2.5\times 10^{6}$ energy evaluations, a gene mutation rate of 0.02, and 50 runs. We adopted the default settings for the other docking parameters and performed all the calculations in vacuum conditions. We then exported all AutoDock results in the PyMOL (The PyMOL Molecular Graphics System, Version 2.0 Schrödinger, LLC, New York, NY, USA) and the Discovery Studio (Biovia) molecular visualization system (BIOVIA, Dassault Systèmes, BIOVIA Workbook, Release 2017; BIOVIA Pipeline Pilot, Release 2017, San Diego: Dassault Systèmes, 2019, San Diego, CA, USA).

We evaluate the performance of Autodock 4.2.6 by redocking and then expressing the results as root-mean-square deviation (RMSD) in Å. We perform all the calculations in duplicate and express the results as averages. The redocking involves the overlapping of the ligands for calculating the RMSD with the Discovery Studio software. We also run a comparative RMSD analysis between Autodock 4.2.6 and AutoDock Vina to assess the docking method’s repeatability and reproducibility.

## 3. Results

### 3.1. DDSN Analysis

**Figure 3:**
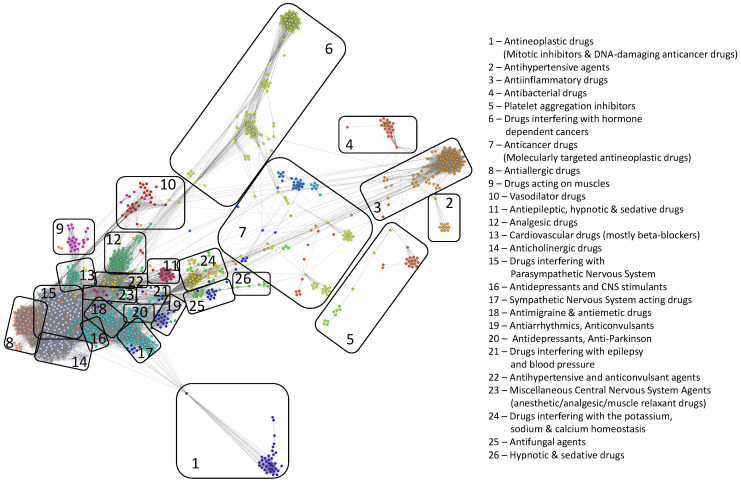
The drug–drug similarity network, where nodes represent drugs and links represent drug–drug similarity relationships based on drug–target interaction behavior. The layout is Force Atlas 2 [34], and the distinct node colors identify the modularity classes that define the drug clusters. We identify the 26 topological clusters with rounded rectangles and provide the functional descriptions for each of them.

Figure 3 illustrates the resulted DDSN, built according to our method, where the node colors identify the distinct modularity clusters.

To mine the DDSN topological complexity, we identified the drug clusters (or communities) using both the modularity [35] and the force-directed, energy-based layout Force Atlas 2 [34] algorithms. The two clustering techniques are compatible [41]; however, the energy-based force-directed layout clustering offers more information about the relationship between clusters and acts as an efficient classifier [47]. In the case of DDSN, the clusters correspond to drug communities $C_{x}$, $x\in N^{*}$, such that $V={⋃}_{i=1}^{m}C_{x}$.

Using the constructed DDSN from Drug Bank 4.2 and expert analysis, we label each cluster according to its dominant property (i.e., the property that better describes the majority of drugs in the cluster—see Supplementary file SupplementaryDDSN for detailed proof), which may represent a specific mechanism of pharmacologic action, a specifically targeted disease, or a targeted organ. We also confirm the clustering consistency across multiple DrugBank versions in pdf Supplementary Information, Section S2, Figure C1.

When using network clustering, if a drug does not comply with the community/cluster label, then this indicates a possible repurposing [48]. We labeled the clusters using the drug properties listed by DrugBank or reported in the literature, such that the dominant property or properties (i.e., properties found in more than 50% of the drugs in the community) give the name of the community, as indicated in Table 1 and Table 2.

According to Table 1 and Table 2 (column Literature [%]), our DDSN computational approach recovers/reconstructs a significant number of drug repurposings reported in the literature (see the Supplementary file SupplementaryDDSN for detailed confirmation literature lists, including some recent repurposing confirmations), namely 26.98% of the 1008 drugs in DDSN (the last line in Table 2, summarizing the confirmation results).

### 3.2. Illustrative Examples of Reconstructed Drug Repositionings

Here, we present a few illustrative examples of reconstructed drug repositionings, as confirmed by recent literature. We provide the entire list of drug repositionings we recovered with the DDSN method and the references that prove them as such in the Supplementary file SupplementaryDDSN.

#### 3.2.1. Reconstructed Repurposings as Antineoplastic Agents

**Figure 4:**
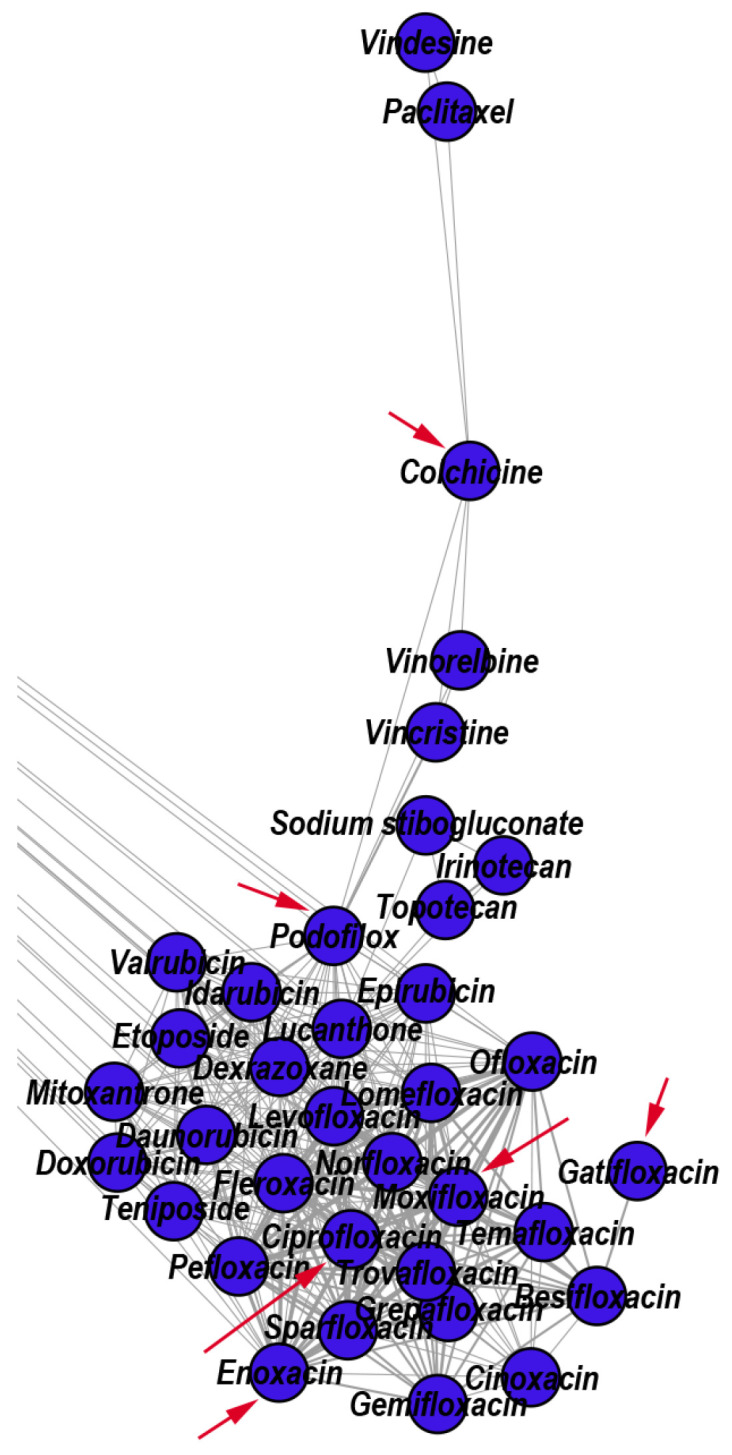
Zoomed DDSN detail of Community 1 ($C_{1}$, Antineoplastic drugs-–-Mitotic inhibitors & DNA-damaging anticancer drugs). The red arrows indicate the reconstructed drug repositionings: Colchicine (antigout drug), Podofilox (topical antiviral), and Enoxacin, Ciprofloxacin, Moxifloxacin, Gatifloxacin (fluoroquinolone antibiotics).

The topological community 1 (i.e., $C_{1}$) consists of antineoplastic drugs, mostly mitotic inhibitors (e.g., Etoposide, Teniposide, Vincristine, Vinorelbine) and DNA-damaging anticancer drugs (e.g., Doxorubicin, Valrubicin, Mitoxantrone). This community also contains fluoroquinolone antibiotics (targeting the alpha subunits of two types of bacterial topoisomerase II enzymes, namely DNA gyrase and DNA topoisomerase 4) and a few other drugs. However, DrugBank does not confirm some drugs’ anticancer effects within topological $C_{1}$, yet the literature confirms them as such. For example, Colchicine, which is currently used based on its anti-inflammatory effects as an antigout drug, exhibits anticancer effects [49]; Podofilox, a drug for topical treatment of external genital warts, is a potent cytotoxic agent in chronic lymphocytic leukemia (CLL) [50]; for some fluoroquinolone drugs, the literature reports anticancer effects (e.g., Enoxacin [51], Ciprofloxacin [52], Moxifloxacin [53], Gatifloxacin [54]). In Figure 4, we show a zoomed detail from our DDSN, by highlighting the presence of Colchicine, Podofilox, Enoxacin, Ciprofloxacin, Moxifloxacin, Gatifloxacin in $C_{1}$; such topological placement suggests their antineoplastic effect.

The topological community $C_{6}$ consists of anticancer drugs that target hormone-dependent organs (i.e., ovary, endometrium, vagina, cervix, and prostate). In this community, Progesterone has the highest value of betweenness/degree ratio, and the DrugBank database does not indicate its anticancer property. Although there are extensive epidemiological studies that link the long-term Progesterone use in oral contraceptives to breast cancer risk, this link is strengthened or weakened by various parameters, such as body weight, age, duration of use [55], parity, age at first birth, breastfeeding, and age at menarche [56]. However, J.C. Leo et al. determined the whole genomic effect of Progesterone in PR-transfected MDA-MB-231 cells and found that Progesterone suppressed the expression of genes involved in cell proliferation and metastasis, concluding that Progesterone can exert a strong anticancer effect in hormone-independent breast cancer following Progesterone receptor (PR) reactivation [57].

**Figure 5:**
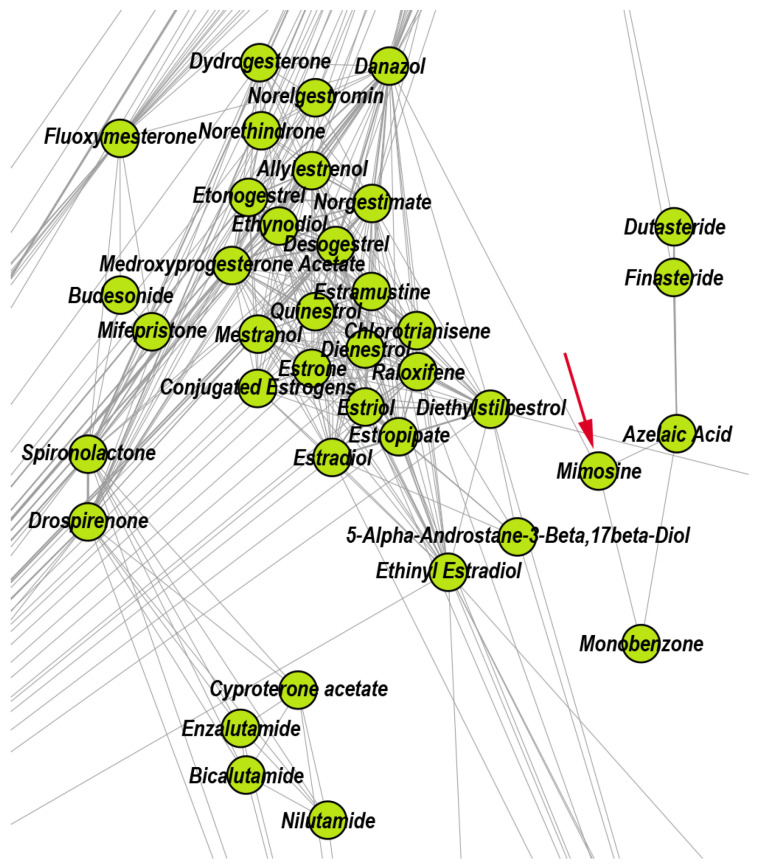
Zoomed DDSN detail of community $C_{6}$ (Drugs interfering with hormone-dependent cancers). The red arrow indicates the reconstructed drug repositioning: Mimosine—an experimental antineoplastic that inhibits DNA replication—also has effects in cancers affecting hormone-dependent organs.

Quinacrine is an antiprotozoal drug that exhibits an anticancer effect in breast cancer because it produces apoptosis by blocking cells in S-phase, induces DNA damage, and inhibits topoisomerase activity [58]; indeed, reference [59] recommends the clinical trial test of Quinacrine for the treatment of patients with androgen-independent prostate cancer. The antineoplastic drug Mimosine attenuates cell proliferation of prostate carcinoma cells in vitro [60]. Figure 5 provides a zoomed detail (i.e., focused view) of the DDSN that highlights Mimosine’s presence (an experimental antineoplastic which inhibits DNA replication) in $C_{6}$; this indicates that Mimosine has effects in hormone-dependent cancers.

#### 3.2.2. Reconstructed Repurposings as Anti-Inflammatory Drugs

**Figure 6:**
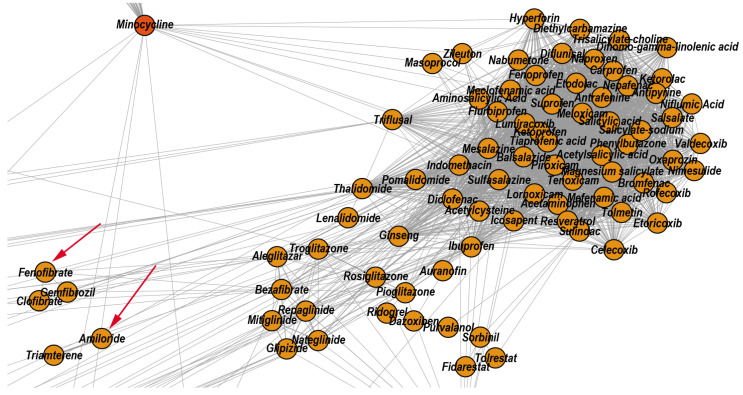
Zoomed DDSN detail of community $C_{3}$ (Anti-inflammatory drugs). The red arrows indicate the reconstructed drug repositionings as anti-inflammatory drugs: Fenofibrate (a lipid modifying drug) and Amiloride (a diuretic).

According to the properties listed in DrugBank, the topological community $C_{3}$ includes drugs that exert anti-inflammatory effects via different mechanisms: non-steroidal anti-inflammatory drugs (e.g., Diclofenac, Ibuprofen, and Acetylsalicylic acid), the antirheumatic agent Auranofin, hypoglycemic drugs (e.g., Rosiglitazone, Troglitazone), and the antihypertensive drug Telmisartan. Moreover, the literature confirms that 28.57% of drugs within this community also present anti-inflammatory effects, even if they are not listed as anti-inflammatories in DrugBank. Here, we present the example of the versatile molecule of Fenofibrate, which reduces the systemic inflammation independent of its lipid regulation effects, with cardiovascular benefits in high-risk [61] and rheumatoid arthritis patients [62]. Another illustrative example is that of Amiloride, which inhibits the activation of the dendritic cells and ameliorates the inflammation besides its diuretic effects, thus having benefits for hypertensive patients [63]. Figure 6 shows a zoomed DDSN detail, highlighting the presence of Fenofibrate and Amiloride in $C_{3}$; this may indicate that the highlighted drugs also have anti-inflammatory effects.

#### 3.2.3. Reconstructed Repurposings as Antifungal Drugs

**Figure 7:**
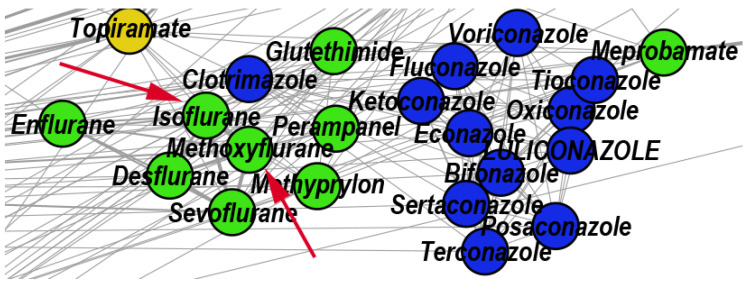
Zoomed DDSN detail of community $C_{25}$ (Antifungal agents). The red arrows indicate the reconstructed drug repositionings: Isoflurane and Methoxyflurane (known as general anesthetic drugs) also have antifungal effects.

The topological community $C_{25}$ includes 22 drugs. According to DrugBank, 13 out of these 22 drugs have antifungal properties, and 9 drugs act on the central nervous system (i.e., general anesthetics, sedative-hypnotics, and antiepileptics). DrugBank lists Isoflurane and Methoxyflurane as general anesthetic drugs. However, A. Giorgi et al. performed in vitro tests to investigate the antibacterial and antifungal effects of common anesthetic gases, and they found that Methoxyflurane and Isoflurane have excellent inhibitory effects on cultures of Klebsiella pneumoniae and Candida albicans [64]. Using in vitro experiments, V.M. Barodka et al. also found that Isoflurane’s liquid formulation has better anti-Candida activity than the antifungal Amphotericin B [65]. Figure 7 shows a zoomed DDSN detail highlighting the presence of Isoflurane and Methoxyflurane in $C_{25}$; this indicates that the highlighted drugs may also have antifungal effects.

### 3.3. Repositioning Hints Prioritization

**Figure 8:**
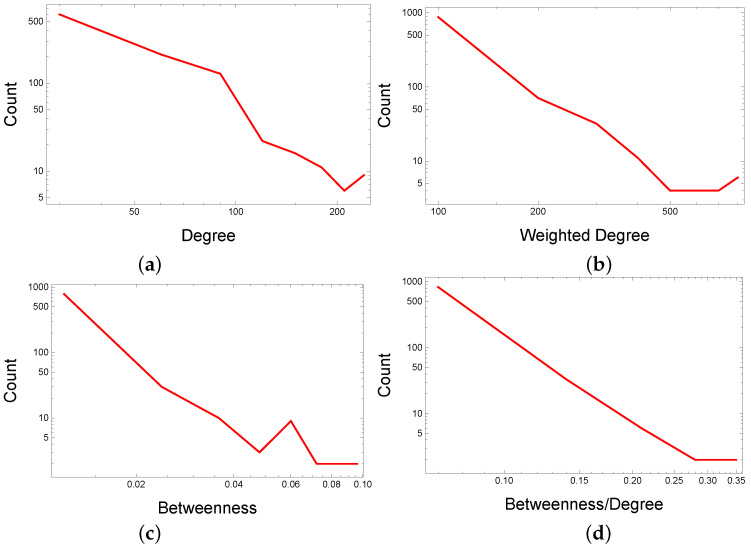
Power-law distributions of centrality parameters in the drug–drug similarity network (DDSN): (**a**) degree *d*; (**b**) weighted degree $d_{w}$; (**c**) betweenness *b*, and (**d**) betweenness/degree $\frac{b}{d}$. According to the guidelines in [67], we represent the distributions using 8 linearly spaced bins for each centrality. The fitting analysis using the Powerlaw package in Python [67] indicates the following values for the distribution slope α and cutoff point $x_{min}$, respectively: 3.436 and 53 for *d*, 2.598 and 64 for $d_{w}$, 2.201 and 0.008 for *b*, 3.093 and 0.088 for $\frac{b}{d}$. The graphical representations of these centrality distributions show that the betweenness/degree $\frac{b}{d}$ is the most stable parameter; therefore, it is the most reliable indicator of multiple drug properties.

A high degree node represents a drug with already documented multiple properties in our characterization of drug–drug similarity networks. Furthermore, a high betweenness (i.e., the ability to connect network communities) indicates the drug’s propensity for multiple pharmacological functions. By this logic, the high-betweenness, high-degree nodes may have reached their full repositioning potential, whereas the high betweenness, low degree nodes (characterized by high betweenness/degree value $\frac{b}{d}$) may indicate a significant repositioning potential. However, predicting such high-value cases of degree *d*, weighted degree $d_{w}$, betweenness *b*, and betweenness/degree $\frac{b}{d}$ is difficult because the corresponding distributions are fat-tailed [66]. Although all the estimated DDSN centralities follow a power-law distribution (see Figure 8), the betweenness/degree $\frac{b}{d}$ is the most stable parameter and, hence, the most reliable indicator of multiple drug properties.

To explore the capability of $\frac{b}{d}$ to predict the multiple drug properties, we exploit the community structure of DDSN by following a two-step approach.

1. We uncover the relevant drug properties by generating network communities $C_{x}$ with $x=\bar{1,m}$ (m=26 in our DDSN). Then, using expert analysis, we assign a dominant property to each community. Figure 3 illustrates the 26 DDSN communities as well as their dominant functionality. The dominant community property can be a pharmacological mechanism, a targeted disease, or a targeted organ. For instance, the community 1 ($C_{1}$) consists of antineoplastic drugs which act as mitotic inhibitors and DNA damaging agents; Community 13 ($C_{13}$) consists of cardiovascular drugs (antihypertensive, anti-arrhythmic, and anti-angina drugs), mostly beta-blockers.
2. In each cluster $C_{x}$, we identify the top *t* drugs according to their $\frac{b}{d}$ values. From these selected drugs, $B_{x}^{t}\subset C_{x}$, some stand out by not sharing the community property or properties, and thus, can be repositioned as such. To this end, for $x=\bar{1,m}$ eliminated from $B_{x}^{t}$ the drugs whose repurposings were already confirmed (i.e., performed by others and found in the recent literature), thus producing m=26 lists of repurposing hints yet to be confirmed by in silico, in vitro, and in vivo experiments, $B_{x}^{h}=B_{x}^{t}\backslash B_{x}^{c}$. Table 3 presents the lists of $B_{x}^{t}$ drugs for t=5 and x=26 (i.e., the top 5 $\frac{b}{d}$ drugs in each community). We chose t=5 to provide a reasonable amount of eloquent information in Table 3; we provide the entire $B_{x}$ sets in the Supplementary file SupplementaryDDSN.

**Figure 9:**
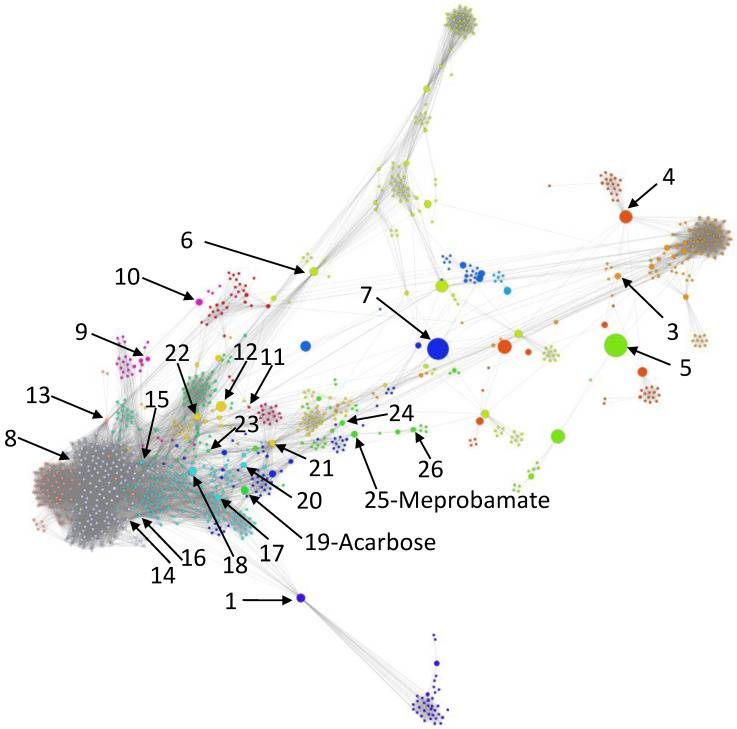
Drug–drug similarity network (DDSN), based on drug–target interactions, where node sizes represent their $\frac{b}{d}$ values. The arrows indicate the top $\frac{b}{d}$ node in each community (for community 2, there is no top node because all drugs have $\frac{b}{d}=0$). The community index identifies each top $\frac{b}{d}$ node, excepting Meprobamate (top $\frac{b}{d}$ in community 25) and Acarbose (community 19), because these drugs (apparently) do not have their community’s property; this indicates Meprobamate as antifungal (i.e., the property of community 25) and Acarbose as antiarrhythmic, anticonvulsant (i.e., the properties of community 19).

To facilitate the visual identification of the repositioning hints, in Figure 9, we shape the size of the nodes of our DDSN representation according to the magnitude of the $\frac{b}{d}$ values. By arrows, we also identify the top $\frac{b}{d}$ nodes (i.e., drugs) in their respective communities, by indicating their community id. Table 3 shows that $B_{x}^{1}=\emptyset$ for all *x* except 19 and 25 ($B_{19}^{1}=\left\{\mathrm{Acarbose}\right\}$ and $B_{25}^{1}=\left\{\mathrm{Meprobamate}\right\}$), therefore—besides the corresponding community number—we expressly point Acarbose and Meprobamate in Figure 9.

The high percentage of database and literature confirmations of our pharmacological properties predictions highlight the robustness of our repurposing method. In the Supplementary file SupplementaryDDSN, we show that the confirmation rate $\sum_{x}B_{x}^{c}/\sum_{x}C_{x}$ = 86.51%. Table 3 presents a similar situation, with only a few unconfirmed drug properties (these repurposing hints $\in B_{x}^{h}$ are in bold).

Our data indicate two top $\frac{b}{d}$ drugs: Meprobamate, in the $C_{25}$ antifungal drugs community, and Acarbose, in the $C_{19}$ (Antiarrhythmics and Anticonvulsants) community. Both repositionings refer to properties currently unaccounted in the DrugBank version 5.1.4 and the scientific literature we have screened (Table 3 and Figure 9). Meprobamate is a hypnotic, sedative, and mild muscle-relaxing drug, with no reported activities on the antifungal drug targets; thus, the antifungal activities of Meprobamate are not yet investigated in silico (with molecular docking), in vitro, or in vivo. Acarbose is a hypoglycemic drug, with no reported nor investigated antiarrhythmic and anticonvulsant properties. At the same time, one should also consider repurposing hints for drugs with high $\frac{b}{d}$, when the highest $\frac{b}{d}$ values correspond to drugs already confirmed with the community property. For example, Azelaic acid has the highest $\frac{b}{d}$ across not confirmed drugs in $C_{6}$.

### 3.4. Repurposing Hints Testing

Molecular docking uses the target and ligand structures to predict the lead compound or repurpose drugs for different therapeutic purposes. The molecular docking tools predict the binding affinities, the preferred poses, and the ligand-receptor complex’s interactions with minimum free energy. In this paper, we use the AutoDock 4.2.6 software suite [46], which consists of automated docking tools for predicting the binding of small ligands (i.e., drugs) to a macromolecule with an established 3D structure (i.e., target). The AutoDock semi-empirical free energy force field predicts the binding energy by considering complex energetic evaluations of bound and unbound forms of the ligand and the target, as well as an estimate of the conformational entropy lost upon binding.

According to the methodology in Section 2 (Section 2.3), we verify the predicted properties of repurposing hints by performing molecular docking not only for the hinted drugs but also for the reference drugs (typical drugs having the predicted property) and for some drugs with little probability of having the expected property. This way, we facilitate the comparison between the interaction of the hinted drug with the biological targets—relevant for the tested property—and the interactions of the reference drugs with the same targets.

Following the methodology in Section 2 (Section 2.3), we first consider the property ϕ as the anticancer effect with x=6 (corresponding to community $C_{6}$), and second ϕ as the antifungal effect with x=25 (community $C_{25}$). As such, we test the repurposing hints $D_{h}^{\phi }=D_{h}^{\mathrm{anticancer}}=\left\{\mathrm{Azelaic}\;\mathrm{acid}\right\}$ and $D_{h}^{\phi }=D_{h}^{\mathrm{antifungal}}=\left\{\mathrm{Meprobamate}\right\}$.

Accordingly, we define the anticancer reference drug from $C_{6}$ as $D_{6}^{\mathrm{antifungal}}$ = {Progesterone, Abiraterone}, no anticancer reference drug outside $C_{6}$ (i.e., $D_{\bar{6}}^{\mathrm{anticancer}}=\emptyset$), and two reference drugs with a low probability of anticancer effects $D_{n}^{\mathrm{anticancer}}=\left\{\mathrm{Fosinopril},\mathrm{Furosemide}\right\}$ (Fosinopril is an antihypertensive and Furosemide is a diuretic). Here, we test the interaction between the hinted and reference drugs with the targets from DrugBank associated with anticancer drugs in $C_{6}$, namely $T_{6}^{\mathrm{antifungal}}$ = {Progesterone receptor, Androgen receptor, Estrogen receptor beta, Steroid 17-alpha-hydroxylase/17,20 lyase, Mineralocorticoid receptor, Estrogen receptor alpha}.

Similarly, we consider the antifungal references in $C_{25}$ as $D_{25}^{\mathrm{antifungal}}=\left\{\mathrm{Clotrimazole},\mathrm{Oxiconazole}\right\}$, and outside $C_{25}$ as $D_{\bar{25}}^{\mathrm{antifungal}}=\left\{\mathrm{Naftifine},\mathrm{Tolnaftate},\mathrm{Nystatin},\mathrm{Natamycin},\mathrm{Ciclopirox},\mathrm{Griseofulvin}\right\}$. The reference drugs with little probability of having antifungal properties are $D_{n}^{\mathrm{antifungal}}=\left\{\mathrm{Fosinopril},\mathrm{Furosemide}\right\}$. We test the interactions between the hinted and reference drugs with DrugBank antifungal-related targets linked to drugs in $C_{25}$ and drugs not in $C_{25}$, respectively $T_{25}^{\mathrm{antifungal}}$ = {Lanosterol 14-alpha demethylase, Lanosterol synthase, Intermediate conductance calcium-activated potassium channel protein 4}, and $T_{\bar{25}}^{\mathrm{antifungal}}$ = {Squalene monooxygenase, Ergosterol, Sodium/potassium-transporting ATPase subunit alpha, Tubulin}.

**Figure 10:**
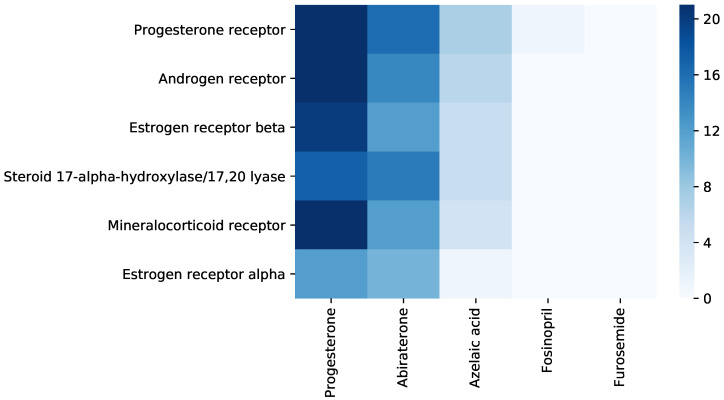
Synthesis of interactions resulted from running molecular docking on the drug–target pairs for $D_{h}^{\phi }=D_{h}^{\mathrm{anticancer}}=\left\{\mathrm{Azelaic}\;\mathrm{acid}\right\}$. In the left part of the heatmap, we present the interactions between the relevant targets $T^{\mathrm{anticancer}}=$ {Progesterone receptor, Androgen receptor, Estrogen receptor beta, Steroid 17-alpha-hydroxylase/17,20 lyase, Mineralocorticoid receptor, Estrogen receptor alpha}, and the reference drugs $D_{r}^{\mathrm{anticancer}}=$ {Progesterone, Abiraterone}. In the right part of the heatmap, we present the interactions between the relevant targets $T^{\mathrm{anticancer}}$ and the tested drugs $D_{t}^{\mathrm{anticancer}}=$ {Azelaic acid, Fosinopril, Furosemide}). We summarize the interactions with the targets $T^{\mathrm{anticancer}}$ as the number of amino acids from the target interacting with the drug molecule (from 0 to the maximum number in our experiments, namely 21). The heatmap representation indicates interactions between $d_{h}^{\mathrm{anticancer}}=$ {Azelaic acid} and almost all the targets from $T_{6}^{\mathrm{anticancer}}$. For the drugs in $D_{n}^{\mathrm{anticancer}}$, namely Fosinopril and Furosemide, there is no interaction with the targets from $T_{6}^{\mathrm{anticancer}}$.

Figure 10 shows the summary of interactions resulted from the molecular docking analysis of the drug–target pairs generated with Equations (11)–(13) (Section 2, Section 2.3.1) for the hint $D_{h}^{\mathrm{anticancer}}=\left\{\mathrm{Azelaic}\;\mathrm{acid}\right\}$. For the hint and the reference drugs $D_{r}^{\mathrm{antifungal}}$, we represent the interactions with the targets $T^{\mathrm{antifungal}}$ as the number of amino acids from the target interacting with the drug molecule (the maximum is 21). We provide the details related to the molecular docking simulations in the pdf Supplementary Information—Tables S1–S6 and Figures S1–S6.

**Figure 11:**
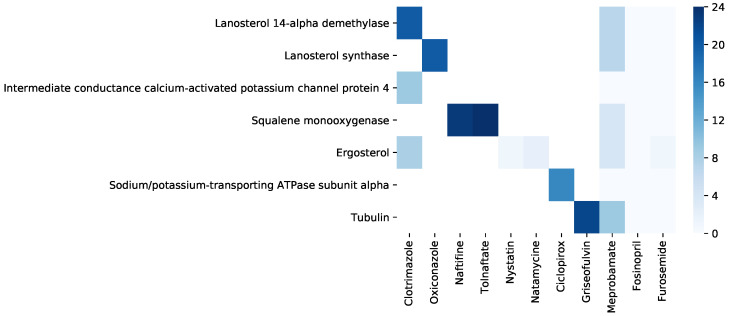
Synthesis of interactions resulted from running molecular docking on the drug–target pairs for $D_{h}^{\phi }=D_{h}^{\mathrm{antifungal}}=\left\{\mathrm{Meprobamate}\right\}$. In the left part of the heatmap, we present the interactions between the relevant targets $T^{\mathrm{antifungal}}=$ {Lanosterol 14-alpha demethylase, Lanosterol synthase, Intermediate conductance calcium-activated potassium channel protein 4, Squalene monooxygenase, Ergosterol, Sodium/potassium-transporting ATPase subunit alpha, Tubulin} and the reference drugs $D_{r}^{\mathrm{antifungal}}=$ {Clotrimazole, Oxiconazole, Naftifine, Tolnaftate, Nystatin, Natamycin, Ciclopirox, Griseofulvin}. We only test the reference drugs and targets pairs that interact according to DrugBank; all the other pairs are white in our representation because they are not tested. In the right part of the heatmap, we present the interactions between the relevant targets $T^{\mathrm{antifungal}}$ and the tested drugs $D_{t}^{\mathrm{antifungal}}=$ {Meprobamate, Fosinopril, Furosemide}). We summarize the interactions with the targets $T^{\mathrm{antifungal}}$ as the number of amino acids from the target interacting with the drug molecule (from 0 to the maximum number in our experiments, namely 24). In the case of Ergosterol $\in T_{\bar{25}}^{\mathrm{antifungal}}$, instead of the number of amino acids, we count the number of hydrophobic alkyl/alkyl interactions because this target has a steroidal chemical structure. The heatmap representation indicates interactions between $d_{h}^{\mathrm{antifungal}}$ (i.e., Meprobamate) and almost all the targets from both $T_{25}^{\mathrm{antifungal}}$ and $T_{\bar{25}}^{\mathrm{antifungal}}$. For the drugs in $D_{n}^{\mathrm{antifungal}}$, there is no relevant interaction with any target from $T_{25}^{\mathrm{antifungal}}⋃T_{\bar{25}}^{\mathrm{antifungal}}$.

Figure 11 presents the summary of interactions resulted from the molecular docking analysis of the drug–target pairs generated with Equations (11)–(13) (see Section 2, Section 2.3.1), for the hint $D_{h}^{\phi }=D_{h}^{\mathrm{antifungal}}=\left\{\mathrm{Meprobamate}\right\}$. For the reference drugs $D_{r}^{\mathrm{antifungal}}=$ {Clotrimazole, Oxiconazole, Naftifine, Tolnaftate, Nystatin, Natamycin, Ciclopirox, Griseofulvin} the interaction is represented as the number of amino acids in the target interacting with the drug molecule (the maximum in our molecular docking experiments is 24). Because Ergosterol $\in T_{\bar{25}}^{\mathrm{antifungal}}$ has a steroidal chemical structure, instead of the number of amino acids, we represent interaction strength as the number of hydrophobic alkyl/alkyl interactions. For the tested drugs $D_{t}^{\mathrm{antifungal}}=$ {Meprobamate, Fosinopril, Furosemide}, we represent the interaction as the number of amino acids from the target (or hydrophobic alkyl/alkyl interactions for Ergosterol) interacting in the same way with both the tested drug ($\in D_{t}^{\mathrm{antifungal}}$) and at least one reference drug ($\in D_{25}^{\mathrm{antifungal}}$). We provide a detailed description of the molecular docking simulations for Meprobamate in the pdf Supplementary Information—Tables S7–S13 and Figures S7–S13. The results confirm the interactions between $d_{h}^{\mathrm{antifungal}}$ (i.e., Meprobamate) and almost all the targets from both $T_{25}^{\mathrm{antifungal}}$ and $T_{\bar{25}}^{\mathrm{antifungal}}$. Conversely, for the drugs in $D_{n}^{\mathrm{antifungal}}$, there is no relevant interaction with any target from $T_{25}^{\mathrm{antifungal}}⋃T_{\bar{25}}^{\mathrm{antifungal}}$.

After Autodock 4.2.6 and AutoDock Vina redocking according to the procedure in Section 2.3.3, we calculate RMSD in both cases. We obtain low RMSD values (i.e., all of them are ≤1.016 Å), suggesting that our preliminary docking methodology is robust [68] (details in Suplementary Information .pdf file, Section S6).

## 4. Discussion

Drug repurposing represents a promising strategy to accelerate drug discovery in sensitive areas of nowadays medicine, such as antibacterial resistance, complex life-threatening diseases (e.g., cancer), or rare diseases. In this paper, we describe a novel weighted drug–drug similarity network whose weights encode the existing known relationships among drugs (i.e., quantifies the number of biological targets shared by two drugs irrespective of the agonist or antagonist effect).

We then demonstrate that the ratio between node betweenness and node degree (i.e., a criterion of combined network metrics) can indicate the drug repositioning candidates better than considering simple network metrics (e.g., degree, weighted degree, betweenness). Indeed, the power-law distributions in Figure 8 suggest that our DDSN is a complex system; thus, the conventional statistical analysis of the DDSN can be misleading. Consequently, we introduce a different approach to deciphering the emerging hidden higher-order functional interactions (i.e., interactions that span multiple orders of magnitude and involve multiple nodes) by visualizing and analyzing the community structure in DDSN and determining the culprits (for such unknown functionalities) through combined network metrics criterion. We use the force-directed energy layout Force Atlas 2 to generate network clusters of drugs [34] because it emulates the emerging processes of a complex system. More precisely, the force-directed based network layouts use micro-scale interactions (i.e., adjacent nodes attract and non-adjacent nodes repulse) to generate an emergent behavior at the macro-scale (i.e., topological clusters). Once we identify communities, the combined network metrics criterion selects the drug repositioning most likely candidates. Specifically, our weighted drug–drug network analysis encodes not only information about how pairs of drugs interact with biological targets but also reveals the unknown functional relationship between drugs, such as the unknown effects on the activation/inhibition of a chemical pathway or cellular behavior. We used a similar methodology—underpinned by force-directed layout clustering—to analyze the fundamentally different structures represented by the drug–drug interaction networks (i.e., the DDIN interactome [48,69]).

### 4.1. Complex Network Perspective

When analyzing networks built with drug data, one must be aware and carefully deal with data incompleteness. Mestres et al. acknowledged this problem for networks built with data from the 2006 DrugBank version, where drug–target data scarcity was indeed a problem [70]. However, in this paper, we worked on a much more comprehensive database, with a much larger and denser number of nodes/drugs and connections. Still, even if recent years’ research alleviated the data scarcity problem, any network analysis has to consider a degree of entailed uncertainty.

Another important aspect of our method’s data processing is the interpretation of b/d ranking. We chose this composite centrality because its distribution in DDSN is more stable than other centralities; therefore, as also suggested by [71,72], it should produce more robust rankings. However, reliable confirmation of b/d as an efficient priority indicator requires retrospective in vivo, in vitro, and in silico (i.e., molecular docking) experiments, and we encourage future research in this way.

We select Azelaic acid (saturated dicarboxylic acid) and Meprobamate (carbamate derivative) as possible antineoplastic and antifungal from our repurposing hints list, respectively. Even so, one may find a posteriori confirmation clues for such repositioning hints. For instance, in [73], the authors discuss the antitumoral effects of Azelaic acid in the case of melanoma and only hypothesize that it may be tested in hormone-related cancers. Furthermore, the Meprobamate molecule contains a moiety that can be associated with antifungal effects [74]. However, these associations only make sense because our DDSN analysis orients this process. Moreover, in the docking experiments, the two hints are not structurally similar to the respective reference drugs (i.e., Progesterone and Abiraterone for antineoplastic, and Clotrimazole, Oxiconazole, Naftifine, Tolnaftate, Nystatin, Natamycin, Ciclopirox, Griseofulvin for antifungal). Indeed, Progesterone and Abiraterone are steroid derivatives, Clotrimazole and Oxiconazole are imidazole derivatives, Ergosterol has a steroidal structure, Terbinafine and Naftifine are allylamine compounds, Griseofulvin is a 3-coumaranone derivative.

### 4.2. Molecular Docking Perspective

Molecular docking represents an alternative, in silico simulation approach to drug discovery, which models the physical interaction between a ligand (i.e., small drug molecule) and a macromolecule (e.g., synthetic host macromolecule, biological target) [75]; it is also a valuable repurposing tool [68,76]. We estimate the free energy values of the molecular interactions with molecular docking to offer a good approximation for the ligand’s conformation and orientation into the protein cavity [77]. DOCK [78] is a dedicated software tool used in drug repurposing along with many available molecular docking models. For example, R. L. Des Jarlais et al. used the Dock computer algorithm to find that haloperidol inhibits HIV-1 and HIV-2 proteases [79]. However, molecular docking can not work unless we have some strong repositioning hints; otherwise, the search space for drug repositionings would be exponentially big. To this end, the methodology proposed in this paper provides strong drug–target interaction hints, such that we can build large-scale drug–target interaction profiles [8,80]. Our approach integrates the molecular docking with complex networks to hint new pharmacological properties by identifying new sets of biological targets on which the drug acts. However, in this paper, we performed only a preliminary docking testing, as our primary focus is the network-based repurposing approach. As such, we recommend that future, more focused, research continue our docking simulations by including target baits (to reflect the limitations of false-positive and false-negative results), considering solvent effects, flexible docking, and comparing multiple docking tools. To this end, we indicate the robust docking methodologies employed in [68,81,82,83].

As Yvonne Martin et al. indicated [84], the paradigm of chemical similarity—which holds that structurally similar drug molecules exert similar biological effects—cannot fully explain drugs’ biological behavior. They found that only 30% of compounds similar to a particular active compound are themselves active (the compounds are structurally similar if the Tanimoto coefficient is ≥0.85 in the Daylight fingerprints). Therefore, behavioral approaches can successfully complement the structural paradigm. To this end, similar interaction profiles are valuable resources in drug repurposing, as drugs with similar target binding patterns may exhibit a similar pharmacologic activity [80,85,86]. As the chemical similarity is not necessarily a reliable predictor of biological similarity [84,87], we analyze the binding modes of Azelaic acid and Meprobamate compared to the other known reference drugs (see Sections S3–S5 in the pdf Supplementary Information).

We highlight the docking simulation results for the interaction between Azelaic acid and Steroid 17-alpha-hydroxylase/17,20 lyase, highly similar to Progesterone and Abiraterone interactions with this target (see pdf Supplementary Information, Table S4). Abiraterone is a potent 17-alpha-hydroxylase/17,20-lyase inhibitor used for the treatment of androgen-dependent prostate cancer [37]. Therefore, discovering new drugs that inhibit this enzyme is a logical strategy. However, because steroidal drugs—such as Abiraterone—have multiple steroid-related side effects, Hille et al. decided to synthesize non-steroidal compounds that mimic the natural 17-alpha-hydroxylase/17,20-lyase substrates (i.e., pregnenolone and progesterone) [88]. Our docking simulation results are in line with references [89,90], which report the covalent bonding of Abiraterone to Steroid 17-alpha-hydroxylase/17,20 lyase (a cysteinato-heme enzyme from the cytochrome P450 superfamily). Precisely, Abiraterone forms a coordinate covalent bond of the pyridine nitrogen at C17 with this target’s heme iron [90]. Furthermore, our docking simulation of the interaction between Abiraterone and 17-alpha-hydroxylase/17,20-lyase confirms that Abiraterone establishes a hydrogen-bond between the -OH group and the target’s Asn202; our results also confirm that amino acid residues of Phe114, Ile206, Leu209, Arg239, Gly301, and Val482 represent the hydrophobic environment for the reference Abiraterone [91]. According to our docking simulation results, Azelaic acid does not establish a hydrogen bond with Asn202; however, not all the inhibitors tested by Chun-Zhi Ai et al. form a hydrogen bond with Asn202. (Instead, they bond to other amino acid residues than Abiraterone [91].) In a recent paper, Gabriele Micheletti et al. reported results of biological and docking evaluations of some hybrid aza-heterocycles compounds, which bound azelayl moiety through an amide bond that act as histone deacetylase inhibitors; this suggests the anticancer potential for three of their Azelaic acid derivatives in osteosarcoma among the five tumor cell lines tested [92].

Meprobamate has similar binding modes to that of Clotrimazole with Lanosterol 14 alpha-demethylase, Oxiconazole with Lanosterol synthase, and Griseofulvin with Tubulin. Indeed, we find the carbamate moiety in a wide range of drugs, such as Felbamate (anticonvulsant), Disulfiram (the treatment of chronic alcoholism), Rivastigmine (anti-dementia), Darunavir (antiviral for the treatment of HIV infections), or Physostigmine (antiglaucoma). Furthermore, carbamates are reversible acetylcholinesterase inhibitors that act as effective fungicides, insecticides, and herbicides in agriculture [74]. Indeed, a recent reference reports the synthesis, in vitro, and in vivo antifungal evaluation of 36 novel threoninamide carbamate derivatives using the pharmacophore model [93].

## 5. Conclusions

The overarching conclusion is that our network-based computational drug repurposing method is robust, as it recovers a wide array of previous drug repositionings. We prove such robustness by employing our approach on an older database, to validate the results with a new DrugBank version. Nonetheless, in drug repositioning, we deal with unknown unknowns; thus, we need to consider the seemingly unconfirmed drug properties as potential repurposing hints. Testing all these hints is a daunting task that requires vast resources; thus, we propose a testing prioritization method based on network centralities.

In this paper, we started a preliminary validation of previously unaccounted drug properties using molecular docking. As such, we find that the Azelaic acid represents a promising candidate for further in silico (e.g., molecular dynamics), in vitro, and in vivo investigations of its potential anticancer effects. Although the molecular docking results are not as strong as for Azelaic acid, Meprobamate’s antifungal properties cannot be disregarded or rejected. Meprobamate is a known oral drug; however, we cannot exclude the topical administration route as an antifungal. To this end, we need further investigations on biopharmaceutical properties to test various pharmaceutical topical formulations with Meprobamate as an active ingredient. The same discussion on the biopharmaceutical properties is valid for Azelaic acid, knowing that its administration route may change as an anticancer drug.

Our findings pave the way for further employing the target-based drug–drug similarity networks with the latest available drug–target interaction data, as well as for in vitro and in silico experiments that will eventually establish useful drug repositionings.

## Figures and Tables

**Table 1:** Confirmation of drug community properties and drug repurposing hints. Each table line corresponds to a topological community $C_{x}$ (with $x=\bar{1..15}$), by specifying the dominant property (or properties) resulted from the pharmacological analysis (column Properties), the number of nodes/drugs in community $C_{x}$ (column Nodes [#]), the percentage of drugs with the properties confirmed by DrugBank (column DrugBank [%]), the percentage of drugs with the predicted properties confirmed by the literature (column Literature [%]), the percentage of drugs with not yet confirmed predicted properties (column Not confirmed [%]), and the drugs we propose for repositioning, representing predictions not confirmed yet but with non-zero betweenness/degree in the DDSN (b/d>0, in column Hints).

$C_{x}$	Properties	Nodes [#]	DrugBank [%]	Literature [%]	Not Confirmed [%]	Hints
1	Antineoplastic (mitotic inhibitors and DNA-damaging)	37	40.54	37.84	21.62	Besifloxacin Pefloxacin Norfloxacin Ofloxacin
2	Antihypertensive (sartans)	10	100	0	0	–
3	Anti-inflammatory	84	65.48	28.57	5.95	Glipizide
4	Antibacterial tetracyclines and Aminoglycosides	20	95.00	0	5.00	Plerixafor
5	Platelet aggregation inhibitor	29	10.34	82.76	6.90	–
6	Interfering with hormone-dependent cancers	93	26.88	65.59	7.53	Azelaic ac.
7	Anticancer (molecularly targeted)	92	23.91	50.00	26.09	Suramin Acetohydroxamic ac. Glyburide Gliquidone Tolbutamide
8	Anti-allergic	51	86.27	11.76	1.96	Butriptyline
9	Acting on muscles	25	72.00	16.00	12.00	–
10	Vasodilator	37	48.65	24.32	27.03	Tofisopam Mefloquine Oxtriphylline Enprofylline Roflumilast Aminophylline
11	Antiepileptic, hypnotic, and sedative	19	84.21	10.53	5.26	Barbituric ac. deriv.
12	Analgesic and used in opiate withdrawal & side-effects	46	89.13	8.70	2.17	–
13	Antihypertensive, anti-arrhythmic, anti-angina (mostly beta-blockers)	26	92.31	3.85	3.85	–
14	Anticholinergic	53	100	0	0	–
15	Interfering with Parasympathetic Nervous System	97	42.27	42.27	15.46	Doxazosin Terazosin Prazosin Paliperidone Aripiprazole Fenoldopam Dapiprazole Alfuzosin Tamsulosin Silodosin Amisulpiride Carphenazine Acetophenazine

**Table 2:** Confirmation of drug community properties and drug repurposing hints. Each table line corresponds to a topological community $C_{x}$ (with $x=\bar{16..26}$), as well as the last line for the entire DDSN, by specifying the dominant property (or properties) resulted from the pharmacological expert analysis (column Properties), the number of nodes/drugs in community $C_{x}$ (column Nodes [#]), the percentage of drugs with the properties confirmed by DrugBank (column DrugBank [%]), the percentage of drugs with the predicted properties confirmed by the literature (column Literature [%]), the percentage of drugs with not yet confirmed predicted properties (column Not confirmed [%]), and the drugs we propose for repositioning, representing predictions not confirmed yet but with non-zero betweenness/degree in the DDSN (b/d>0, in column Hints).

$C_{x}$	Properties	Nodes [#]	DrugBank [%]	Literature [%]	Not Confirmed [%]	Hints
16	Antidepressant and Central Nervous System stimulant	26	92.31	7.69	0	–
17	Sympathetic Nervous System acting	61	85.25	8.20	6.56	–
18	Antimigraine and antiemetic	26	42.31	26.92	30.77	Captodiame Ropinirole MDMA Dofetilide Rotigotine L-DOPA
19	Antiarrhythmic and anticonvulsant	24	66.67	12.50	20.83	Acarbose Hexylcaine
20	Antidepressant and anti-Parkinson	21	57.14	14.29	28.57	Quinidine Propafenone Cinchocaine MMDA Aprindine
21	Interfering with epilepsy and blood pressure	12	41.67	25.00	33.33	Miconazole Quinidine barbiturate
22	Antihypertensive and anticonvulsant	20	80.00	15.00	5.00	–
23	Anesthetic, analgesic, and muscle relaxant	19	73.68	5.26	21.05	Halofantrine Ibutilide Pentolinium
24	Interfering with K, Na, Ca homeostasis	51	50.98	13.73	35.29	Progabide Bethanidine Ellagic ac. Vigabatrin Ethinamate
25	Antifungal	22	59.09	9.09	31.82	Meprobamate Enflurane Sevoflurane Desflurane
26	Hypnotic and sedative	7	100	0	0	–
All	–	1008	59.52	26.98	13.49	–

**Table 3:** Top 5 drugs ($B_{x}^{5}$ with $x=\bar{1,26}$) according to their $\frac{b}{d}$ values, for each of the 26 DDSN communities/clusters ($C_{x}$). The properties of drugs written in regular fonts match the properties of their respective communities (according to the DrugBank). The properties of italicized drugs do not match all their respective communities’ properties, but the latest literature confirms them (drugs in regular fonts and italics pertain to $B_{x}^{h}$). The properties of the drugs written in bold do not match the community properties, and the literature did not confirm them yet; this situation leads to new drug repositioning hints (i.e., the $B_{x}^{h}$ drugs). The positions marked with ‘–’ correspond to drugs with $\frac{b}{d}=0$.

	$B_{x}^{5}$	1	2	3	4	5
$C_{x}$						
1		Amsacrine	Colchicine	Podofilox	Lucanthone	**Besifloxacin**
2		–	–	–	–	–
3		Amiloride	Marimastat	Diclofenac	Thalidomide	Telmisartan
4		Minocycline	Framycetin	Amikacin Tobramycin Netilmicin	Doxycycline Clomocycline Oxytetracycline	–
5		Treprostinil	Iloprost	Captopril	Bimatoprost	Candoxatril
6		Progesterone	Mimosine	Fluticasone propionate	Danazol	Spironolactone
7		Vandetanib	Dalteparin	Dehydroepiandrosterone	Amlexanox	Atorvastatin
8		Olopatadine	Terfenadine	Flunarizine	Astemizole	Epinastine
9		Succinylcholine	Carbachol	Decamethonium	Pilocarpine	Cevimeline
10		Nicotine	Melatonin	Amrinone	Dipyridamole	Naloxone
11		Quinine	Phenobarbital Secobarbital Pentobarbital	Barbital Hexobarbital Aprobarbital	–	–
12		Nimodipine	Adenosine	Drotaverine	Pentazocine	Loperamide
13		Ketotifen	Amiodarone	Sotalol	Bevantolol	Penbutolol
14		Disopyramide	Scopolamine	Ethopropazine	Paroxetine	Rocuronium
15		Minaprine	Amitriptyline	Agomelatine	Orphenadrine	Imipramine
16		Cocaine	Chloroprocaine	Procaine	Phenermine	Milnacipran
17		Epinephrine	4-Methoxyamphetamine	Pseudoephedrine	Ephedra	Methamphetamine
18		Ginkgo biloba	**Captodiame**	Cisapride	Bromocriptine	Carteolol
19		**Acarbose**	Lidocaine	Mexiletine	Etomidate	Flecainide
20		Phenelzine	Agmatine	**Quinidine** **Propafenone**	Ephedrine	Amphetamine
21		Zonisamide	**Miconazole**	Ethanol	**Quinidine barbiturate**	–
22		Felodipine	Bepridil	Verapamil	Dextromethorphan	Amlodipine
23		Halothane	**Halofantrine**	Tramadol	Ibutilide	Tubocurarine
24		Thiamylal	Valproic Acid	**Progabide**	**Bethanidine**	Topiramate
25		**Meprobamate**	**Enflurane**	Tioconazole	Clotrimazole	Methoxyflurane **Isoflurane** **Sevoflurane**
26		Flunitrazepam	Eszopiclone	–	–	–

## Supplementary Materials

The following are available online at https://www.mdpi.com/1999-4923/12/9/879/s1, SupplementaryInformation.pdf, SupplementaryDDSN.xlsx, DDSN.gephi.

## Abbreviations

The following abbreviations are used in this manuscript:

DDIN	Drug–Drug Interaction Network
DDSN	Drug–Drug Similarity Network
FDA	U.S. Food and Drug Administration
NDA	New Drug Applications
NME	New Molecular Entities
