# Supplementary material for: Uncovering New Drug Properties in Target-Based Drug–Drug Similarity Networks
Source: Pharmaceutics. 2020 Sep 16;12(9):879. doi: 10.3390/pharmaceutics12090879 (PMC7557376; doi:10.3390/pharmaceutics12090879)
Supplement: Supplementary file 1 [file pharmaceutics-12-00879-s001.zip › pharmaceutics-911977 supplementary.pdf]

Supplementary Information: Uncovering new drug properties in target-based drug-drug similarity networks

Lucreția Udrescu[1], Paul Bogdan[2], Aimée Chiș[3], Ioan Ovidiu Sîrbu[3], Alexandru Topîrceanu[4],

Renata-Maria Văruț[5], and Mihai Udrescu[4]*

[1]"Victor Babeș" University of Medicine and Pharmacy Timișoara, Department I - Drug Analysis, Timișoara 300041, Romania
[2]University of Southern California, Ming Hsieh Department of Electrical Engineering, Los Angeles, CA 90089-2563, USA
[3]"Victor Babeș" University of Medicine and Pharmacy Timișoara, Department of Biochemistry and Pharmacology - Biochemistry, Timișoara 300041, Romania
[4]University Politehnica of Timișoara, Department of Computer and Information Technology, Timișoara 300223, Romania
[5]University of Medicine and Pharmacy of Craiova, Faculty of Pharmacy, Craiova 200349, Romania

1. Clustering methods
  1.1 Modularity clustering 2
  1.2 Force-directed clustering 4
2. Clustering consistency 6
3. Molecular docking results for Azelaic acid 7
4. Molecular docking results for Meprobamate 19
5. Molecular docking results interpretation
  5.1 Azelaic acid 33
  5.2 Meprobamate 35
6. The graphical representations of the docked complexes 36
7. Quantum chemical calculation
  7.1 HOMO-LUMO energies for all ligands (*i.e.*, drugs) in this manuscript 41
  7.2 Mulliken population analysis for partial atomic charges for all ligands (*i.e.*, drugs) in this manuscript 46
  7.3 Molecular electrostatic potential surfaces for all ligands (*i.e.*, drugs) in this manuscript 49
8. Docking results validation 52

1. Clustering methods

1.1 Modularity clustering

The modularity $\mathcal{M}$, corresponding to a community detection/clustering of network $G = (V, E)$ that classifies vertices $v_i \in V$ in one of the clusters $C_1, C_2, \dots C_m$ with $\bigcup_{i=1}^{m} C_i = V$, is

$$\mathcal{M}_m = \sum_{C_i \in \mathcal{C}_m} \left(\frac{\left|E_{C_i}\right|}{|E|} - \frac{\frac{1}{2} d_{C_i}^2}{\frac{1}{2} d^2} \right). \qquad (1)$$

The community detection of network G by finds the clustering $\mathcal{C}_m = \{C_1, C_2, \dots C_m\}$ such that the modularity $\mathcal{M}_m$ is maximized (Newman, M.E., 2006. Modularity and community structure in networks. Proceedings of the national academy of sciences, 103(23), pp.8577-8582). The approach is to find the best method of dividing a graph in 2 communities. (Best method means the method that gives the maximum clustering modularity.) Such a method can then be applied recursively on each resulted community, thus trying to divide them further; the entire process comes to an end when the overall modularity cannot be increased further. Therefore, the entire process is based on further splitting a graph/subgraph in two distinct communities/clusters, such that we achieve the maximum possible overall modularity. We rewrite the graph modularity as

$$\mathcal{M} = \frac{1}{4k} \sum_{ij} \left(\mathrm{A_{ij}} - \frac{\mathrm{d_i d_j}}{2\mathrm{k}} \right) \left(\mathrm{s_i s_j} + 1 \right). \qquad (2)$$

In equation 2, A_{ij} is the graph's adjacency matrix, d_i and d_j are respectively the degrees of vertices/nodes v_i and v_j, and k is the total number of edges in the network ($\mathrm{k} = |E| = \frac{1}{2} \sum_i d_i$ for an unweighted network). Also, $s_i = 1$ if v_i is classified in community 1 and $s_i = -1$ if v_i is classified in community 2 [2]. Therefore, we have

$$\frac{1}{2}\left(s_i s_j + 1\right) = \begin{cases} 1 \text{ if } i, j \text{ are in the same community} \\ 0 \text{ otherwise} \end{cases}. \qquad (3)$$

We have $2k = \sum_i d_i = \sum_{ij} A_{ij}$, hence we rewrite equation 2 as

$$\mathcal{M} = \frac{1}{4k} \sum_{ij} \left(A_{ij} - \frac{d_i d_j}{2k} \right) s_i s_j. \qquad (4)$$

Further, we express equation 4 as

$$\mathcal{M} = \frac{1}{4k}\mathbf{s}^{\mathrm{T}}M\mathbf{s}. \tag{5}$$

In equation 5, $\mathbf{s}$ is the column vector containing the s_i elements and $\mathbf{s}^T$ the transpose of $\mathbf{s}$, and $M_{ij} = A_{ij} - \frac{d_i d_j}{2k}$ is called the modularity matrix.

We rewrite $\mathbf{s}$ as a linear combination of normalized eigenvectors $\mathbf{u}_i$ of *M*, $\mathbf{s} = \sum_i a_i \mathbf{u}_i$, with $a_i = \mathbf{u}_i{}^{\boldsymbol{T}}\mathbf{s}$; it then follows that

$$\mathcal{M} = \frac{1}{4k}\sum_i a_i \mathbf{u}_i{}^{\boldsymbol{T}} M \sum_j a_j \mathbf{u}_j = \frac{1}{4k}\sum_i \left(\mathbf{u}_i{}^{\boldsymbol{T}}\mathbf{s}\right)^2 \mu_i. \tag{6}$$

In equation 6, μ_i is the eigenvalue of *M* corresponding to eigenvector $\mathbf{u}_i$. As eigenvectors are orthogonal, max$\{\mathcal{M}\}$ can be achieved by setting $s_i = 1$ if the corresponding elements of $\mathbf{u}_i$ are positive and $s_i = -1$ otherwise, thus dividing the network according to the maximum modularity.

As our DDSN is e weighted network, each edge $e_{ij} \in E$ has a weight w_{ij}. Therefore, equation 1 is rewritten as

$$\mathcal{M}_m = \sum_{C_i \in \mathcal{C}_m} \left(\frac{\mathrm{w}_{E_{C_i}}}{\mathrm{w}_E} - \frac{\frac{1}{2}w_{C_i}^2}{\frac{1}{2}w_V^2} \right). \tag{7}$$

In equation 7, w_E is the total edge weight of edges *E* in *G*, $w_{E_{C_i}}$ is the total edge weight of edges in cluster C_i, w_V is the total edge weight of all vertices *V* in *G*, and w_{C_i} is the total edge weight of vertices in cluster C_i.

1.2 Force-directed clustering

Because each edge is either intra-cluster or inter-cluster, we have

$$\sum_{\mathrm{C_i} \in \mathcal{C}_m} \frac{\mathrm{w}_{\mathrm{E}_{\mathrm{C_i}}}}{\mathrm{w_E}} + \sum_{(\mathrm{C_i},\mathrm{C_j}) \subset \mathcal{C}_m; \mathrm{i} \neq \mathrm{j}} \frac{\mathrm{w}_{\mathrm{E}_{\mathrm{C_i C_j}}}}{\mathrm{w_V}} = 1. \quad (8)$$

In equation 8, we have $w_{E_{C_i C_j}}$ as the total edge weight of the edges between clusters C_i and C_j.

We can also rewrite equation 7 in terms of inter-cluster edge weights, with w_{C_i} representing the total edge weight of vertices in cluster C_i, as follows

$$\sum_{(C_i,C_j) \subset \mathcal{C}_m; i \neq j} \left(-\frac{w_{E_{C_i C_j}}}{w_E} + \frac{\mathrm{w_{C_i} w_{C_j}}}{\frac{1}{2}\mathrm{w_V^2}} \right) = -\sum_{(v_i,v_j) \in \mathrm{V}; C_m(v_i) \neq C_m(v_j)} \left(\frac{\mathrm{w_{ij}}}{\mathrm{w_E}} - \frac{\mathrm{w_i w_j}}{\frac{1}{2}\mathrm{w_V^2}} \right). \quad (9)$$

In equation 9, w_i represents the total weight of edges incident to node/vertex v_i (i.e., the weighted degree of vertex v_i), and $C_m(v_i) \neq C_m(v_j)$ means that vertices v_i and v_j pertain to different clusters within clustering C_m.

Without changing the modularity $\mathcal{M}_m$ of C_m, the *m* clusters can be considered as positions in $\mathbb{R}^{m-1}$, and each pair of clusters has the distance 1, such that we can interpret the *m* clusters as forming a (*m*-1)–simplex with edge length 1. Thus, C_m becomes a (*m*-1)–dimensional layout and its modularity can be expressed as

$$-\sum_{(v_i,v_j) \in \mathrm{V}; i \neq j} \left(\frac{\mathrm{w_{ij}}}{\mathrm{w_E}} |\gamma_\mathrm{i} - \gamma_\mathrm{j}| - \frac{\mathrm{w_i w_j}}{\frac{1}{2}\mathrm{w_V^2}} |\gamma_\mathrm{i} - \gamma_\mathrm{j}| \right). \quad (10)$$

In equation 10, γ_i and γ_j represent the positions of vertices v_i and v_j in $\mathbb{R}^{m-1}$.

Because the distances between the vertices are binary, the modularity of C_m is

$$-\sum_{(v_i,v_j)\in V;i\neq j}\left(\frac{w_{ij}}{w_E}\left|\gamma_i-\gamma_j\right|^{a+1}-\frac{w_i w_j}{\frac{1}{2}w_V^2}\left|\gamma_i-\gamma_j\right|^{r+1}\right) \tag{11}$$

for all $a, r \in \mathbb{R}$, with a > -1 and $r > 1$.

As the force is the negative gradient of energy, we transform the force model into an energy model, so that we can express the equilibria situation as energy minima

$$\sum_{(v_i,v_j);i\neq j}\left(w_{ij}\frac{\left|\gamma_i-\gamma_j\right|^a}{a+1}-w_i w_j\frac{\left|\gamma_i-\gamma_j\right|^r}{r+1}\right) \tag{12}$$

As formally shown by Noack (Noack, A., 2009. Modularity clustering is force-directed layout. Physical Review E, 79(2), p.026102), the energy layout models that minimize energy are maximizing modularity, hence the energy layouts perform modularity clustering. Indeed, energy layouts are more than mere visualization tools. Nonetheless, the energy layouts have the advantage of conveying more information about the positions of the vertices, vicinities, overlapping zones, etc. Conversely, the maximum modularity clustering would have put all vertices/nodes in a cluster in the same position.

2. Clustering consistency

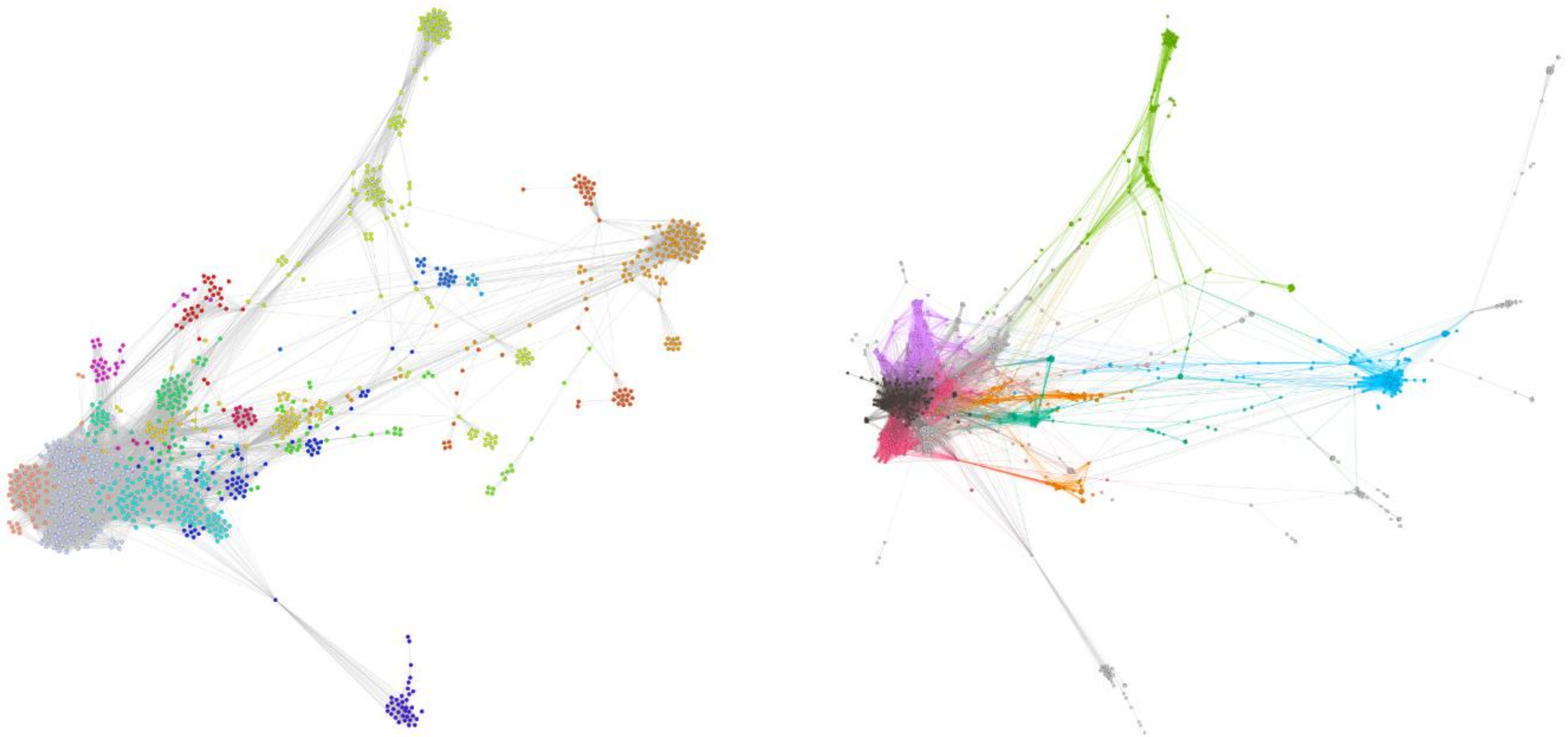

Figure C1. Visual comparison between the drug-drug similarity network (DDSN) built with drug-target data from DrugBank 4.2 (left - it is the DDSN analyzed in our paper) and DrugBank 4.5 (right). Because the 4.5 version has more drugs than 4.2, the structures exhibit some differences—also, 4.5 contains more drug-target interactions, leading to bigger weights, more significant attraction forces, thus tighter topological communities. (Another difference is that we colored all links in grey for the 4.2 DDSN, whereas for the 4.5 DDSN, we assigned the color of the bigger-degree incident node.) Nonetheless, upon visual inspection, the two structures appear very similar.

3. Molecular docking results for Azelaic acid

Table S1. The molecular interactions between Azelaic acid (i.e., the repositioning hint – $d_h^{\text{anticancer}}$), Progesterone and Abiraterone drugs from $\mathscr{D}_6^{\text{anticancer}}$ reference drugs with already accounted anticancer activity), Fosinopril and Furosemide (drugs from $\mathscr{D}_n^{\text{anticancer}}$ reference drugs, with no reported anticancer activity) with Estrogen receptor alpha. The residues shown in bold represent the common Estrogen receptor alpha amino acids involved in the same type of interaction with the tested drugs (Estrogen receptor alpha is a target from $\mathscr{T}_6^{\text{anticancer}}$).

$\mathscr{T}_6^{\text{anticancer}}$ **Estrogen receptor alpha**									
Drug name	Drug role	Lowest free energy of binding [kcal/mol]	Estimated inhibition constant [Temp 298.15 K]	Conventional hydrogen bond	Carbon hydrogen bond	Alkyl interaction	Pi-alkyl interaction	Pi-sigma interaction	Interactive amino acid residues (Van der Waals interaction)
**Azelaic acid**	Repositioning hint (No reported interaction with the target)	-4.48	524.50 µM	ALA$_{A307}$<br>ARG$_{A363}$ (2)<br>ASP$_{A369}$	--	--	--	--	ALA$_{A318}$, ASP$_{A321}$, ALA$_{A322}$, VAL$_{A364}$, PRO$_{A365}$, **GLY$_{A366}$**, VAL$_{A368}$
Progesterone	Reference drug (agonist, inhibitor, downregulator)	-5.89	48.06 µM	VAL$_{A364}$<br>VAL$_{A367}$<br>VAL$_{A368}$	--	ALA$_{A318}$, (2)<br>ARG$_{A363}$ (2)<br>PRO$_{A365}$	--	--	ALA$_{A307}$, LEU$_{A310}$, GLN$_{A314}$, LYS$_{A362}$, **GLY$_{A366}$**, ASP$_{A369}$
Abiraterone	Reference drug (No reported interaction with the target)	-7.11	6.09 µM	LYS$_{A362}$<br>VAL$_{A368}$	--	ALA$_{A307}$<br>LEU$_{A310}$<br>ALA$_{A318}$<br>PRO$_{A365}$	--	--	GLN$_{A314}$, ARG$_{A363}$, **GLY$_{A366}$**, PHE$_{A367}$
Fosinopril	Reference drug (No reported interaction with the target)	-2.03	32.30 mM	ILE$_{A326}$	PRO$_{A325}$	ILE$_{A326}$ (2)<br>TRP$_{A393}$ (2)<br>ARG$_{394}$	ILE$_{A326}$ (2)<br>TRP$_{A393}$ (2)<br>ARG$_{394}$	--	LEU$_{A320}$, GLU$_{A323}$, PRO$_{A324}$, GLY$_{A422}$, PHE$_{A44}$5, VAL$_{A446}$
Furosemide	Reference drug (No reported interaction with the target)	-3.62	2.20 mM	ARG$_{A434}$<br>LEUV$_{509}$	--	ALA$_{A430}$<br>ARG$_{A434}$<br>HIS$_{A513}$	ALA$_{A430}$<br>ARG$_{A434}$<br>HIS$_{A513}$	HIS$_{A513}$	THR$_{431}$, GLN$_{506}$, ILE$_{A510}$,SER$_{A512}$

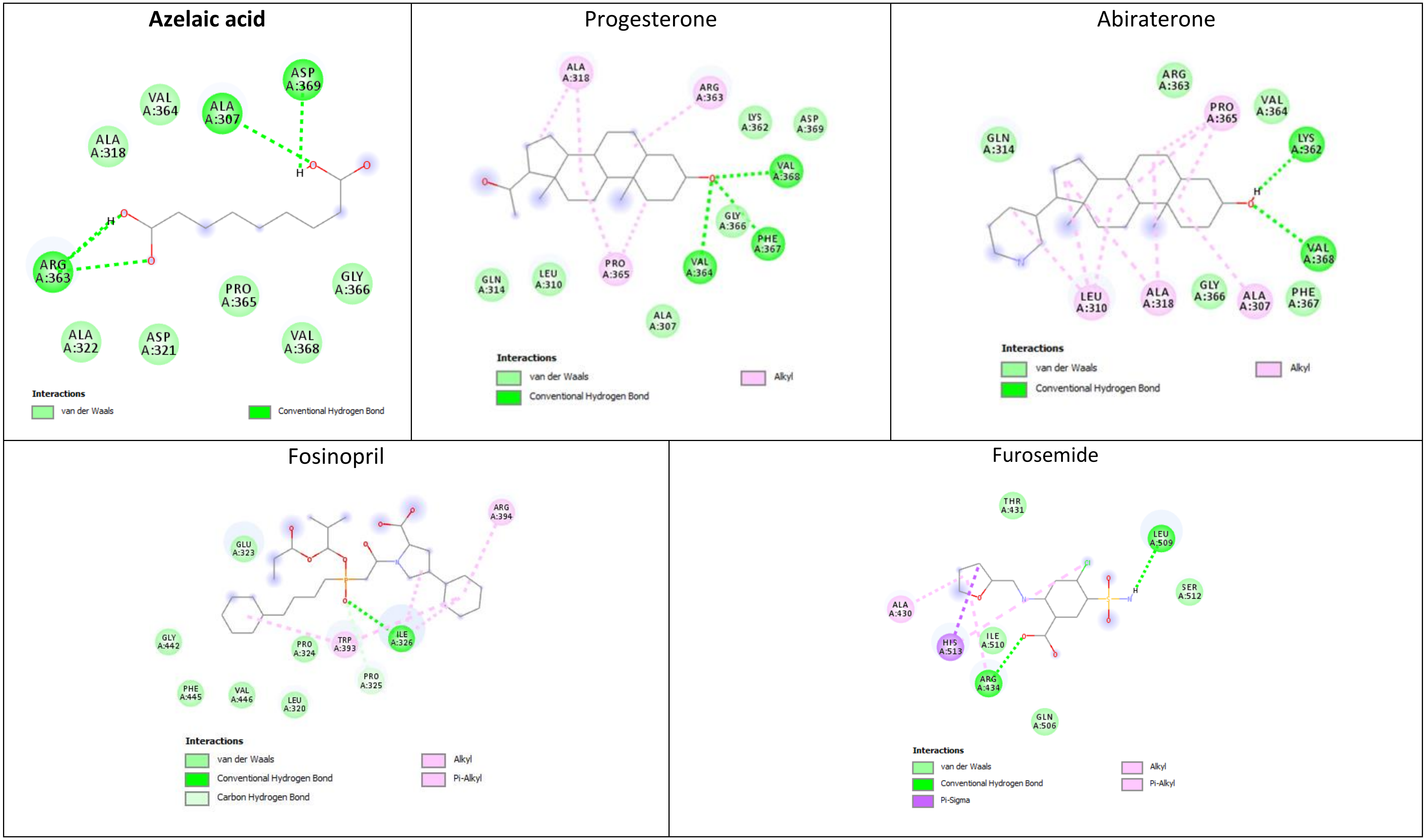

Figure S1. 2D molecular model of interactions between Azelaic acid, Progesterone, Abiraterone, Fosinopril and Furosemide (drugs from $\mathscr{D}_t^{\text{anticancer}}$) and the amino acid residues in Estrogen receptor alpha (a target from $\mathscr{T}_6^{\text{anticancer}}$). The docking software places the 2D chemical representation of the drug molecule in the center of each square. The colored disks represent the amino acids surrounding the drug molecule, while the dotted lines represent the interactions between the target's amino acids and the drug molecule. which is surrounded by the interacting amino acids (represented as colored disks) of the target (shown as dotted-lines); the maps indicates the target's amino acids that establish van der Waals interactions with the drug, but these interactions are not represented as dotted-lines. The diagrams also indicate the amino acids that establish van der Waals interactions with the drug, but these interactions are not represented.

Table S2. The molecular interactions between Azelaic acid (i.e., the repositioning hint – $d_h^{\text{anticancer}}$), Progesterone and Abiraterone (drugs from $\mathscr{D}_6^{\text{anticancer}}$ reference drugs, with already accounted anticancer activity), Fosinopril and Furosemide (drugs from $\mathscr{D}_n^{\text{anticancer}}$ reference drugs, with no reported anticancer activity) with Estrogen receptor beta (a target from $\mathscr{T}_6^{\text{anticancer}}$). The residues shown in bold represent the common Estrogen receptor beta amino acids involved in the same type of interaction with the tested drugs.

$\mathscr{T}_6^{\text{anticancer}}$ **Estrogen receptor beta**									
Drug name	Drug role	Lowest free energy of binding [kcal/mol]	Estimated inhibition constant [Temp 298.15 K]	Conventional hydrogen bond	Carbon hydrogen bond	Alkyl interaction	Pi-Alkyl interaction	Halogen bond	Interactive amino acid residues (Van der Waals interaction)
**Azelaic acid**	Repositioning hint (No reported interaction with the target)	-3.11	5.29 µM	GLU$_{A305}$<br>**ARG$_{A346}$**<br>**GLY$_{A472}$**	--	--	--	--	**MET$_{A295}$**, LEU$_{A298}$, LEU$_{A301}$, ***MET$_{A336}$***, LEU$_{A339}$, MET$_{A340}$, LEU$_{A343}$, PHE$_{A356}$, **ILE$_{A373}$**, **MET$_{A473}$**, HIS$_{A475}$, LEU$_{A476}$
Progesterone	Reference drug (agonist, downregulator)	-8.68	435.53nM	**ARG$_{A346}$**<br>PHE$_{A356}$<br>**GLY$_{A472}$**<br>HIS$_{A475}$<br>LEU$_{A476}$	--	LEU$_{A298}$<br>LEU$_{A301}$<br>ALA$_{A302}$<br>MET$_{A336}$<br>LEU$_{A339}$<br>MET$_{A340}$<br>LEU$_{A343}$<br>ILE$_{A376}$	PHE$_{A356}$ (2)	--	**MET$_{A295}$**, GLU$_{A305}$, **ILE$_{A373}$**, LEU$_{A380}$, **MET$_{A473}$**, MET$_{A479}$
Abiraterone	Reference drug (No reported interaction with the target)	-7.9	1.62 µM	SER$_{A333}$<br>GLU$_{A337}$	--	MET$_{A473}$	TRP$_{A335}$,<br>TYR$_{A488}$	--	GLU$_{A332}$, CYS$_{A334}$, ***MET$_{A336}$***, ARG$_{A466}$, ASN$_{A470}$, LYS$_{A471}$, HIS$_{B467}$
Fosinopril	Reference drug (No reported interaction with the target)	-2.54	13.68 mM	LEU$_{B263}$<br>HIS$_{B428}$	SER$_{B264}$<br>PRO$_{B265}$	VAL$_{B438}$<br>MET$_{A453}$	--	--	MET$_{B261}$, ASP$_{B431}$, ALA$_{B432}$, ASP$_{B435}$, TRP$_{B439}$, GLN$_{A449}$, GLN$_{A450}$
Furosemide	Reference drug (No reported interaction with the target)	-3.51	2.66 mM	PRO$_{A285}$<br>ALA$_{A287}$<br>PHE$_{A289}$	--	--	PHE$_{A289}$	GLU$_{A366}$	SER$_{A283}$, ARG$_{A284}$, PRO$_{A288}$

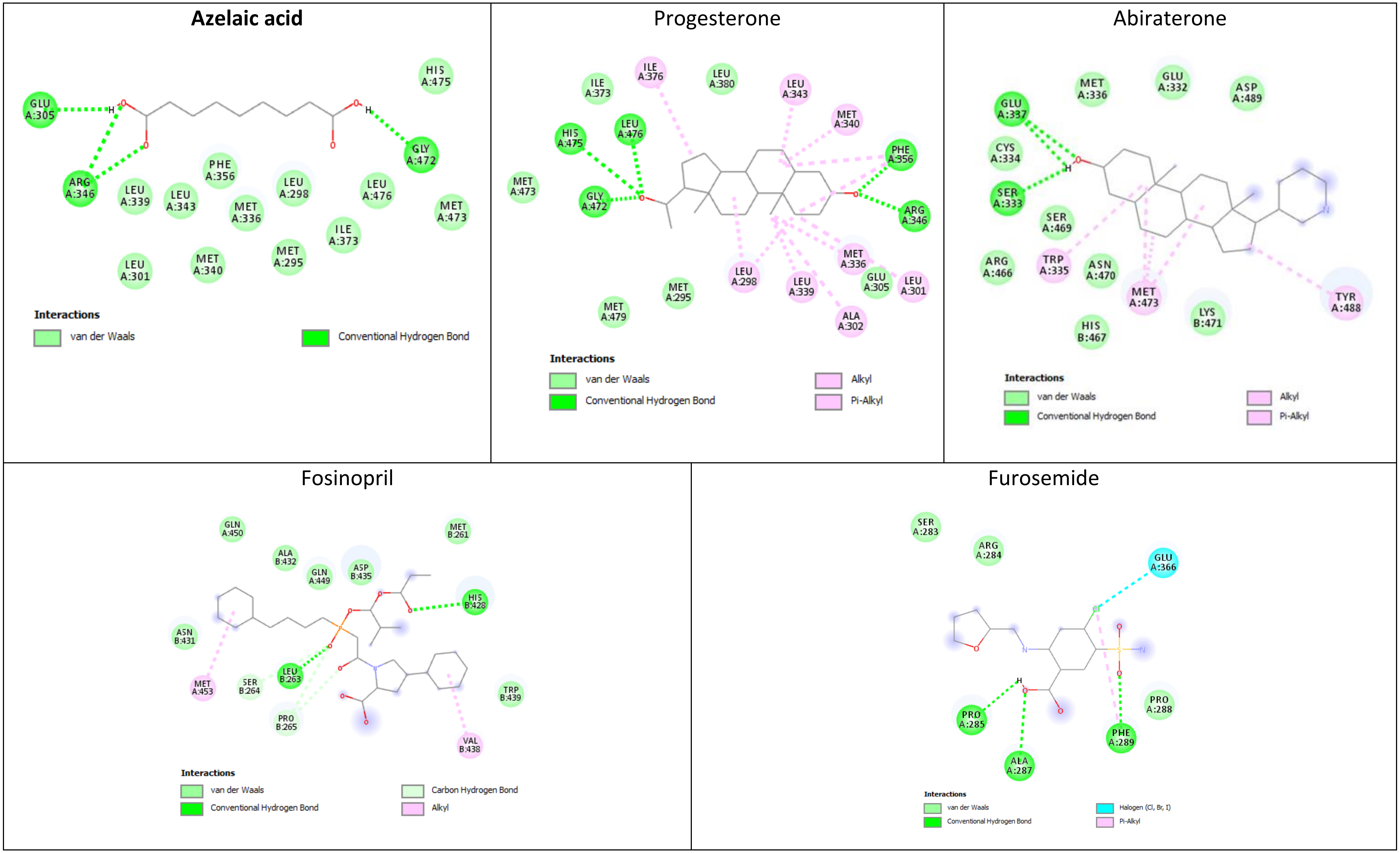

Figure S2. The 2D molecular model generated by the molecular docking simulation, for interactions between Azelaic acid, Progesterone, Abiraterone, Fosinopril, and Furosemide ($d_t^{\text{anticancer}}$ drugs), and the amino acid residues in Estrogen receptor beta (a target from $\mathscr{T}_6^{\text{anticancer}}$). The docking software places the 2D chemical representation of the drug molecule in the center of each square. The colored disks represent the amino acids surrounding the drug molecule, while the dotted lines represent the interactions between the target's amino acids and the drug molecule. The diagrams also indicate the amino acids that establish van der Waals interactions with the drug, but these interactions are not represented.

Table S3. The molecular interactions between Azelaic acid (i.e., the repositioning hint – $d_h^{\text{anticancer}}$), Progesterone and Abiraterone (a drug from $\mathscr{D}_6^{\text{anticancer}}$ reference drug with already accounted anticancer activity), Fosinopril and Furosemide (drugs from $\mathscr{D}_n^{\text{anticancer}}$ reference drugs, with no reported anticancer activity) with Progesterone receptor. The residues shown in bold represent the common Progesterone receptor (a target from $\mathscr{T}_6^{\text{anticancer}}$) amino acids involved in the same type of interaction with the tested drugs.

$\mathscr{T}_6^{\text{anticancer}}$ **Progesterone receptor**								
Drug name	Drug role	Lowest free energy of binding [kcal/mol]	Estimated inhibition constant [Temp 298.15 K]	Conventional hydrogen bond	Carbon hydrogen bond	Alkyl interaction	Pi-Alkyl interaction	Interactive amino acid residues (Van der Waals interaction)
**Azelaic acid**	Repositioning hint (No reported interaction with the target)	-4.54	471.16 µM	**GLN_{B725}**<br>**ARG_{B766}**<br>LEU_{B887}	--	--	--	***LEU_{B721}***, MET_{B756}, MET_{B759}, **VAL_{B760}**, **LEU_{B763}**, **PHE_{B778}**, LEU_{B797}, **MET_{B801}**, HIS_{B888}, TYR_{B890}, **CYS_{B891}**
Progesterone	Reference drug (agonist)	-11.17	6.47 nM	ASP_{B719}<br>**GLN_{B725}**<br>**ARG_{B766}**	--	LEU_{B718}<br>LEU_{B721}<br>MET_{B756}<br>MET_{B759}<br>LEU_{B797}	TYR_{B890}	LEU_{B715}, **VAL_{B760}**, MET_{B722}, TRP_{B755}, **LEU_{B763}**, **PHE_{B778}**, **MET_{B801}**, **CYS_{B891}**, THR_{B894}, VAL_{B903}, PHE_{B905}, MET_{B909}
Abiraterone	Reference drug (No reported interaction with the target)	-11.90	1.89 nM	MET_{B759},<br>***ARG_{B766}***	--	LEU_{B715}<br>LEU_{B718}<br>LEU_{B797}<br>LEU_{B887}<br>CYS_{B891}	PHE_{B778}	ASN_{B719}, ***LEU_{B721}***, GLY_{B722}, TRP_{B755}, THR_{B894}, VAL_{B903}, PHE_{B905}, MET_{B909}
Fosinopril	Reference drug (No reported interaction with the target)	-2.97	6.69 mM	ASP_{B697},<br>LYS_{B731}	SER_{B728}	PRO_{B696}<br>TRP_{B732}	--	SER_{B693}, ILE_{B694}, GLU_{B695}, ILE_{B699}, ARG_{B724}, GLN_{B725}, LEU_{B727}, SER_{B728}, SER_{B735}
Furosemide	Reference drug (No reported interaction with the target)	-4.88	265.75 µM	GLN_{A682} (2)<br>LEU_{A683}<br>ILE_{A684}	--	--	--	ASN_{A689}, MET_{A692}

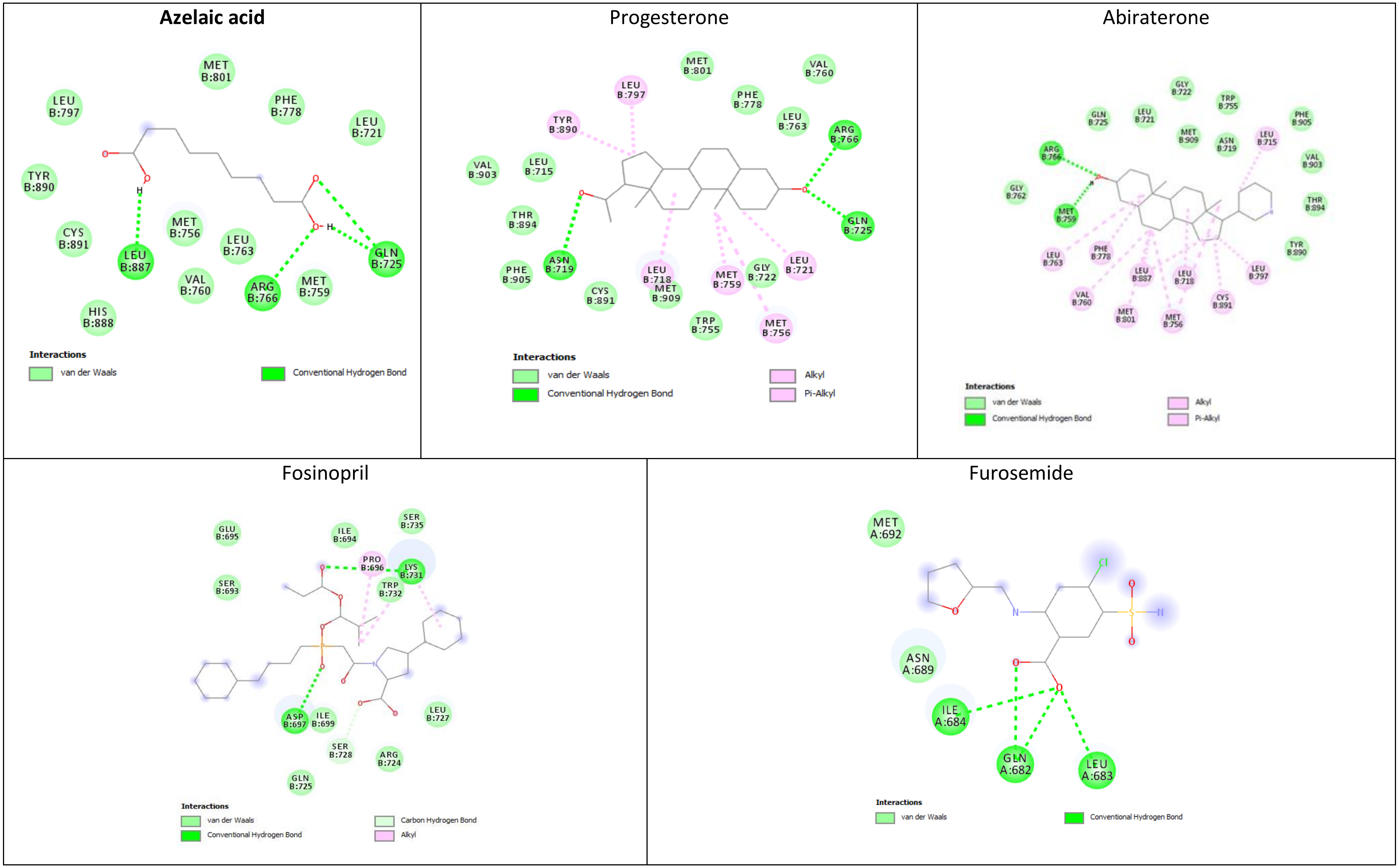

Figure S3. The 2D diagrams generated by the molecular docking simulation, for interactions between Azelaic acid, Progesterone, Abiraterone, Fosinopril, and Furosemide (i.e., $d_t^{\text{anticancer}}$ drugs), and the amino acid residues in Progesterone receptor (a target from $\mathscr{T}_6^{\text{anticancer}}$). The docking software places the 2D chemical representation of the drug molecule in the center of each square. The colored disks represent the amino acids surrounding the drug molecule, while the dotted lines represent the interactions between the target's amino acids and the drug molecule. The diagrams also indicate the amino acids that establish van der Waals interactions with the drug, but these interactions are not represented.

Table S4. The molecular interactions between Azelaic acid (i.e., the repositioning hint – $d_h^{\text{anticancer}}$), Progesterone and Abiraterone ($d_6^{\text{anticancer}}$ drugs from $\mathscr{D}_6^{\text{anticancer}}$ reference drugs with known anticancer activity), Fosinopril and Furosemide (drugs from $\mathscr{D}_n^{\text{anticance}}$ reference drugs, with no reported anticancer activity) with Steroid 17-alpha-hydroxylase/17,20 lyase. The residues shown in bold represent the common Steroid 17-alpha-hydroxylase/17,20 lyase amino acids involved in the same type of interaction with the test and reference drugs (Steroid 17-alpha-hydroxylase/17,20 lyase is a target from $\mathscr{T}_6^{\text{anticancer}}$).

$\mathscr{T}_6^{\text{anticancer}}$ **Steroid 17-alpha-hydroxylase/17,20 lyase**									
Drug name	Drug role	Lowest free energy of binding [kcal/mol]	Estimated inhibition constant [Temp 298.15 K]	Covalent bond	Conventional hydrogen bond	Carbon hydrogen bond	Alkyl interaction	Pi-Alkyl interaction	Interactive amino acid residues (Van der Waals interaction)
**Azelaic acid**	Reference drug (No reported interaction with the target)	**-8.49**	**600.71 nM**	--	ARG_{B96}, ILE_{B112}, TRP_{B121},ARG_{B125}, **ILE_{B371}**, **HIS_{B373}**, ARG_{B440}	--	--	--	***ALA_{B113}***, **LEU_{B370}**, **SER_{B441}**, CYS_{B442}, **GLY_{B436}**
Progesterone	Reference drug (substrate, inhibitor)	-8.72	406.22 nM	--	**ILE_{B371}**, **HIS_{B373}**	--	VAL_{B366} (3) ALA_{B367} CYS_{B442} (2) HEM	PHE_{B435}	LEU_{B86}, ARG_{B96}, THR_{B306}, LEU_{B361}, **LEU_{B370}**, PRO_{B434}, **GLY_{B436}**, ALA_{B437}, ARG_{B440}, **SER_{B441}**, ALA_{B448}
Abiraterone	Reference drug (inhibitor)	-8.99	402.33nM	HEM	ASN_{202}	***ALA_{B113}***, ***GLY_{B436}***	ILE_{B205} ILE_{B206} LEU_{B209} ALA_{B367} ***LEU_{B370}*** VAL_{B482}	PHE_{B114}	**ALA_{B113}**, ARG_{B239}, GLY_{B301}, PRO_{B434}, **GLY_{B436}**
Fosinopril	Reference drug (No reported interaction with the target)	-3.86	1.47 mM	--	ASN_{A51}, ARG_{A364}	--	HIS_{A48}, LEU_{A476}	HIS_{A48}	GLY_{A47}, HIS_{A50}, LYS_{A55}, ASP_{C241}, LYS_{C245}, PHE_{A317}, LEU_{A363}, TRP_{A397}, HIS_{A401}, ASP_{A410}, GLN_{A411}, PHEA412, GLU_{A477}, PHE_{A484}
Furosemide	Reference drug (No reported interaction with the target)	-5.64	74.01 µM	--	LYS_{C55} LYS_{C59}	LEU_{C56}	PHE_{C42} ARG_{C45}	PHE_{C42} ARG_{C45}	GLY_{C47}, ASN_{C52}, LEU_{C56}, LYS_{C58}

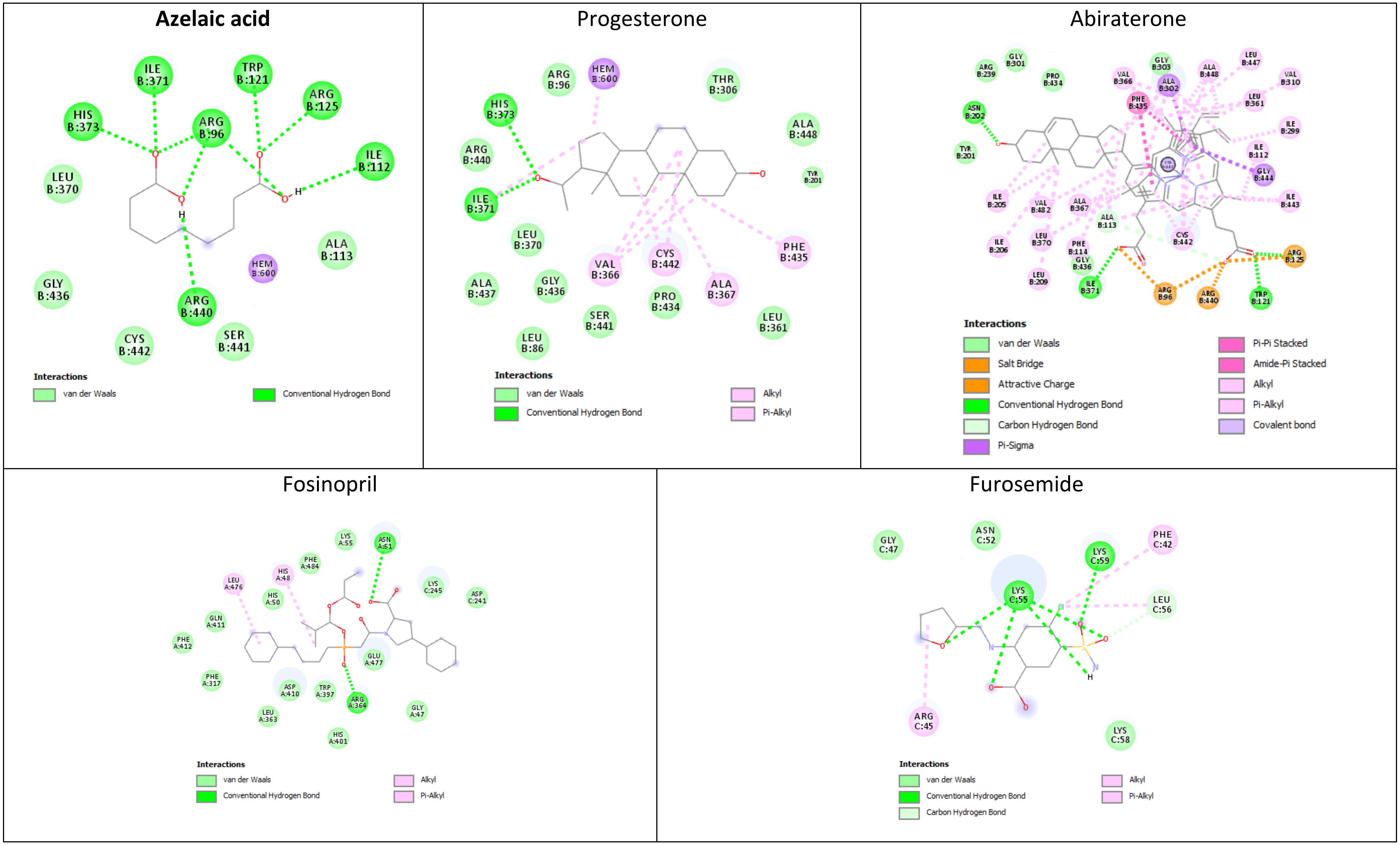

Figure S4. Structure views of the 3D-complexes between Azelaic acid (i.e., the repositioning hint – $d_h^{\text{anticancer}}$), Progesterone and Abiraterone ($d_6^{\text{anticancer}}$ drugs from $\mathscr{D}_6^{\text{anticancer}}$ reference drug with known anticancer activity), Fosinopril and Furosemide (reference drugs from $\mathscr{D}_n^{\text{anticancer}}$, with no reported anticancer activity) with Steroid 17-alpha-hydroxylase/17,20 lyase, which is a target from $\mathscr{T}_6^{\text{anticancer}}$. The docking software places the 2D chemical representation of the drug molecule in the center of each square. The colored disks represent the amino acids surrounding the drug molecule, while the dotted lines represent the interactions between the target's amino acids and the drug molecule. The diagrams also indicate the amino acids that establish van der Waals interactions with the drug, but these interactions are not represented.

Table S5. The molecular interactions between Azelaic acid (i.e., the repositioning hint – $d_h^{\text{anticancer}}$), Progesterone and Abiraterone ($d_6^{\text{anticancer}}$ drugs from $\mathscr{D}_6^{\text{anticancer}}$ reference drugs with known anticancer activity), Fosinopril and Furosemide (drugs from $\mathscr{D}_n^{\text{anticancer}}$ reference drugs, with no reported anticancer activity) with Androgen receptor (a target from $\mathscr{T}_6^{\text{anticancer}}$). The residues shown in bold represent the common target's amino acids involved in the same type of interaction with the test and reference drugs.

$\mathscr{T}_6^{\text{anticancer}}$ **Androgen receptor**								
Drug name	Drug role	Lowest free energy of binding [kcal/mol]	Estimated inhibition constant [Temp 298.15 K]	Conventional hydrogen bond	Pi-donor hydrogen bond	Alkyl interaction	Pi-Alkyl interaction	Interactive amino acid residues (Van der Waals interaction)
**Azelaic acid**	Repositioning hint (No reported interaction with the target)	-5.01	213.54 µM	LEU_{A704}<br>**MET_{A745}**<br>**ARG_{A752}(2)**<br>PHE_{A764}	LEU_{A707}	--	--	LEU_{A707}, **GLY_{A708}, GLN_{A711}**, MET_{A742}, **VAL_{A746}**, ALA_{A748}, **MET_{A749}**, LEU_{A873}
Progesterone	Reference drug (agonist, potentiator)	-10.28	29.31 nM	**MET_{A745}**<br>**ARG_{A752}**	--	LEU_{A704} (2), LEU_{A707}, MET_{A742}, MET_{A745}, MET_{A780}, LEU_{A873}, THR_{877}	--	LEU_{A701}, ASN_{A705}, **GLY_{A708}, GLN_{A711}**, TRP_{A741}, **VAL_{A746}, MET_{A749}**, PHE_{A764}, PHE_{A876}, THR_{A877}, LEU_{A880}, PHE_{A891}
Abiraterone	Reference drug (No reported interaction with the target)	-8.56	527.57 nM	THR_{A755}<br>LYS_{A808}	--	PRO_{A682}, VAL_{A684}, VAL_{A685}, ALA_{A748}, ARG_{A752}	--	GLY_{A683}, **GLN_{A711}**, HIS_{A714}, VAL_{A715}, LEU_{A744}, TRP_{A751}, PHE_{A804}
Fosinopril	Reference drug (No reported interaction with the target)	--2.91	7.41 mM	--	--	LYS_{A912}, PRO_{A913}	--	PRO_{A868}, ARG_{A871}, ILE_{A906}, GLY_{A909}, VAL_{A911}, ILE_{A914}, HIS_{A917}, THR_{A918}
Furosemide	Reference drug (No reported interaction with the target)	-4.13	938.39 µM	GLU_{A793} (2)<br>LYS_{A861}	--	LEU_{A862}	TYR_{A915}	TRP_{A796}, LEU_{A797}, ASP_{A864}, SER_{A865}, PRO_{A868}, HIS_{A917}, THR_{A918}

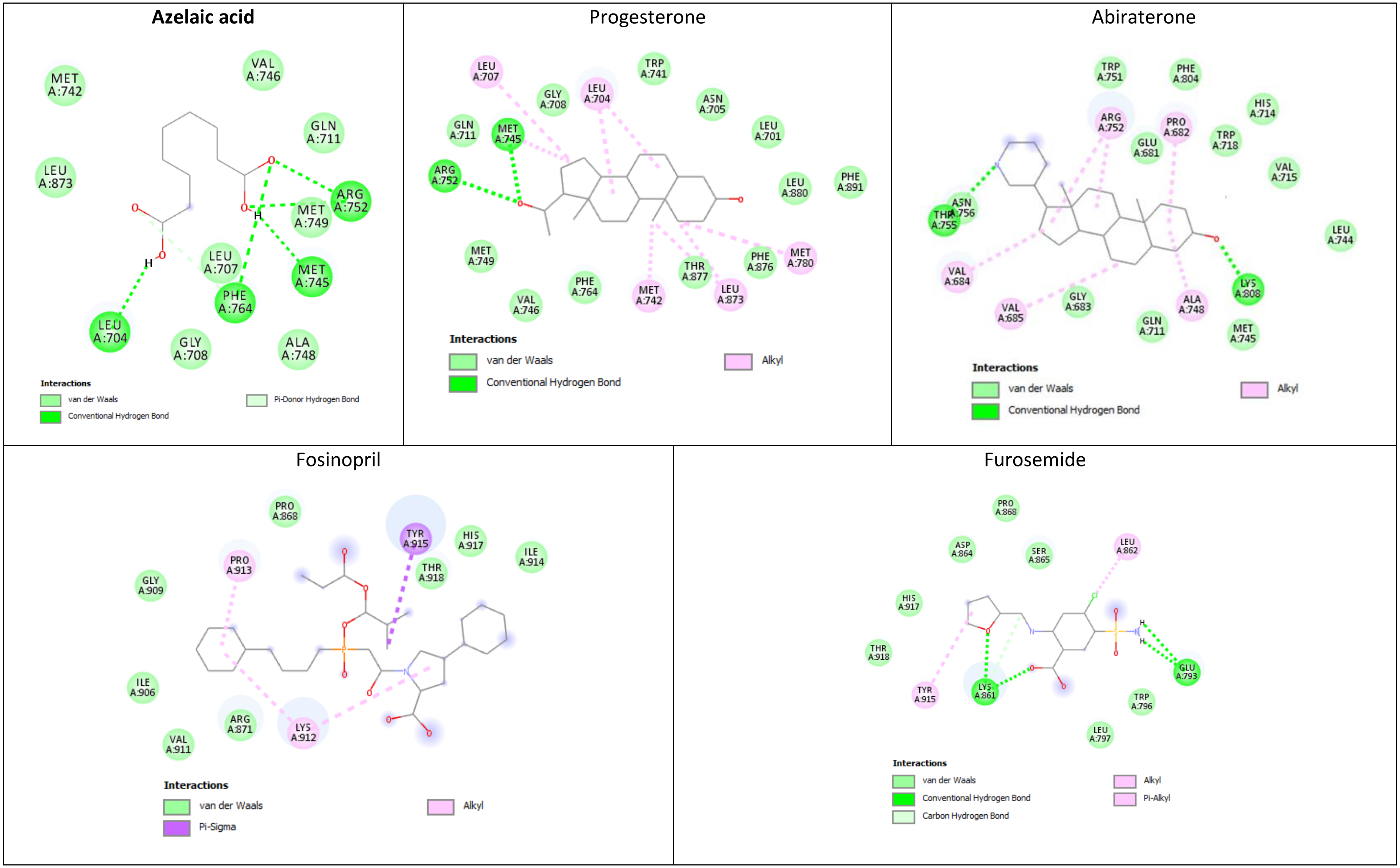

Figure S5. The 2D diagrams generated by the molecular docking simulation for interactions between Azelaic acid, Progesterone, Abiraterone, Fosinopril, and Furosemide (i.e., $d_t^{\text{anticancer}}$ drugs), and the amino acid residues in Androgen receptor (a target from $\mathscr{T}_6^{\text{anticancer}}$). The 2D chemical representation of the drug molecule is in the center of each square. The colored disks represent the amino acids surrounding the drug molecule, while the dotted lines represent the interactions between the target's amino acids and the drug molecule. The diagrams also indicate the amino acids that establish van der Waals interactions with the drug, but these interactions are not represented.

Table S6. The molecular interactions between Azelaic acid (i.e., the repositioning hint – $d_h^{\text{anticancer}}$), Progesterone and Abiraterone ($d_6^{\text{anticancer}}$ drugs from $\mathscr{D}_6^{\text{anticancer}}$ reference drugs with known anticancer activity), Fosinopril and Furosemide (drugs from $\mathscr{D}_n^{\text{anticancer}}$ reference drugs, with no reported anticancer activity) with Mineralocorticoid receptor. The residues shown in bold represent the common Mineralocorticoid receptor amino acids involved in the same type of interaction with the test and reference drugs (Mineralocorticoid receptor is a target from $\mathscr{T}_6^{\text{anticancer}}$).

$\mathscr{T}_6^{\text{anticancer}}$ **Mineralocorticoid receptor**								
Drug name	Drug role	Lowest free energy of binding [kcal/mol]	Estimated inhibition constant [Temp 298.15 K]	Conventional hydrogen bond	Carbon hydrogen bond	Alkyl interaction	Pi-Alkyl interaction	Interactive amino acid residues (Van der Waals interaction)
**Azelaic acid**	Repositioning hint (No reported interaction with the target)	-4.49	507.15 µM	ASN_{D770} GLN_{D776} $\mathbf{ARG_{D817}}$	--	--	--	LEU_{D769}, LEU_{D772}, ALA_{D773}, $\mathbf{TRP_{D806}}$, MET_{D807}, LEU_{D810}, ALA_{D813}, $\mathbf{LEU_{D814}}$, PHE_{D829}, CYS_{D942}, $\mathbf{PHE_{D956}}$, LEU_{D960}
Progesterone	Reference drug (antagonist, agonist)	-10.77	12.72 nM	$\mathbf{ARG_{D817}}$	CYS_{D942}	LEU_{D769} LEU_{D772} ALA_{D773} MET_{D807} LEU_{D810} LEU_{D938} CYS_{D942} MET_{D845}	PHE_{D829}, PHE_{D941}	ASN_{D770}, LEU_{D766}, GLN_{D776}, $\mathbf{TRP_{D806}}$, SER_{D811}, $\mathbf{LEU_{D814}}$, THR_{D945}, VAL_{D954}, $\mathbf{PHE_{D956}}$
Abiraterone	Reference drug (No reported interaction with the target)	-8.08	1.19 µM	GLN_{F776}	--	PRO_{F747}, VAL_{F750}, ALA_{F813}, ARG_{F817}, LYS_{F820}, HIS_{F821}	HIS_{F821}	GLU_{F746}, GLU_{F748}, ILE_{F749}, GLN_{F779}, VAL_{F780}, TRP_{F816}, PHE_{F866}
Fosinopril	Reference drug (No reported interaction with the target)	-2.28	21.22 mM	LYS_{F901} LYS_{F905}	--	PRO_{F738} TYR_{F899}	PRO_{F738} TYR_{F899}	PRO_{F788}, ASN_{F898}, GLU_{F902}, ARG_{F904}
Furosemide	Reference drug (No reported interaction with the target)	-4.01	1.16 mM	ASP_{D884} LYS_{D977}	--	LYS_{D977}	--	LYS_{D883}, GLY_{D974}, PRO_{D978}

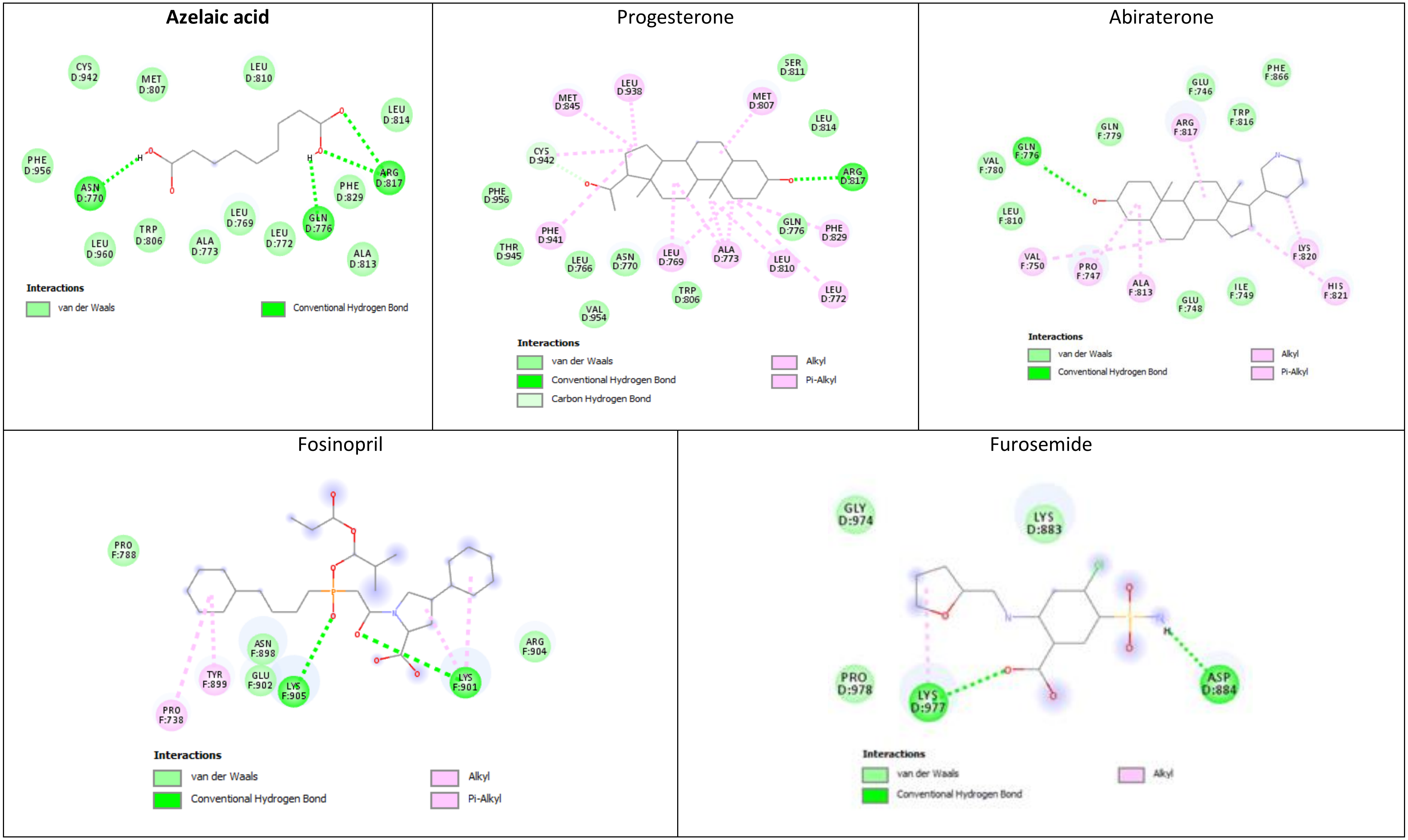

Figure S6. The 2D maps generated by the molecular docking simulation for interactions between Azelaic acid, Progesterone, Abiraterone, Fosinopril, and Furosemide (i.e., $d_t^{\text{anticancer}}$ drugs), and the amino acid residues in Mineralocorticoid receptor (a target from $\mathscr{T}_6^{\text{anticancer}}$). The 2D chemical representation of the drug molecule is in the center of each square. The colored disks represent the amino acids surrounding the drug molecule, while the dashed lines represent the interactions between the target's amino acids and the drug molecule. The diagrams also indicate the amino acids that establish van der Waals interactions with the drug, but these interactions are not represented.

4. Molecular docking results for Meprobamate

Table S7. A comparison of the molecular interactions between Meprobamate (i.e., the repositioning hint $-d_h^{\text{antifungal}}$), Clotrimazole (this drug $\in \mathscr{D}_{25}^{\text{antifungal}}$ reference drugs with documented antifungal activity), Fosinopril and Furosemide (reference drugs from $\mathscr{D}_n^{\text{antifungal}}$, with no reported antifungal activity) and Lanosterol 14-alpha demethylase (Lanosterol 14-alpha demethylase $\in \mathscr{T}_{25}^{\text{antifungal}}$). The residues shown in bold represent the Lanosterol 14-alpha demethylase amino acids involved in the same type of interaction with the tested and reference drugs.

$\mathscr{T}_{25}^{\text{antifungal}}$ **Lanosterol 14-alpha demethylase**								
Drug name	Drug role	Lowest free energy of binding [kcal/mol]	Estimated inhibition constant [Temp 298.15 K]	Conventional hydrogen bond	Carbon hydrogen bond	Alkyl interaction	Pi-alkyl interaction	Interactive amino acid residues (Van der Waals interaction)
**Meprobamate**	**Repositioning hint (No reported interaction with the target)**	-2.77	9.38 mM	**MET_{A358}**<br>MET_{A360}<br>MET_{A460}	--	**PRO_{A210}**	--	VAL_{A102}, **TYR_{A103}**, **PHE_{A105}**, **VAL_{A213}**, **LEU_{A356}**, LEU_{A357}, LEU_{A359}, **VAL_{A461}**
Clotrimazole	Reference drug (Known antagonist, inhibitor)	-7.15	0.0058 mM	**MET_{A358}**	MET_{A460}	**PRO_{A210}**<br>LEU_{A357}<br>MET_{A358}	PHE_{A48}<br>PHE_{A214}	GLY_{A49}, ILE_{A72}, **TYR_{A103}**, **PHE_{A105}**, **VAL_{A213}**, PRO_{A355}, **LEU_{A356}**, MET_{A360}, TYR_{A457}, HIS_{A458}, THR_{A459}, **VAL_{A461}**, VAL_{A462}
Fosinopril	Reference drug (No reported interaction with the target)	-4.85	0.278 mM	--	--	ALA_{A131}<br>LYS_{A426}<br>ILE_{A423}	--	ARG_{A124}, LEU_{A127}, ASN_{A128}, GLU_{A132}, LEU_{A134}, THR_{A135}, ILE_{A136}, PHE_{A139}, GLY_{A418}, VAL_{A419}, HIS_{A420}, LYS_{A421}, CYS_{A422}, GLY_{A424}, GLN_{A425}, PHE_{A427}
Furosemide	Reference drug (No reported interaction with the target)	-4.78	0.315 mM	PRO_{A83}<br>HIS_{A84} (2)<br>HIS_{A86}<br>SER_{A87}<br>GLU_{A409} (2)	GLY_{A410}	--	--	GLU_{A85}, ARG_{A88}, LEU_{A91}, VAL_{A408}, ALA_{A411}

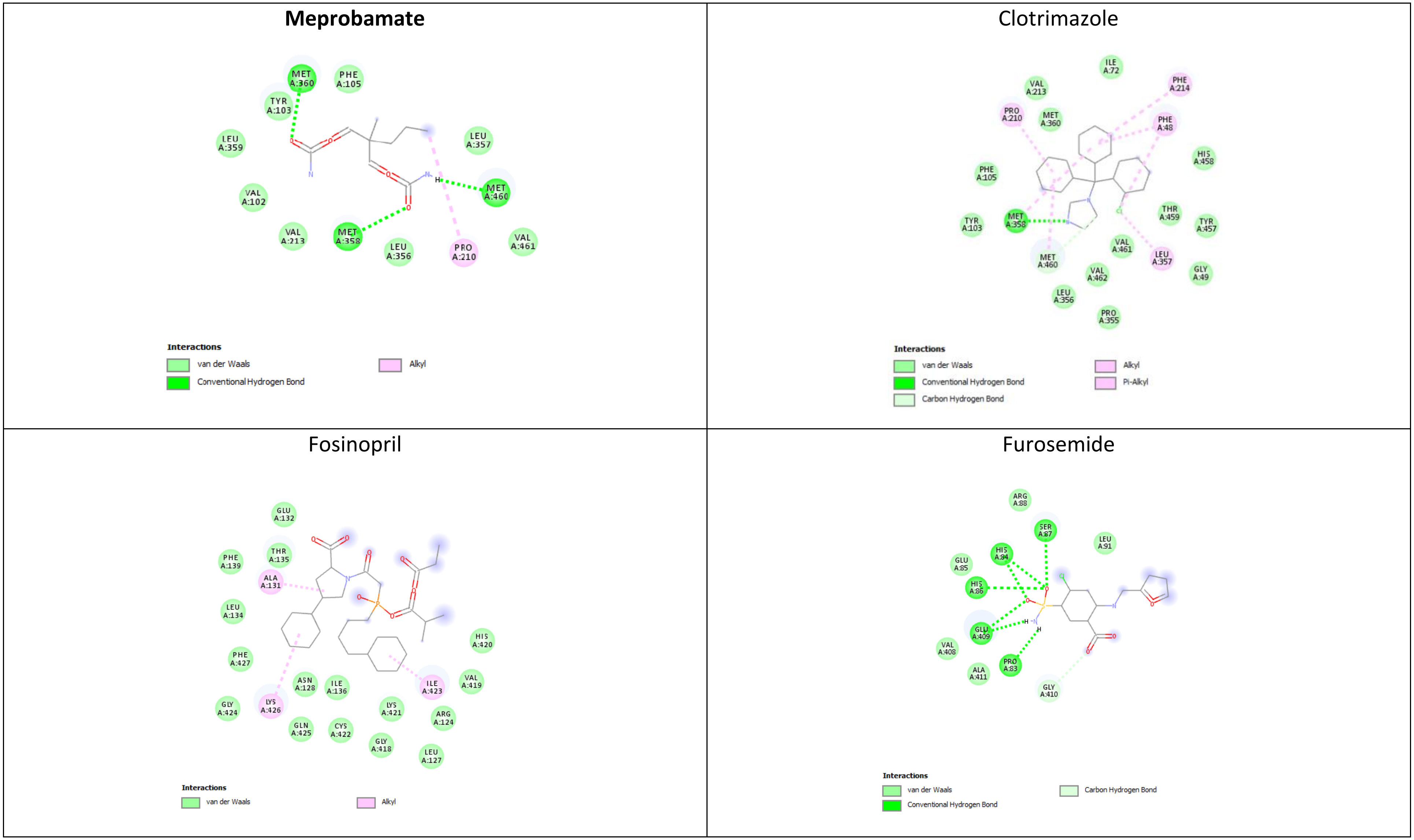

Figure S7. 2D molecular model of interactions between Meprobamate, Fosinopril, Furosemide (tested drugs that $\in \mathcal{D}_t^{antifungal}$), and Clotrimazole (a reference drug from $\mathcal{D}_{25}^{antifungal}$) with the amino acid residues in Lanosterol 14-alpha demethylase (a target $\in \mathcal{T}_{25}^{antifungal}$). The docking software places the 2D chemical representation of the drug molecule in the center of each square. The colored disks represent the amino acids surrounding the drug molecule, while the dotted lines represent the interactions between the target's amino acids and the drug molecule. The maps also indicate the target's amino acids that establish van der Waals interactions with the drug; however, for simplification, this figure does not represent these interactions.

Table S8. The comparison of the molecular interactions between Meprobamate (i.e., the repositioning hint – $d_h^{antifungal}$), Oxiconazole (this drug $\in \mathscr{D}_{25}^{antifungal}$ reference drugs, with already documented antifungal activity), Fosinopril and Furosemide (reference drugs from $\mathscr{D}_n^{antifungal}$, with no reported antifungal activity) and Lanosterol synthase (this target $\in \mathscr{T}_{25}^{antifungal}$). The residues shown in bold represent the Lanosterol synthase amino acids involved in the same type of interaction with the tested and reference drugs.

$\mathscr{T}_{25}^{antifungal}$ **Lanosterol synthase**								
Drug name	Drug role	Lowest free energy of binding [kcal/mol]	Estimated inhibition constant [Temp 298.15 K]	Conventional hydrogen bond	Carbon hydrogen bond	Alkyl interaction	Pi-Alkyl interaction	Interactive amino acid residues (Van der Waals interaction)
**Meprobamate**	Repositioning hint (No reported interaction with the target)	-3.23	4.26 mM	THR$_{A210}$<br>LEU$_{A211}$<br>TRP$_{A216}$<br>SER$_{A241}$ (2)	--	**MET$_{A215}$**<br>**ALA$_{A224}$**<br>**LEU$_{A515}$**	--	**PHE$_{A212}$**, LEU$_{A229}$, CYS$_{A233}$, **TYR$_{A237}$**, TYR$_{A297}$, **LEU$_{A300}$**, PRO$_{A517}$, **MET$_{A525}$**
Oxiconazole	Reference drug (inhibitor)	-6.24	0.0267 mM	MET$_{A215}$	--	**MET$_{A215}$** (3)<br>**ALA$_{A224}$**<br>LEU$_{A229}$<br>LEU$_{A292}$<br>VAL$_{A296}$<br>**LEU$_{A515}$**	TRP$_{A216}$	LEU$_{A211}$, **PHE$_{A212}$**, PRO$_{A213}$, ALA$_{A222}$, PRO$_{A223}$, PRO$_{A226}$, **TYR$_{A237}$**, SER$_{A241}$, LEU$_{A299}$, **LEU$_{A300}$**, LEU$_{A512}$, **MET$_{A525}$**
Fosinopril	Reference drug (No reported interaction with the target)	-5.04	0.2018 mM	GLY$_{A86}$	THR$_{A49}$<br>GLN$_{A88}$	LEU$_{A27}$<br>CYS$_{A29}$<br>LEU$_{A51}$<br>ALA$_{A89}$	PHE$_{A64}$	ASN$_{A28}$, ARG$_{A46}$, GLY$_{A50}$, GLU$_{A52}$, TYR$_{A63}$, VAL$_{A85}$, LEU$_{A87}$, GLU$_{A90}$, ASP$_{A91}$, GLY$_{A92}$, THR$_{A95}$, GLU$_{A406}$, PHE$_{A407}$, SER$_{A409}$, CYS$_{A410}$, LYS$_{A413}$
Furosemide	Reference drug (No reported interaction with the target)	-5.61	0.0773 mM	TYR$_{A98}$<br>GLY$_{A380}$	TYR$_{A704}$	--	TRP$_{A230}$<br>HIS$_{A232}$<br>PHE$_{A696}$	PRO$_{A101}$, PHE$_{A103}$, TRP$_{A192}$, GLY$_{A336}$, PRO$_{A337}$, ILE$_{A338}$, SER$_{A339}$, THR$_{A381}$, VAL$_{A453}$, THR$_{A502}$, TYR$_{A503}$, PHE$_{A521}$, TRP$_{A581}$, VAL$_{A695}$, ASN$_{A697}$, ILE$_{A702}$

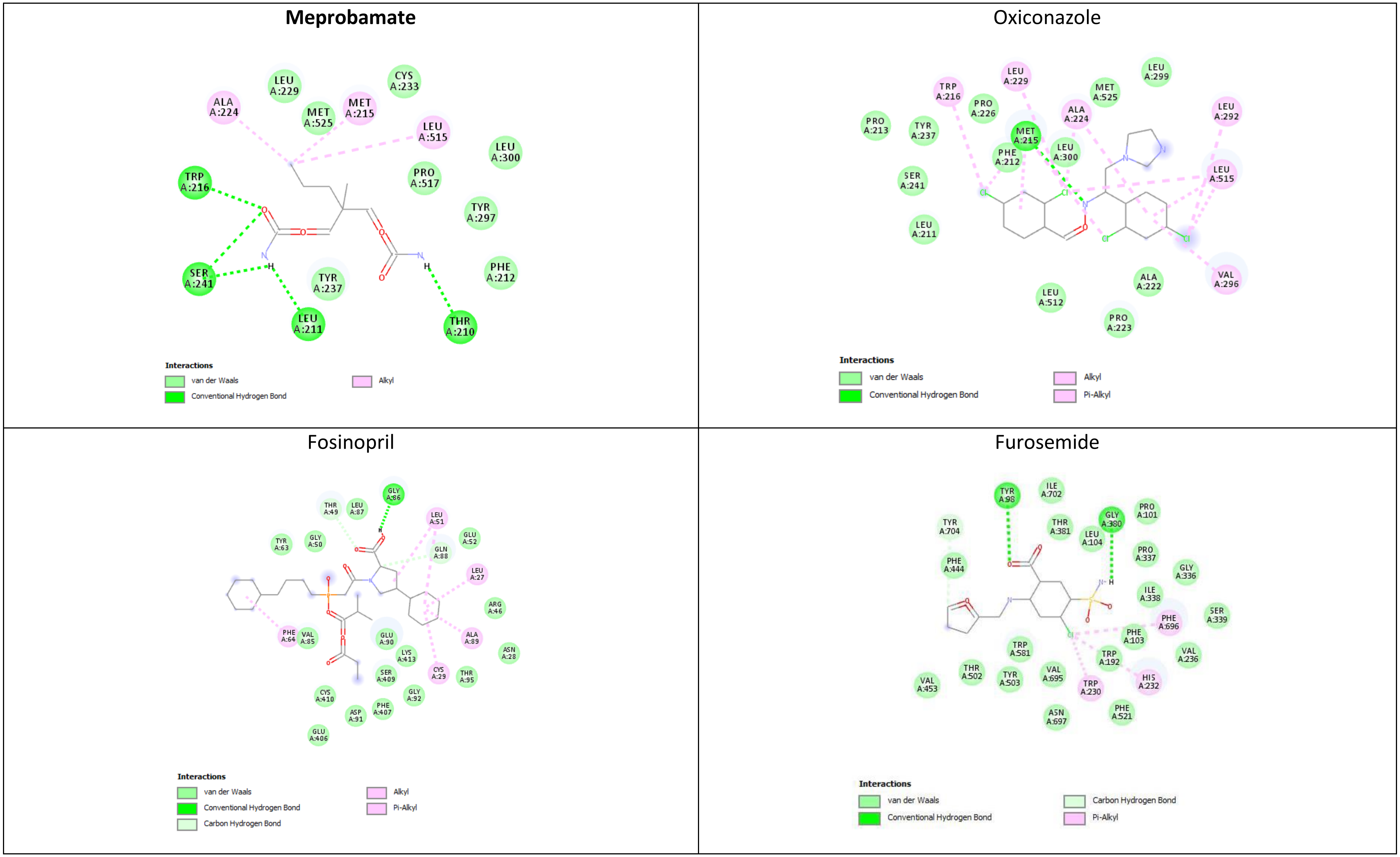

Figure S8. The 2D maps generated by the molecular docking simulation, for interactions between Meprobamate, Fosinopril, and Furosemide (tested drugs from $\mathscr{D}_t^{\text{antifungal}}$ drugs), Oxiconazole (the reference drug $\in \mathscr{D}_{25}^{\text{antifungal}}$) and the amino acid residues in Lanosterol synthase (the target $\in \mathscr{T}_{25}^{\text{antifungal}}$). The docking software places the 2D chemical representation of the drug molecule in the center of each square. The colored disks represent the amino acids surrounding the drug molecule, while the dotted lines represent the interactions between the target's amino acids and the drug molecule. The diagrams also indicate the amino acids that establish van der Waals interactions with the drug; however, for the sake of clarity, these interactions are not represented.

Table S9. A comparison of the molecular interactions between Meprobamate (i.e., the repositioning hint – $d_h^{antifungal}$), Clotrimazole (this drug ∈ $\mathscr{D}_{25}^{antifungal}$ reference drug with already documented antifungal activity), Fosinopril and Furosemide (these drugs ∈ $\mathscr{D}_n^{antifungal}$ reference drugs, with no reported antifungal activity) with Intermediate conductance calcium-activated potassium channel protein 4. The residues shown in bold represent the Intermediate conductance calcium-activated potassium channel protein 4 (this target ∈ $\mathscr{T}_{25}^{antifungal}$) amino acids involved in the same type of interaction with the reference and tested drugs.

$\mathscr{T}_{25}^{antifungal}$	**Intermediate conductance calcium-activated potassium channel protein 4**								
Drug name	Drug role	Lowest free energy of binding [kcal/mol]	Estimated inhibition constant [Temp 298.15 K]	Conventional hydrogen bond	Carbon hydrogen bond	Alkyl interaction	Halogen interaction	Pi-Sulfur interaction	Interactive amino acid residues (Van der Waals interaction)
**Meprobamate**	**Repositioning hint (No reported interaction with the target)**	-1.02	179.63 mM	ALA_{A405} (2)<br>GLU_{A408}<br>THR_{A412}<br>THR_{B398}	THR_{A412}	LEU_{A409}<br>ALA_{A413}	--	--	GLN_{B395}, LYS_{B402}
Clotrimazole	Reference drug (Known antagonist, inhibitor)	-3.60	2.30 mM	--	--	LYS_{A402}<br>ALA_{A405}	GLU_{A408}	--	ASP_{A404}, THR_{A407}<br>ASP_{B397}, THR_{B398}, GLY_{B401}, ASP_{B404}
Fosinopril	Reference drug, randomly chosen (No reported interaction with the target)	-1.96	36.76 mM	--	--	LEU_{B381}	--	--	ILE_{B377}, LEU_{B378}, ASP_{B380}, ASN_{B384}, LEU_{B385}
Furosemide	Reference drug, randomly chosen (No reported interaction with the target)	-2.45	16.05 mM	HIS_{A389}	SER_{A386}	--	--	HIS_{A389}	LEU_{A378}, LEU_{A381}, GLN_{A382}, LEU_{A385}, TYR_{A379}

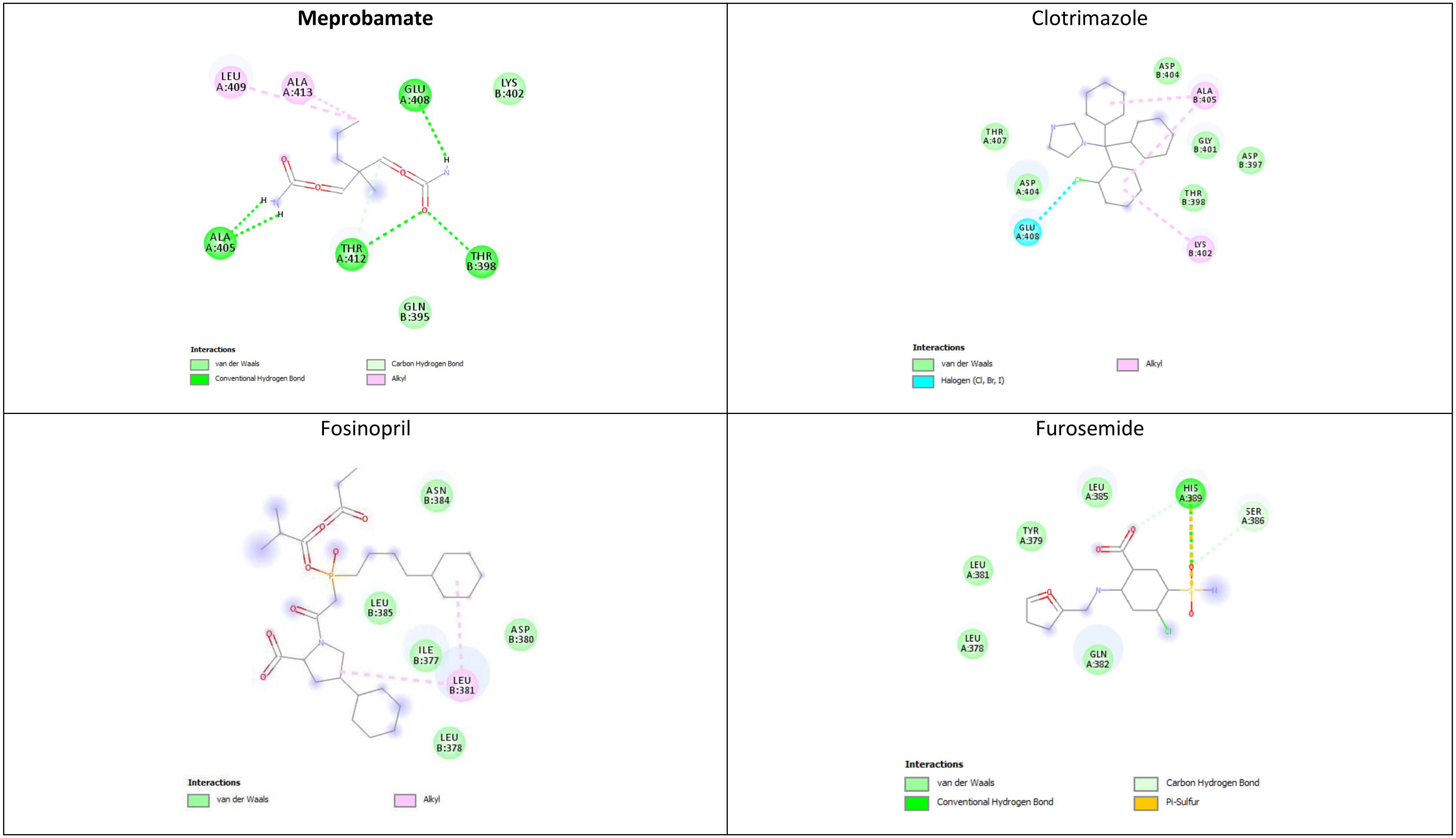

Figure S9. The 2D diagrams generated by the molecular docking simulation, for interactions between Meprobamate (the $d_h^{\text{antifungal}}$ repositioning hint), Fosinopril, and Furosemide (these reference drugs $\in \mathscr{D}_n^{\text{antifungal}}$), and Clotrimazole (the reference drug $\in \mathscr{D}_{25}^{\text{antifungal}}$) with the amino acid residues in Intermediate conductance calcium-activated potassium channel protein 4 (this target $\in \mathscr{T}_{25}^{\text{antifungal}}$). The docking software places the 2D chemical representation of the drug molecule in the center of each square. The colored disks represent the amino acids surrounding the drug molecule, while the dotted lines represent the interactions between the target's amino acids and the drug molecule. The diagrams also indicate the amino acids that establish van der Waals interactions with the drug; however, for the sake of clarity, these interactions are not represented.

Table S10. A comparison of the molecular interactions between Meprobamate (i.e., the repositioning hint – $d_h^{\text{antifungal}}$), Naftifine and Tolnaftate (these drugs $\in \mathscr{D}_{25}^{\text{antifungal}}$ reference drugs with already well-documented antifungal activity), Fosinopril and Furosemide (these drugs $\in \mathscr{D}_n^{\text{antifungal}}$ reference drugs, with no reported antifungal activity), and Squalene monooxygenase. The residues shown in bold represent the Squalene monooxygenase (this target $\in \mathscr{T}_{25}^{\text{antifungal}}$) amino acids involved in the same type of interaction with the tested and reference drugs.

$\mathscr{T}_{25}^{\text{antifungal}}$ **Squalene monooxygenase**								
Drug name	Drug role	Lowest free energy of binding [kcal/mol]	Estimated inhibition constant [Temp 298.15 K]	Conventional hydrogen bond	Carbon hydrogen bond	Alkyl interaction	Pi-Alkyl interaction	Interactive amino acid residues (Van der Waals interaction)
**Meprobamate**	Repositioning hint (No reported interaction with the target)	-2.67	11.01 mM	GLY_{A164} PHE_{A166} GLY_{A418} GLY_{A420} MET_{A421}	--	**VAL_{A163}** LEU_{A287}	--	ILE_{A162}, **GLU_{A165}**, LEU_{A167}, GLN_{A168}, PHE_{A306}, LEU_{A333}, **TYR_{A335}**, LEU_{A345}, MET_{A388}, PRO_{A389}, **ASP_{A408}**, **ARG_{A413}**, PRO_{A415}, GLY_{A419}, THR_{A422}
Naftifine	Reference drug (inhibitor)	-7.47	0.0034 mM	--	PRO_{B415}	VAL_{B163} ALA_{B322} LEU_{B333} MET_{B421}	--	ILE_{B162}, GLY_{A164}, **GLU_{A165}**, PHE_{B166}, GLN_{B168}, TYR_{B195}, PHE_{B306}, GLU_{B323}, LEU_{B324}, ILE_{B334}, **TYR_{B335}**, LEU_{B345}, LEU_{B416}, THR_{B417}, GLY_{B418}, GLY_{B419}, GLY_{B420}, LEU_{B509}
Tolnaftate	Reference drug (inhibitor)	-6.75	0.0113 mM	PRO_{A389}	PRO_{A389}	VAL_{A133} ILE_{A162} **VAL_{A163}** LEU_{A345} MET_{A388}	HIS_{A226} PHE_{A306}	ARG_{A161}, GLY_{A164}, **GLU_{A165}**, ILE_{A230}, GLY_{A286}, LEU_{A287}, **TYR_{A335}**, ALA_{A390}, SER_{A391}, **ASP_{A408}**, MET_{A412}, **ARG_{A413}**, HIS_{A414}, GLY_{A420}, MET_{A421}
Fosinopril	Reference drug (No reported interaction with the target)	-4.25	0.7694 mM	--	--	LEU_{A547} LEU_{A554} LEU_{B554} CYS_{A558} CYS_{B558}	--	PRO_{A544}, ARG_{A545}, LEU_{A548}, TYR_{B566}, GLY_{A551}, GLY_{B551}, ALA_{A552}, SER_{B559}, TYR_{A555}, TYR_{B555}, PRO_{B563}
Furosemide	Reference drug (No reported interaction with the target)	-5.36	0.1186 mM	HIS_{B198} GLU_{B205} ASP_{B370}	--	LYS_{B203}	--	ASP_{B199}, GLN_{B200}, GLU_{B201}, SER_{B204}, PHE_{B317}, PRO_{B366}, GLN_{B367}, ILE_{B368}, PRO_{B369}, HIS_{B371}, LYS_{B373}

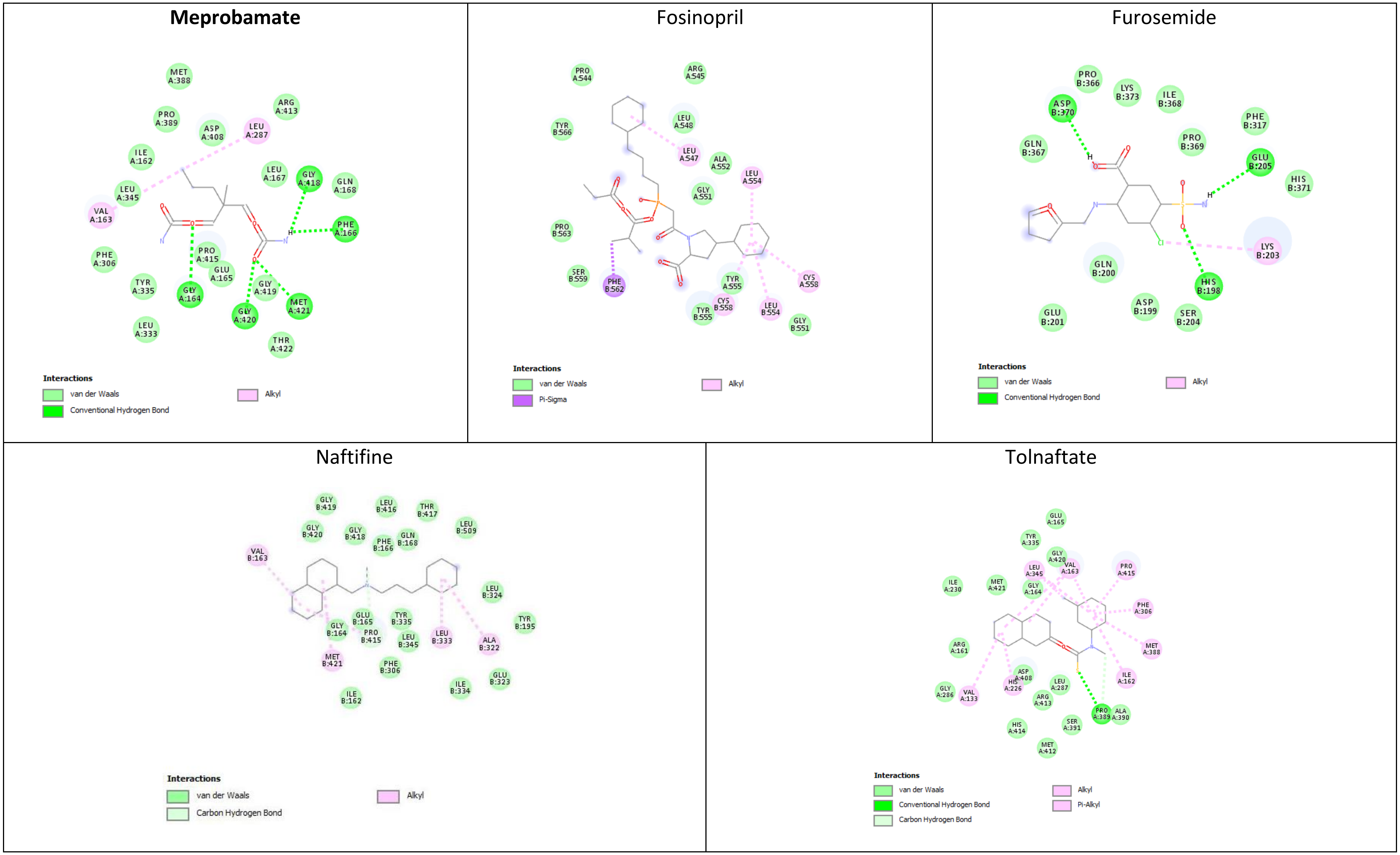

Figure S10. The 2D maps generated by the molecular docking simulation, for interactions of Meprobamate, Fosinopril, and Furosemide (i.e., $d_t^{antifungal}$ drugs), Naftifine and Tolnaftate (reference drugs from $\mathscr{D}_{25}^{antifungal}$ with already documented antifungal activity) with the amino acid residues in Squalene monooxygenase (this target $\in \mathscr{T}_{25}^{antifungal}$). The docking software places the 2D chemical representation of the drug molecule in the center of each square. The colored disks represent the amino acids surrounding the drug molecule, while the dotted lines represent the interactions between the target's amino acids and the drug molecule. The maps also indicate the amino acids that establish van der Waals interactions with the drug; however, for clarity, these interactions are not represented.

Table S11. A comparison of the molecular interactions between Meprobamate (i.e., the repositioning hint – $d_h^{\text{antifungal}}$), Clotrimazole (a $d_{25}^{\text{antifungal}}$ drug from $\mathscr{D}_{25}^{\text{antifungal}}$ reference drugs with documented antifungal activity), Nystatin and Natamycine (these drugs $\in \mathscr{D}_{\overline{25}}^{\text{antifungal}}$ reference drugs with well-documented antifungal activity), Fosinopril and Furosemide (these drugs $\in \mathscr{D}_{n}^{\text{antifungal}}$ reference drugs, with no reported antifungal activity) and Ergosterol (this target $\in \mathscr{T}_{\overline{25}}^{\text{antifungal}}$).

$\mathscr{T}_{\overline{25}}^{\text{antifungal}}$ **Ergosterol**				
	Drug role	Lowest free energy of binding [kcal/mol]	Estimated inhibition constant [Temp 298.15 K]	Description of drug-target molecular interaction
**Meprobamate**	Reference drug (No reported interaction with the target)	**-3.48**	**2.79 mM**	4 hydrophobic alkyl/alkyl interaction that involves the pentyl radical (meprobamat) and the six-atom cycles (ergosterol)
Clotrimazole	Reference drug (inhibitor)	-4.06	1.05 mM	8 hydrophobic alkyl/alkyl interaction that involves the 3 benzene rings (clotrimazole) and the six-atom cycles (ergosterol)
Nystatin	Repositioning hint (No reported interaction with the target)	-3.98	1.21 mM	1 hydrogen bond between –1-OH of nystatin (oxanic cycle) and –OH (ergosterol)
Natamycin	Reference drug (inhibitor)	-6.24	0.0265 mM	2 hydrogen bond between -3,4—dihydroxy of natamycine (methyloxan cylce) and –OH (ergosterol)
Fosinopril	Reference drug (No reported interaction with the target)	-3.79	1.67 mM	10 hydrophobic alkyl/alkyl interaction that involves all three cycles (fosinopril) and the five-atom cycles and 18-CH_3 (ergosterol)
Furosemide	Reference drug (No reported interaction with the target)	-4.01	1.15 mM	1 hydrophobic alkyl/alkyl interaction between chlorine (furosemide) and -OH (ergosterol)

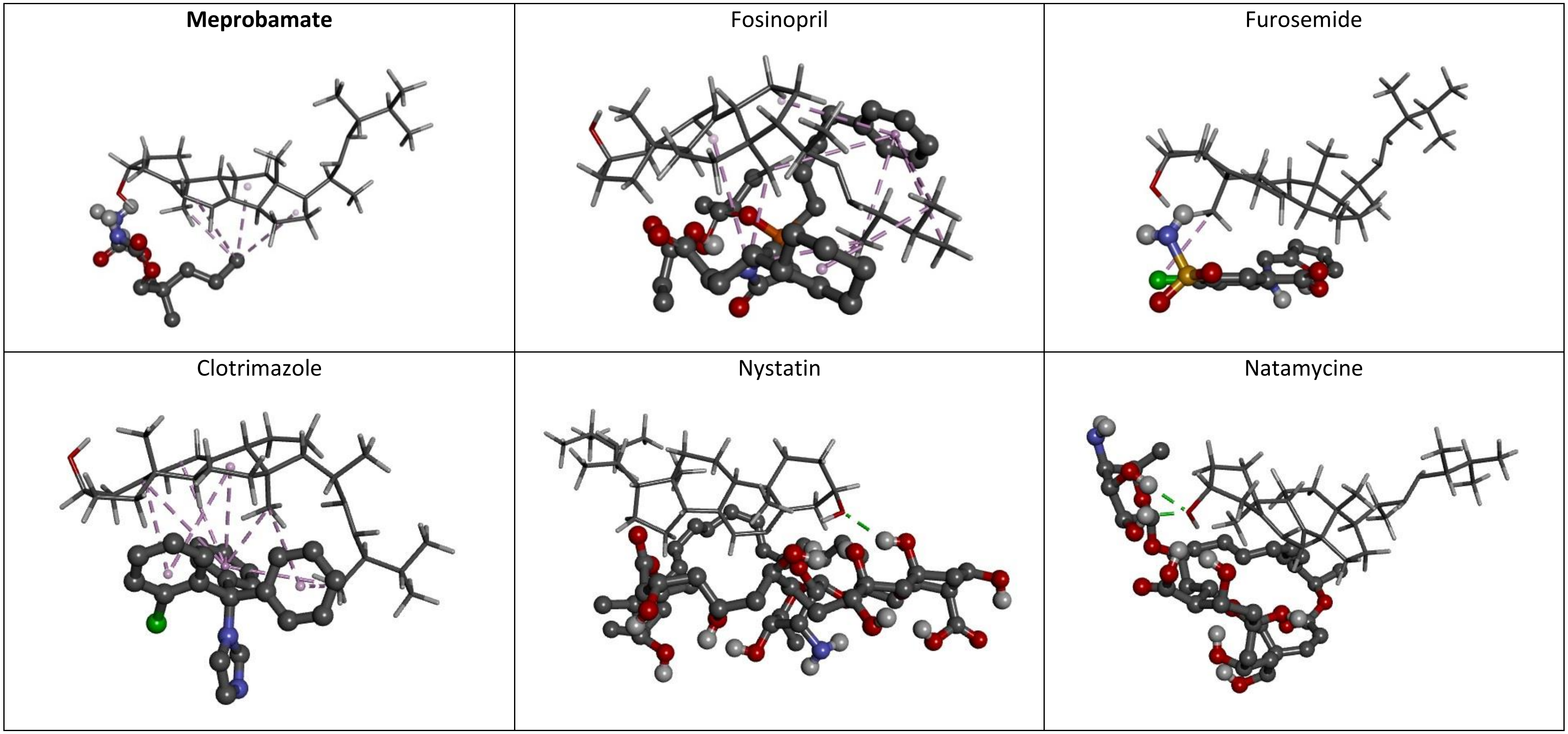

Figure S11. Structure views of the 3D-complexes between Meprobamate (i.e., the repositioning hint – $d_h^{\text{antifungal}}$), Clotrimazole (a $d_{25}^{\text{antifungal}}$ drug from $\mathscr{D}_{25}^{\text{antifungal}}$ reference drug with documented antifungal activity), Nystatin and Natamycine (these reference antifungals drugs $\in \mathscr{D}_{25}^{\text{antifungal}}$), Fosinopril and Furosemide (reference drugs $\in \mathscr{D}_n^{\text{antifungal}}$, with no reported antifungal activity) with Ergosterol, which is a steroidal target from $\mathscr{T}_{25}^{\text{antifungal}}$. The drug molecules are represented by the ball-and-stick molecular models, while the chemical structure in gray lines is for Ergosterol. The purple dashed lines represent the hydrophobic alkyl/alkyl interactions between Meprobamate, Clotrimazole, Fosinopril, and Furosemide with the target, and the green lines are for the hydrogen bonds of Nystatin and Natamycine with the target.

Table S12. The comparison of the molecular interactions of Meprobamate (i.e., the repositioning hint – $d_h^{\text{antifungal}}$), Ciclopirox (a $d_{\overline{25}}^{\text{antifungal}}$ drug from $\mathscr{D}_{\overline{25}}^{\text{antifungal}}$ reference drugs with documented antifungal activity), Fosinopril and Furosemide ($\in \mathscr{D}_n^{\text{antifungal}}$ reference drugs, with no reported antifungal activity) and Sodium/potassium-transporting ATPase subunit alpha (this target $\in \mathscr{T}_{\overline{25}}^{\text{antifungal}}$). There are no target amino acid involved in the same type of interaction with the test and reference drugs.

$\mathscr{T}_{\overline{25}}^{\text{antifungal}}$ **Sodium/potassium-transporting ATPase subunit alpha**								
Drug name	Drug role	Lowest free energy of binding [kcal/mol]	Estimated inhibition constant [Temp 298.15 K]	Conventional hydrogen bond	Carbon hydrogen bond	Alkyl interaction	Pi-Alkyl interaction	Interactive amino acid residues (Van der Waals interaction)
**Meprobamate**	Repositioning hint (No reported interaction with the target)	-2.47	15.37 mM	GLN_{B69} ASN_{B282}	ALA_{B73}	VAL_{B183}	PHE_{B186}	ASP_{B70}, VAL_{B72}, PRO_{B74}, PRO_{B75}, LEU_{B184}, GLY_{B185}, LYS_{B187}, GLU_{B281}, ILE_{B283}
Ciclopirox	Reference drug (binder)	-5.38	0.1145 mM	GLN_{D69} ALA_{D73}	--	ALA_{D73} PRO_{D74} (3)	PHE_{E19} PHE_{D186}	TYR_{E20}, TYR_{E21}, ASP_{D70}, ARG_{D71}, VAL_{D72}, PRO_{D75}, VAL_{D183}, GLU_{D281}, ASN_{D282}, ILE_{D283}
Fosinopril	Reference drug (No reported interaction with the target)	-3.04	5.92 mM	SER_{C988}	--	VAL_{C928} (2) VAL_{C937} MET_{C942} (2) CLR_{C1107}	--	VAL_{C921}, VAL_{C922}, TRP_{C924}, ALA_{C925}, LYS_{C931}, PHE_{C938}, ILE_{C948}, LEU_{C951}, PHE_{C952}, PHE_{C985}, LEU_{C989}, PHE_{C992}
Furosemide	Reference drug (No reported interaction with the target)	-4.11	0.9668 mM	GLN_{C274} THR_{C359} LYS_{C719} ASP_{C722} ASP_{C740} (2)	LEU_{C270} GLU_{C271} PRO_{C276}	ALA_{C356}	--	GLY_{C272}, THR_{C275}, LYS_{C352}, ASN_{C353}, GLU_{C355}, SER_{C718}, ALA_{C721}, ILE_{C723}, GLY_{C724}, GLN_{C737}, ALA_{C738}, ALA_{C739}

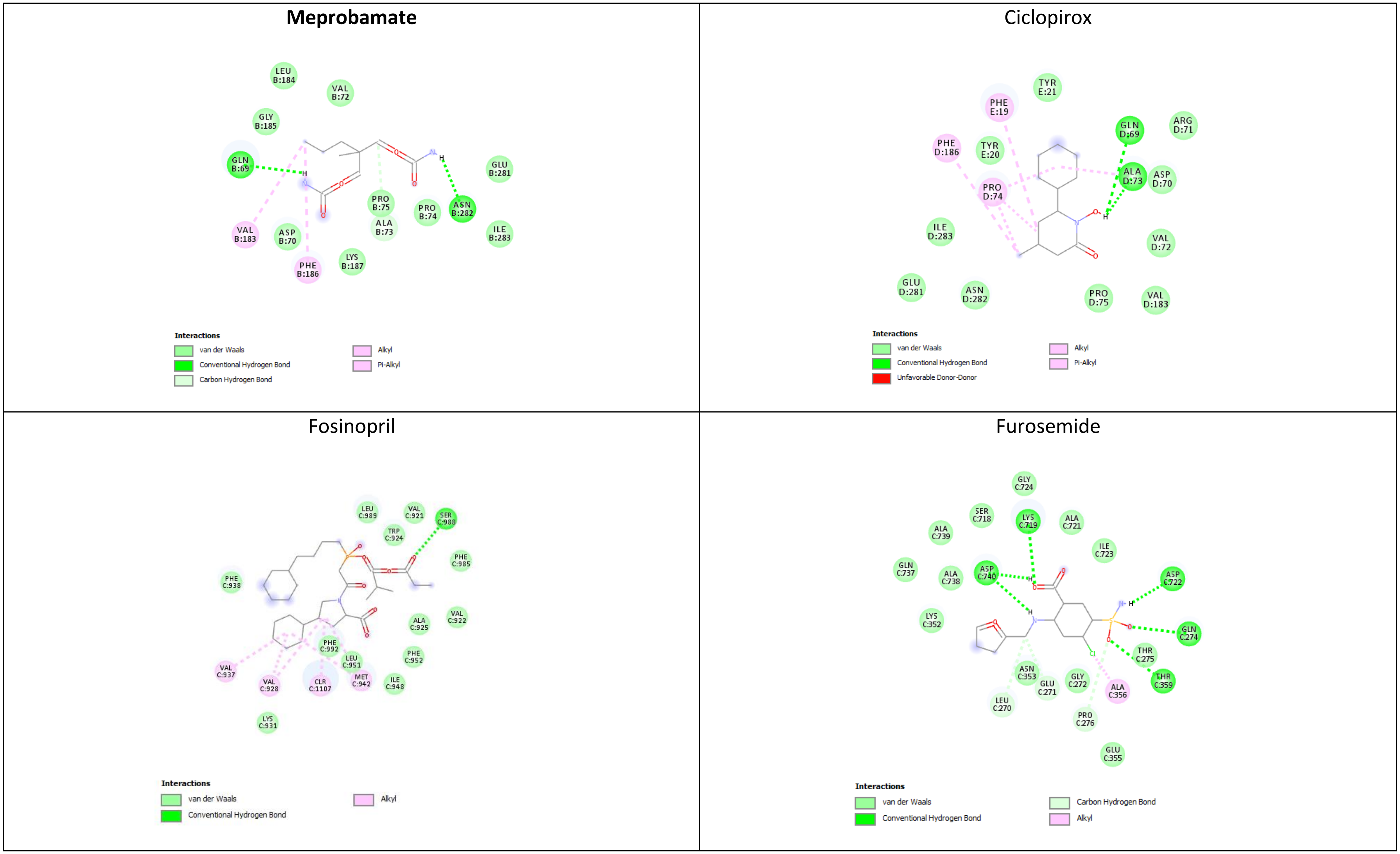

Figure S12. The 2D diagrams generated by the molecular docking simulation for interactions of Meprobamate, Fosinopril, and Furosemide (i.e., $d_t^{\text{antifungal}}$ drugs), Ciclopirox (i.e., a reference drug from $\mathscr{D}_{25}^{\text{antifungal}}$) with the amino acid residues in Sodium/potassium-transporting ATPase subunit alpha (this target $\in \mathscr{T}_{25}^{\text{antifungal}}$). The 2D chemical representation of the drug molecule is in the center of each square. The colored disks represent the amino acids surrounding the drug molecule, while the dotted lines represent the interactions between the target's amino acids and the drug molecule. The diagrams also indicate the amino acids that establish van der Waals interactions with the drug; however, for clarity, these interactions are not represented.

Table S13. The comparison of the molecular interactions between Meprobamate (i.e., the repositioning hint – $d_h^{\text{antifungal}}$), Griseofulvin (a $d_{\overline{25}}^{\text{antifungal}}$ drug from $\mathscr{D}_{\overline{25}}^{\text{antifungal}}$ reference drugs with documented antifungal activity), Fosinopril and Furosemide ($\in \mathscr{D}_n^{\text{antifungal}}$ reference drugs, with no reported antifungal activity), and Tubulin (this target $\in \mathscr{T}_{\overline{25}}^{\text{antifungal}}$). The residues shown in bold represent the Tubulin amino acids involved in the same type of interaction with the test and reference drugs.

$\mathscr{T}_{\overline{25}}^{\text{antifungal}}$ **Tubulin**								
Drug name	Drug role	Lowest free energy of binding [kcal/mol]	Estimated inhibition constant [Temp 298.15 K]	Conventional hydrogen bond	Carbon hydrogen bond	Alkyl interaction	Pi-Alkyl interaction	Interactive amino acid residues (Van der Waals interaction)
**Meprobamate**	Repositioning hint (No reported interaction with the target)	-2.99	6.42 mM	VAL$_{B260}$ TRP$_{B346}$	--	PRO$_{B261}$ **ILE$_{B347}$**	--	ALA$_{B256}$, VAL$_{B257}$, **ASN$_{B258}$**, **MET$_{B259}$**, **LEU$_{B313}$**, THR$_{B314}$, **PRO$_{B348}$**, **ASN$_{B349}$**, LYS$_{A401}$, **ALA$_{A403}$**, **PHE$_{A404}$**, **TYR$_{B435}$**
Griseofulvin	Reference drug (inhibitor)	-6.14	0.0316 mM	THR$_{B314}$ LYS$_{A401}$	VAL$_{A181}$ VAL$_{B257}$	**ILE$_{B347}$**	--	VAL$_{A182}$, PRO$_{A184}$, **ASN$_{B258}$**, **MET$_{B259}$**, VAL$_{B260}$, PRO$_{B261}$, **LEU$_{B313}$**, TRP$_{B346}$, **PRO$_{B348}$**, **ASN$_{B349}$**, ASN$_{B350}$, LEU$_{A397}$, MET$_{A398}$, **ALA$_{A403}$**, **PHE$_{A404}$**, TYR$_{B432}$, **TYR$_{B435}$**
Fosinopril	Reference drug (No reported interaction with the target)	-4.28	0.7279 mM	THR$_{B145}$ GLY$_{B146}$ ASP$_{B179}$ THR$_{B180}$ GTP$_{B502}$	THR$_{B145}$	PRO$_{B173}$	HIS$_{B139}$	ALA$_{B9}$, GLY$_{B10}$, CYS$_{B12}$, ASN$_{B101}$, LEU$_{B137}$, GLY$_{B142}$, GLY$_{B143}$, GLY$_{B144}$, SER$_{B147}$, GLY$_{B150}$, SER$_{B170}$, VAL$_{B171}$, VAL$_{B172}$, GLU$_{B183}$, ASN$_{B206}$
Furosemide	Reference drug (No reported interaction with the target)	-5.09	0.1861 mM	HIS$_{A309}$ GLN$_{A342}$ (2)	LYS$_{A311}$	--	--	PHE$_{A296}$, PRO$_{A307}$, ARG$_{A308}$, GLY$_{A310}$, TYR$_{A312}$, ILE$_{A335}$, LYS$_{A338}$, THR$_{A340}$, ILE$_{A341}$, PHE$_{A343}$

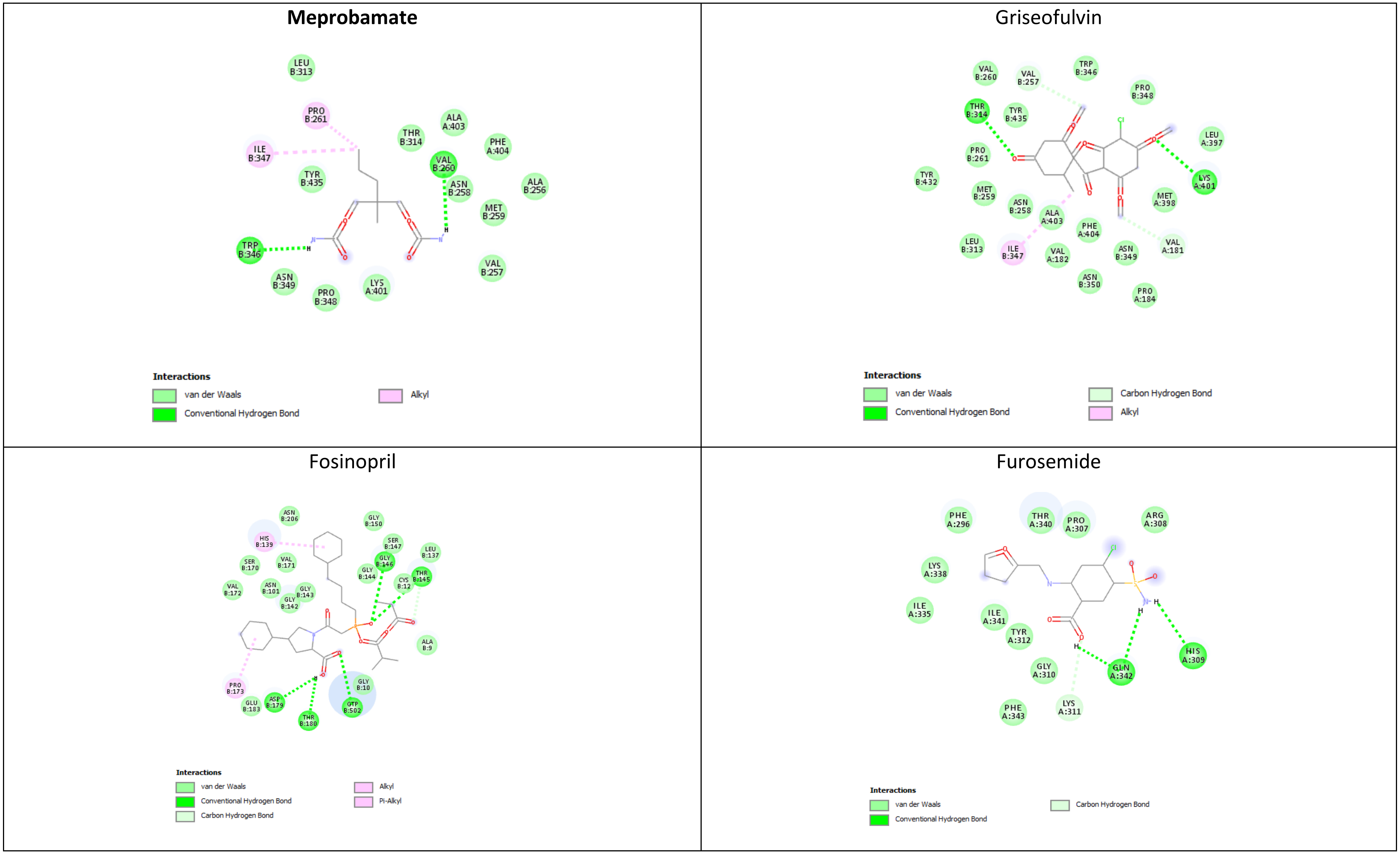

Figure S13. The 2D maps generated by the molecular docking simulation for interactions between Meprobamate, Fosinopril, Furosemide (i.e., $d_t^{\text{antifungal}}$ drugs), Griseofulvin (this reference drug $\in \mathscr{D}_{25}^{\text{antifungal}}$) and the amino acid residues in Tubulin (this target $\in \mathscr{T}_{25}^{\text{antifungal}}$). The 2D chemical representation of the drug molecule is in the center of each square. The colored disks represent the amino acids surrounding the drug molecule, while the dashed lines represent the interactions between the target's amino acids and the drug molecule. The maps also indicate the amino acids that establish van der Waals interactions with the drug; however, for clarity, these interactions are not represented.

5. Molecular docking results interpretation

5.1 Azelaic acid

Table S1 & Figure S1

The free energy of the complex between Progesterone and **Estrogen receptor alpha** is -5.89 kcal/mol, Abiraterone and **Estrogen receptor alpha** is -7.11 kcal/mol, whereas that of the complex between Azelaic acid and Estrogen receptor alpha is -4.48 kcal/mol, showing that the Progesterone and Abiraterone complexes have higher stability than that of the Azelaic acid complex. Azelaic acid and Progesterone bind to the target through 8 amino acids, but Azelaic acid establishes an identical type of interaction with only one amino acid. Azelaic acid and Abiraterone bind to the target through 7 amino acids, but Azelaic acid establishes an identical type of interaction with only one amino acid. Fosinopril and Furosemide interact with no amino acids in the target. The results indicate a low similarity between Progesterone (i.e., a reference drug in $\mathscr{D}_6^{\text{anticancer}}$) and $d_h^{\text{anticancer}}$ Azelaic acid. We also notice a clear difference between the $\mathscr{D}_n^{\text{anticancer}}$ = {Fosinopril, Furosemide} and reference anticancer drugs in terms of interaction at the active binding site of Estrogen receptor beta.

Table S2 & Figure S2

The lowest free energy of the Azelaic acid-**Estrogen receptor beta** complex is -3.11 kcal/mol, Abiraterone-Estrogen receptor beta complex is -7.90 kcal/mol, and of the Progesterone-Estrogen receptor beta complex is -8.68 kcal/mol, indicating that the Progesterone and Abiraterone complexes have higher stability than that of Azelaic acid. Both Progesterone and Azelaic acid bind the same 15 amino acids, and 5 out of 15 interactions are of the same type. Azelaic acid and Abiraterone bind to the target through the same 4 amino acids, and Azelaic acid establishes identical type of interactions with 3 amino acids. Fosinopril and Furosemide interact with no amino acid in the active site of the Estrogen receptor beta.

Table S3 & Figure S3

The free energy of the complex between Azelaic acid and **Progesterone receptor** is -4.54 kcal/mol, that of the complex between Progesterone and Progesterone receptor is -11.17 kcal/mol, and that of the complex between Abiraterone and Progesterone receptor is -11.90 kcal/mol, thus indicating higher stability for the Abiraterone and Progesterone complexes. However, Azelaic acid and Progesterone similarly bind to the target, as both drugs interact with the same 12 amino acids in the target, and 7 out of 12 interactions are of the same type; Azelaic acid and Abiraterone bond to this target with the same 7 amino acids, but only 2 out of these 7 interactions are of the same type. Fosinopril establishes only one van der Waals interaction with Progesterone receptor, and Furosemide do not interact with any amino acids in the target. These results indicate a similarity between Progesterone (i.e., a reference drug $\mathscr{D}_6^{\text{anticancer}}$) and $d_h^{\text{anticancer}}$ Azelaic acid. On the other hand, we notice a clear difference between the $\mathscr{D}_n^{\text{anticancer}}$ = {Fosinopril, Furosemide} and Progesterone in terms of interaction at the active binding site of Progesterone receptor.

Table S4 & Figure S4

The lowest free energy of the complex between Azelaic acid and **Steroid 17-alpha-hydroxylase/17,20 lyase** is -8.49 kcal/mol, that of the complex between Progesterone and Steroid 17-alpha-hydroxylase/17,20 lyase is -8.72 kcal/mol, and that of the complex between Abiraterone and Steroid 17-alpha-hydroxylase/17,20 lyase is -8.99 kcal/mol, suggesting a very similar stability of the three complexes. DrugBank lists Progesterone as a substrate and inhibitor, and Abiraterone as an inhibitor of Steroid 17-alpha-hydroxylase/17,20 lyase. On the other hand, our results indicate a high similarity between the repositioning hint (i.e., Azelaic acid) and the reference anticancer drugs (i.e., Progesterone and Abiraterone) in terms of the inhibition constant, as these values rendered by the docking simulation are 600.71 nM, 406.22 nM, and 402.33 nM for Azelaic acid, Progesterone, and Abiraterone, respectively. Azelaic acid and Progesterone similarly bind to the target, as both drugs interact with the same 8 amino acids in the target, and 5 out of 8 interactions are of the same type. Azelaic acid and Abiraterone interact with the same 5 amino acids in the target, and 2 interactions are of the same type. Moreover, our docking simulation results are in line with results of C. Avendaño (Carme nAvendaño, J. CarlosMenéndez. Chapter 3 - Anticancer Drugs That Modulate Hormone Action. Medicinal Chemistry of Anticancer Drugs, Second Edition, 2015, p. 81-131) and **N.M. DeVore** (Natasha M. DeVore & Emily E. Scott. Structures of cytochrome P450 17A1 with prostate cancer drugs abiraterone and TOK-001. Nature. 2012; 482(7383): 116–119), which report the covalent bonding of Abiraterone and Steroid 17-alpha-hydroxylase/17,20 lyase (a cysteinato-heme enzyme that belongs to the cytochrome P450 superfamily). Precisely, Abiraterone forms a coordinate covalent bond of the pyridine nitrogen at C_{17} with heme iron of this target (Natasha M. DeVore & Emily E. Scott. Structures of cytochrome P450 17A1 with prostate cancer drugs abiraterone and TOK-001. Nature. 2012; 482(7383): 116–119). Fosinopril and Furosemide do not interact with any amino acids in this target. We notice a similarity between Progesterone (i.e., a reference drug $\mathscr{D}_6^{\text{anticancer}}$) and $d_h^{\text{anticancer}}$ Azelaic acid in terms of number and type of interactions with the amino acids in the target. We notice a clear difference between the $\mathscr{D}_n^{\text{anticancer}}$ = {Fosinopril, Furosemide} and the anticancer reference drugs (i.e., Progesterone and Abiraterone) in terms of interaction at the active binding site of Steroid 17-alpha-hydroxylase/17,20 lyase. These results suggest that Azelaic acid is a promising candidate for further in silico, in vitro and in vivo investigations of its potential anticancer effects.

Table S5 & Figure S5

The lowest free energy of the Azelaic acid - **Androgen receptor** complex is -5.01 kcal/mol, whereas the Progesterone - Anrogen receptor complex is more stable than the Azelaic acid - Androgen receptor complex (-10.28 kcal/mol). Azelaic acid and Progesterone interact similarly with Androgen receptor through a common set of 10 amino acids; 6 out of these interactions are of the same type. Fosinopril and Furosemide interact with none of the 11 amino acids in the active site of the target. Again, we notice a difference between the $\mathscr{D}_n^{\text{anticancer}}$ = {Fosinopril, Furosemide} and Progesterone in terms of interactions at the active binding site of Androgen receptor.

Table S6 & Figure S6

The lowest free energy of the Azelaic acid - **Mineralocorticoid receptor** complex is -4.49 kcal/mol, of the Progesterone - Mineralocorticoid receptor complex is -10.77 kcal/mol, and of the Abiraterone - Mineralocorticoid receptor is -8.08 kcal/mol, thus indicating that the Progesterone complex has higher stability than that of Abiraterone and Azelaic acid complexes. Progesterone and Azelaic acid bind the same 13 amino acids in

the target, and 4 out of 13 interactions are of the same type. Abiraterone, Fosinopril and Furosemide interact with no amino acid in the active site of the Mineralocorticoid receptor.

5.2 Meprobamate

Table S7 & Figure S7

The free energy of the complex between Meprobamate and **Lanosterol 14-alpha demethylase** is -2.77 kcal/mol, whereas that of the complex between Clotrimazole and Lanosterol 14-alpha demethylase is -7.15 kcal/mol, showing a higher stability for the Clotrimazole complex. However, Clotrimazole and Meprobamate similarly bind to the target, as both drugs interact with the same 10 amino acids in the target (7 out of 10 interactions are of the same type). Conversely, Fosinopril and Furosemide interact with none of these 10 amino acids in the target. On the other hand, we notice a clear difference between the $\mathscr{D}_n^{\text{antifungal}}$ = {Fosinopril, Furosemide} and Clotrimazole in terms of interaction at the active binding site of Lanosterol 14-alpha demethylase.

Table S8 & Figure S8

The lowest free energy of the Meprobamate - **Lanosterol synthase** complex is -3.23 kcal/mol, whereas the Oxiconazole - Lanosterol synthase complex is more stable than the Meprobamate-Lanosterol synthase (-6.24 kcal/mol). Oxiconazole and Meprobamate interact similarly with Lanosterol synthase through a common set of 11 amino acids, interactions of which 7 are of the same type. Fosinopril and Furosemide interact with none of the 11 amino acids in the active site of the target. Again, we have a clear difference between the $\mathscr{D}_n^{\text{antifungal}}$ = {Fosinopril, Furosemide} and Oxiconazole regarding the interaction at the active binding site of Lanosterol synthase.

Table S9 & Figure S9

The molecular docking results reveal that the complex Meprobamate - **Intermediate conductance calcium-activated potassium channel protein 4** has the lowest free energy of -1.02 kcal/mol. The complex between the reference antifungal drug Clotrimazole and the same target has the lowest free energy -3.60 kcal/mol. Examining the drug-target interaction, we notice that Meprobamate, Fosinopril, and Furosemide are different from Clotrimazole in the way they interact with the amino acids in the target active site.

Table S10 & Figure S10

For the Meprobamate - **Squalene monooxygenase** complex, the lowest free energy is -2.67 kcal/mol, while for the complexes of Tolnaftate and Naftifine with Squalene monooxygenase are -6.75 kcal/mol and 7.47 kcal/mol, respectively; this means that the reference drug-target complexes are more stable than the Meprobamate complex. Meprobamate and Tolnaftate interact with the same 15 amino acids in the target (of which 5 are of the same type). Concurrently, the interaction of both Meprobamate and Naftifine with Squalene monooxygenase shares only 3 amino acids, of which 2 are of the same type. Fosinopril and Furosemide interact with no amino acid in the active site of the target. According to our docking results, Meprobamate's behavior in terms of binding to the target Squalene monooxygenase is more similar to Tolnaftate than to Naftifine.

Table S11 & Figure S11

The interaction with the (non-protein) target **Ergosterol** is similar for Meprobamate (the repositioning hint) and Clotrimazole ($\in \mathscr{D}_{25}^{\text{antifungal}}$), through weak hydrophobic interactions with the Ergosterol six-atoms cycles. The Clotrimazole-Ergosterol complex (-4.06 kcal/mol) is more stable than the Meprobamate-Ergosterol complex (-3.48 kcal/mol). Of note, the reference antifungal drugs Nystatin and Natamycine ($\mathscr{D}_{25}^{\text{antifungal}}$) interact differently with Ergosterol, as they form hydrogen bonds with the target's hydroxyl group, while Fosinopril and Furosemide establish hydrophobic interactions with the five-atom cycle of Ergosterol.

Table S12 & Figure S12

The molecular docking of Meprobamate with **Sodium/potassium-transporting ATPase subunit alpha**, as well as of Ciclopirox with the same target, reveals that the former complex has lower stability (-2.47 kcal/mol) than the latter (-5.38 kcal/mol). Furthermore, Meprobamate and Ciclopirox do not interact with common amino acids in the active site of the target. Fosinopril and Furosemide do not interact with the amino acids in the active site of the target.

Table S 13 & Figure S13

The lowest free energy of the Meprobamate - **Tubulin** complex is -2.99 kcal/mol, and of the Griseofulvin - Tubulin complex is -6.14 kcal/mol, indicating that the Griseofulvin complex has much higher stability than that of Meprobamate. Griseofulvin and Meprobamate similarly bind to the target, as both drugs interact with the same 15 amino acids (9 out of 15 interactions are of the same type). Fosinopril and Furosemide interact with none of the 15 amino acids in the active site of the target.

6. The graphical representations of the docked complexes

Figure S14 The graphical representation of the docked complex between the antifungal reference drug Clotrimazole and the target Lanosterol 14-alpha demethylase

Figure S15 The graphical representation of the docked complex between the antifungal repositioning hint Meprobamate and the target Lanosterol 14-alpha demethylase

Figure S16 The graphical representation of the docked complex between the anticancer reference drug Progesterone and the target Progesterone receptor

Figure S17 The graphical representation of the docked complex between the anticancer repositioning hint Azelaic acid and the target Progesterone receptor

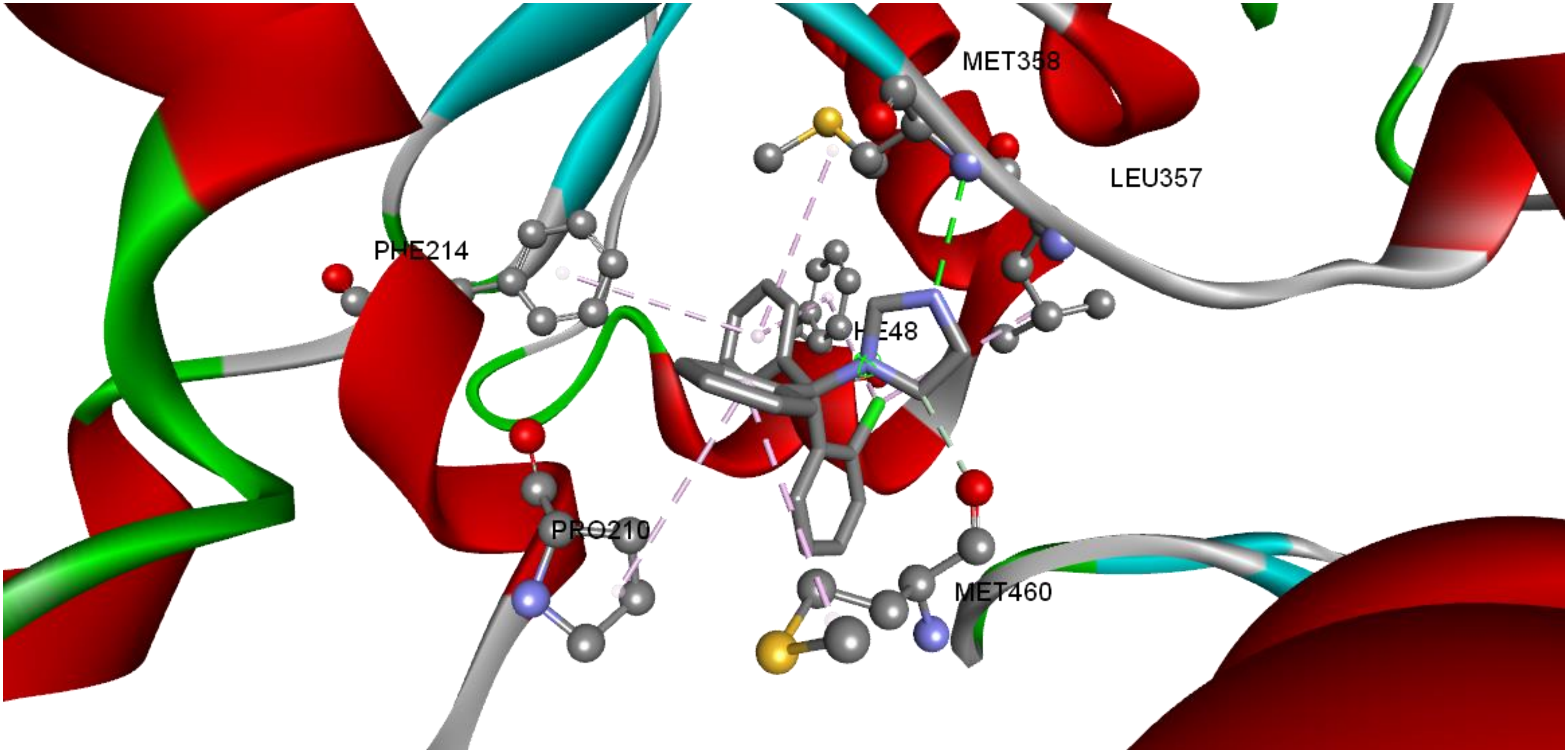

Figure S14 Molecular interactions analysis of Clotrimazole (the reference antifungal drug) with target Lanosterol 14-alpha demethylase. The docked complex of Lanosterol 14-alpha demethylase with Clotrimazole emphasize the molecular interactions of Meprobamate towards the active site of Lanosterol 14-alpha demethylase; the green dashed lines represent conventional and carbon hydrogen bonds, and the pink dashed lines represent the alkyl and pi-alkyl interactions. The flat ribbon represents the protein target, from which the interacting amino acid residues append. The figure displays the drug molecules (figured as ball-and-stick models) in the binding pocket of the target.

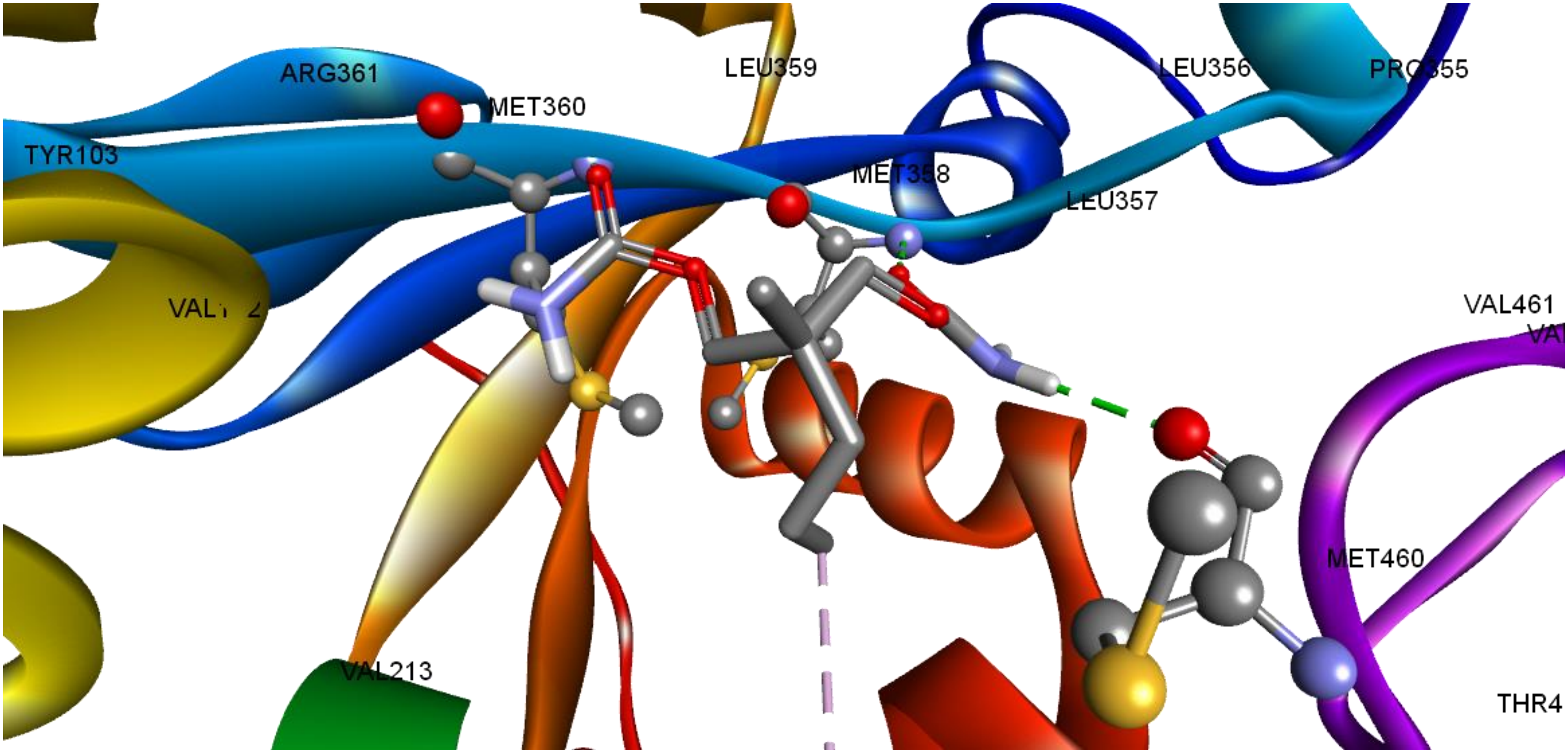

Figure S15 Molecular interactions analysis of Meprobamate (the repositioning hint) with target Lanosterol 14-alpha demethylase. The docked complex of Lanosterol 14-alpha demethylase with Meprobamate emphasize the molecular interactions of Meprobamate towards the active site of Lanosterol 14-alpha demethylase. The green dashed lines represent conventional hydrogen bonds, and the pink dashed lines represent the alkyl interactions. The flat ribbon represents the protein target, from which the interacting amino acid residues append. The figure displays the drug molecule (figured as ball-and-stick models) in the binding pocket of the target.

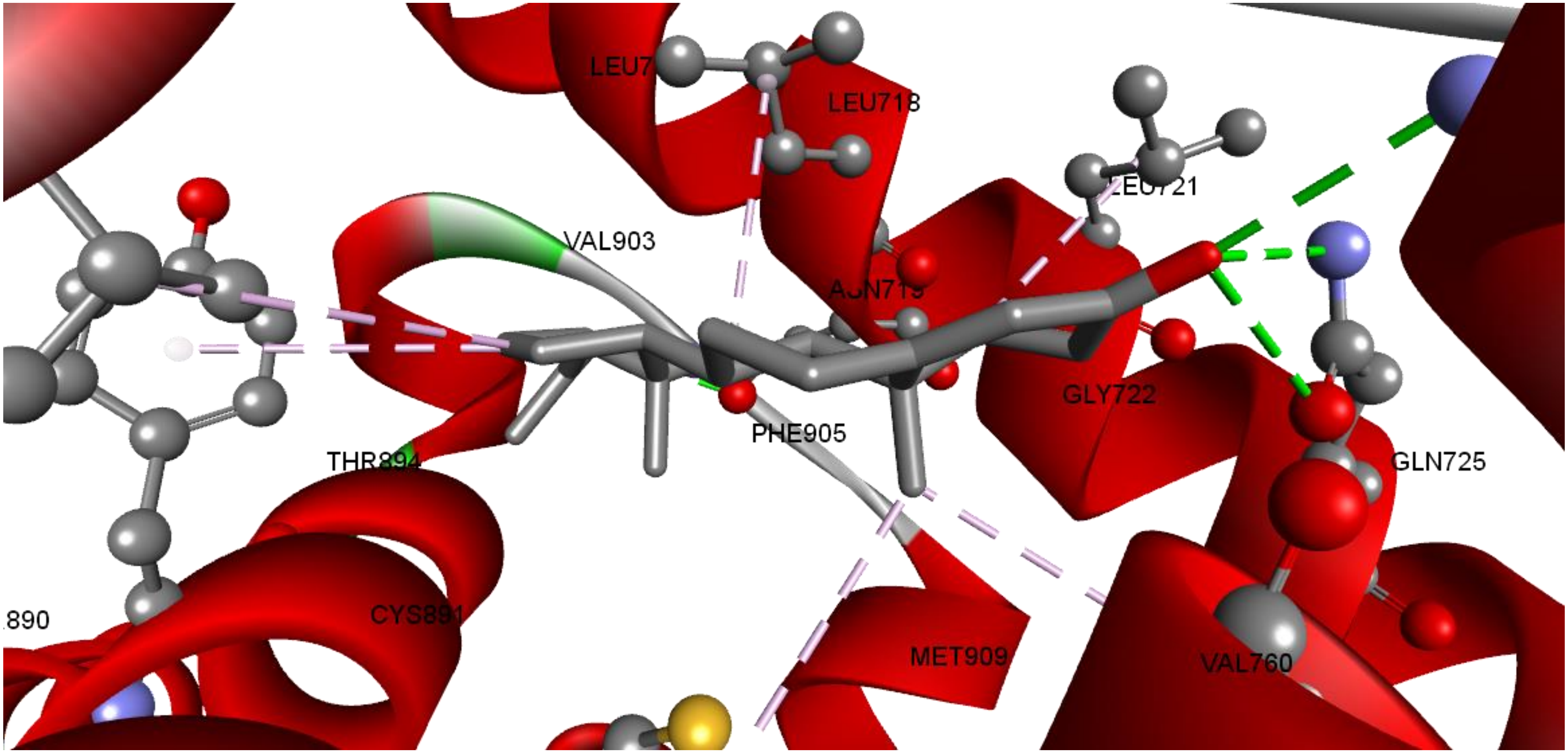

Figure S16 Molecular interactions analysis of Progesterone (the reference anticancer drug) with target Progesterone receptor. The docked complex of Progesterone receptor with Progesterone emphasize the molecular interactions of Progesterone towards the active site of Progesterone receptor; the green dashed lines represent conventional hydrogen bonds, and the pink dashed lines represent the alkyl and pi-alkyl interactions. The flat ribbon represents the protein target, from which the interacting amino acid residues append. The figure displays the drug molecules (figured as ball-and-stick models) in the binding pocket of the target.

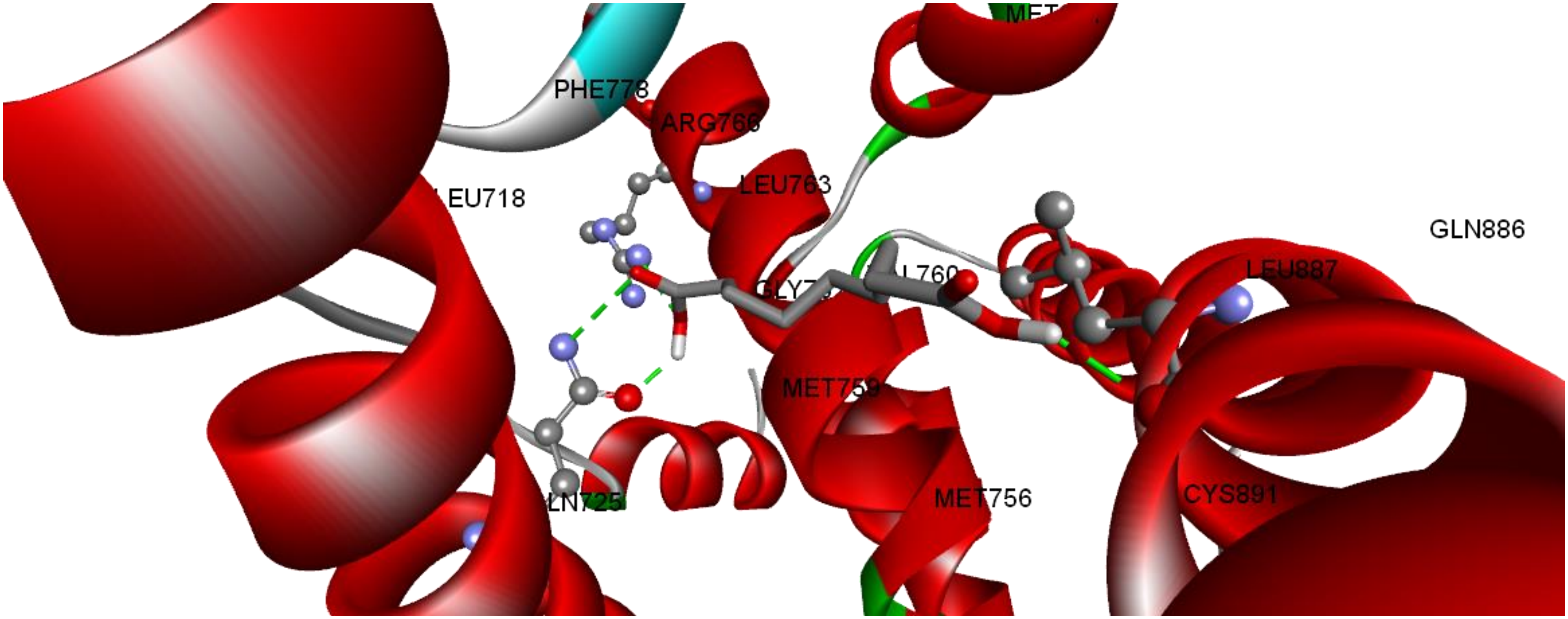

Figure S17 Molecular interactions analysis of Azelaic acid (the repositioning hint) with target Progesterone receptor. The docked complex of Progesterone receptor with Azelaic acid emphasize the molecular interactions of Azelaic acid towards the active site of Progesterone receptor. The green dashed lines represent conventional hydrogen bonds. The flat ribbon represents the protein target, from which the interacting amino acid residues append. The figure displays the drug molecule (figured as ball-and-stick models) in the binding pocket of the target.

7. Quantum chemical calculation

7.1 HOMO-LUMO energies for all ligands (i.e., drugs) in this manuscript

Graphical representation of HOMO orbitals in the studied molecules	Graphical representation of LUMO orbitals in the studied molecules	E_{HOMO} (eV)	E_{LUMO} (eV)	ΔE [(1)] (eV)	λ [(2)] (eV)	η [(3)] (eV)
**Azelaic acid**						
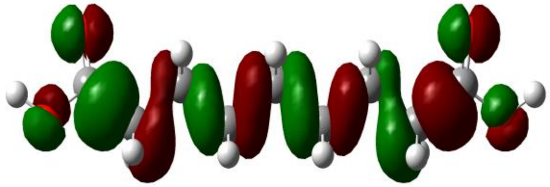	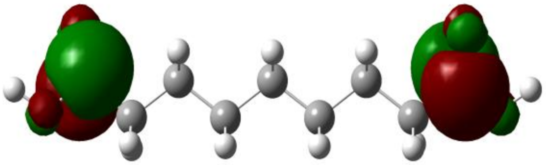	- 11.38467	0.917639	12.302309	5.2335	6.15115
**Progesterone**						
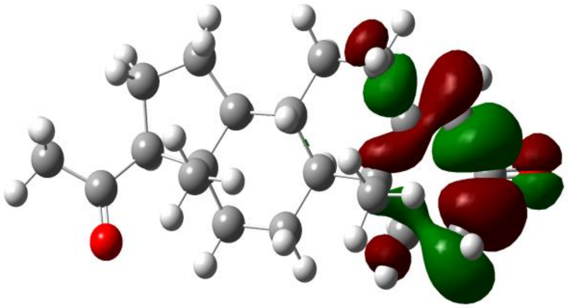	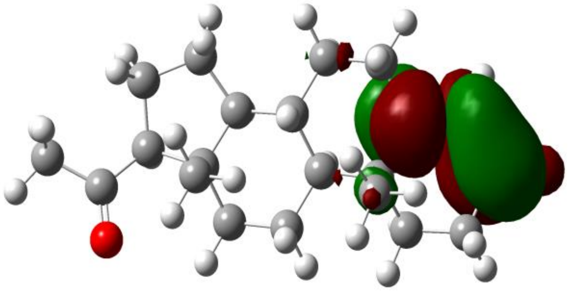	- 10.15986	- 0.097347	10.062512	5.1286	5.031255
**Abiraterone**						
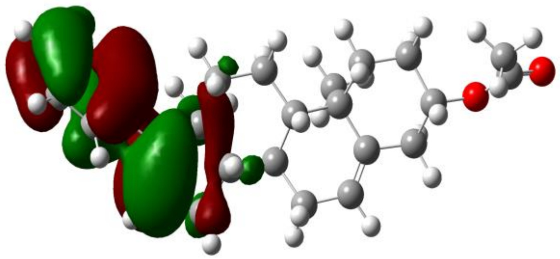	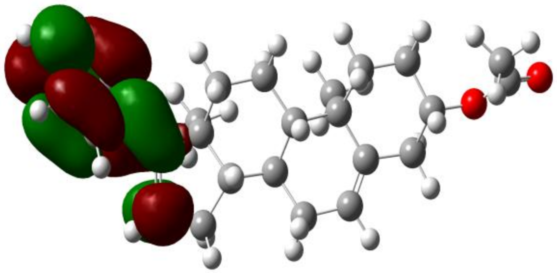	- 9.249274	- 0.309326	8.939947	4.7793	4.469973

**Meprobamate**						
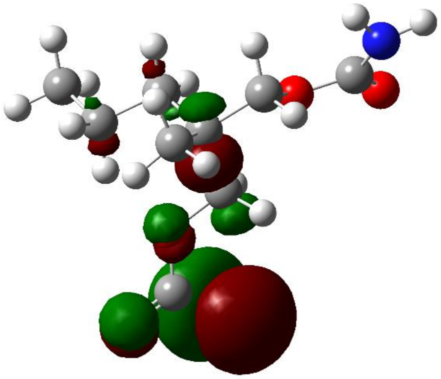	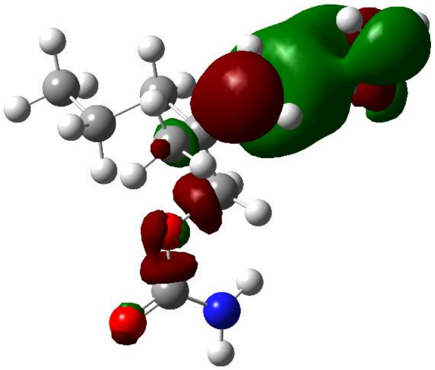	**-10.48988**	**0.867030**	**11.35691**	**4.811426**	**5.6784**
**Clotrimazole**						
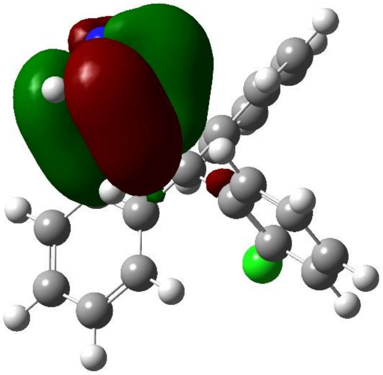	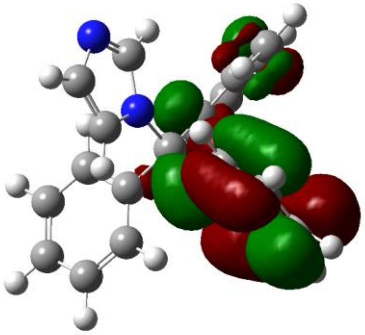	**- 9.280731**	**- 0.347823**	**8.932907**	**4.814275**	**4.292541**

**Oxiconazole**						
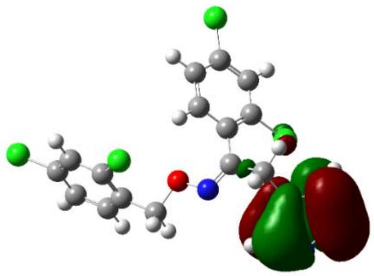	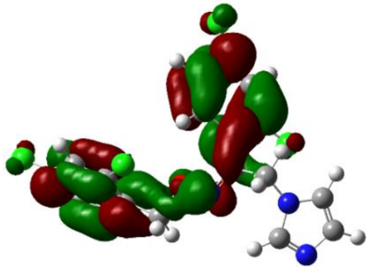	**- 9.344505**	**- 0.631010**	**8.713494**	**4.987755**	**4.356747**
**Naftifine**						
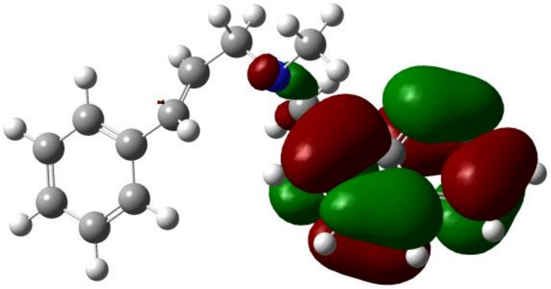	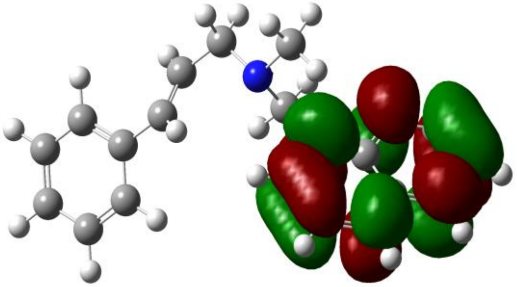	**- 8.649096**	**- 0.351178**	**8.297918**	**4.50013**	**4.148959**
**Tolnaftate**						
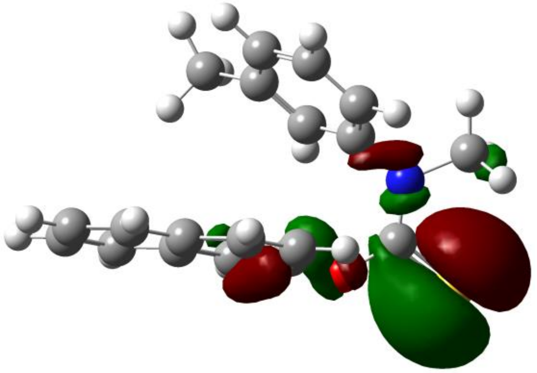	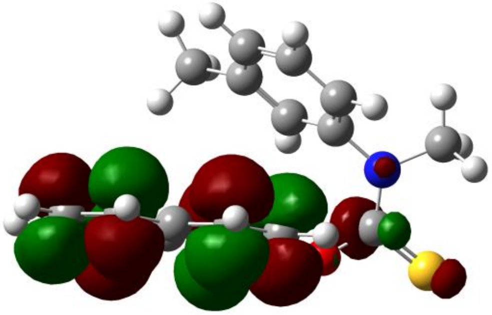	**- 8.685643**	**- 0.760219**	**7.925423**	**4.72293**	**3.962711**

**Nystatin**						
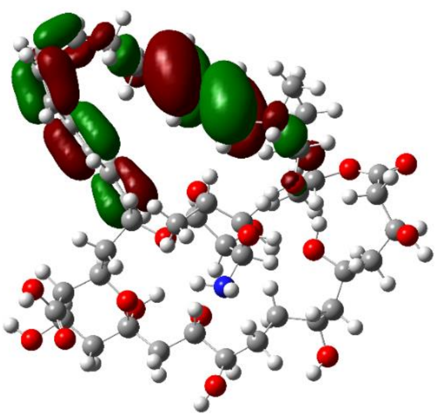	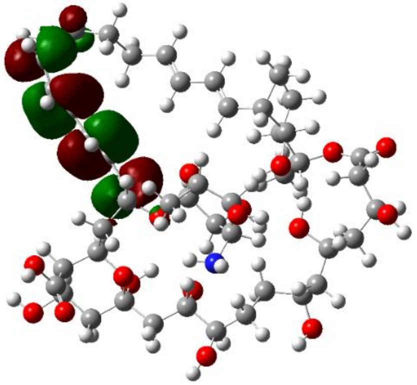	**- 8.714139**	**- 0.626526**	**8.087619**	**4.67033**	**3.7305**
**Natamycin**						
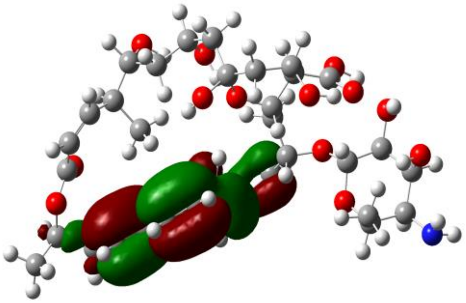	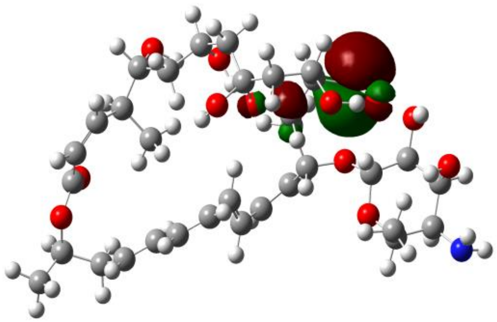	**- 9.110476**	**0.0363716**	**9.146847**	**4.53705**	**4.573423**
**Ciclopirox**						
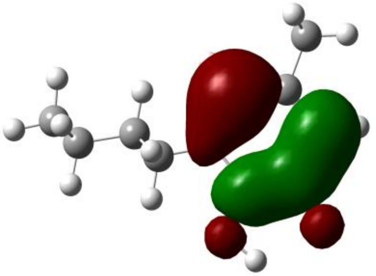	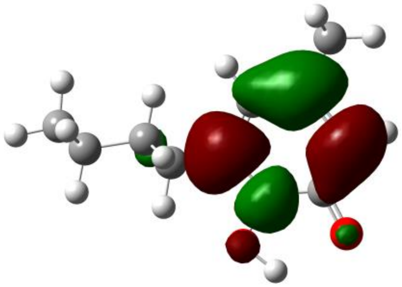	**- 8.864879**	**- 0.518489**	**8.346389**	**4.69164**	**4.173194**

**Griseofulvin**						
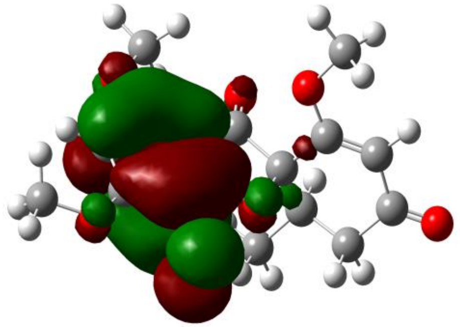	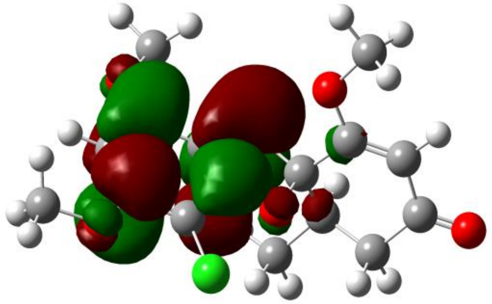	**- 9.227333**	**- 0.832985**	**8.394347**	**5.03015**	**3.780681**
**Fosinopril**						
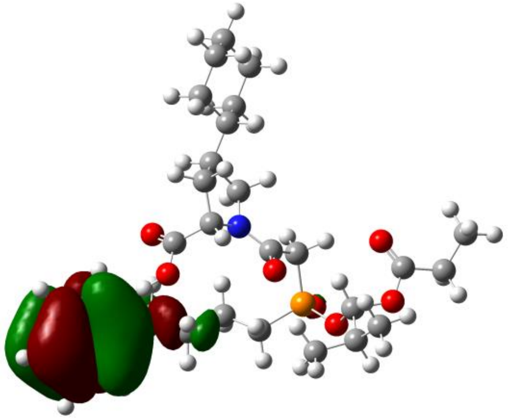	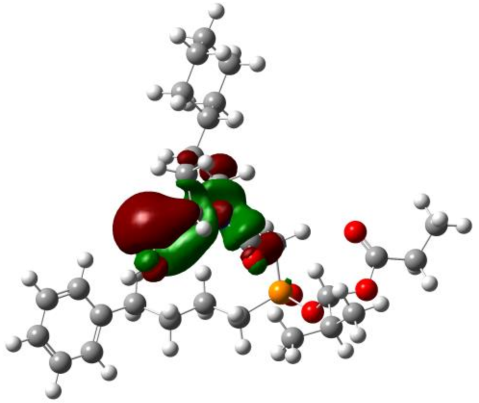	**- 9.52807**	**- 0.088852**	**9.439217**	**4.80846**	**4.719608**
**Furosemide**						
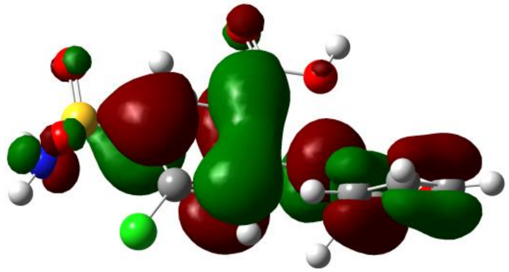	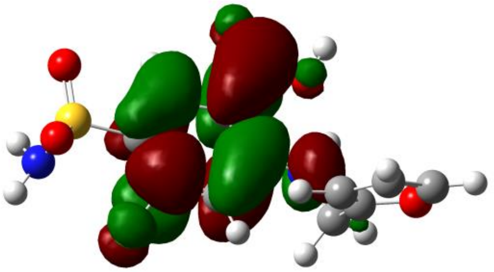	**- 9.450583**	**- 0.988114**	**8.462468**	**5.2193**	**4.231234**

The HOMO and LUMO energies are calculated in eV by DFT B3LYP.

Green and dark red isosurfaces of HOMO and LUMO indicate positive and negative values, respectively.

(1) $\Delta E = E_{LUMO} - E_{HOMO}$

(2) $\lambda = \dfrac{E_{LUMO} + E_{HOMO}}{2}$

(3) $\eta = \dfrac{E_{LUMO} - E_{HOMO}}{2}$

7.2 Mulliken population analysis for partial atomic charges for all ligands (*i.e.*, drugs) in this manuscript

**Meprobamate**	**Ciclopirox**	**Clotrimazole**
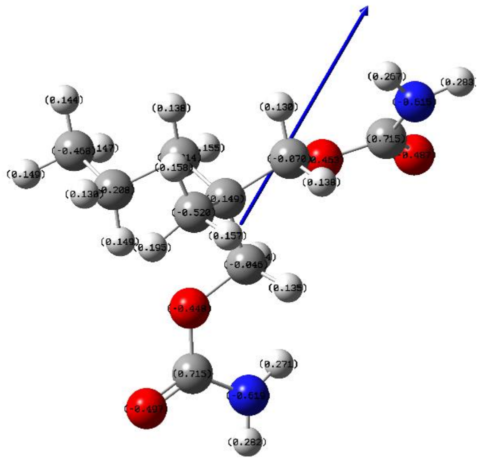	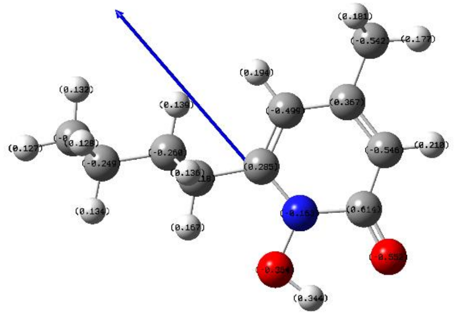	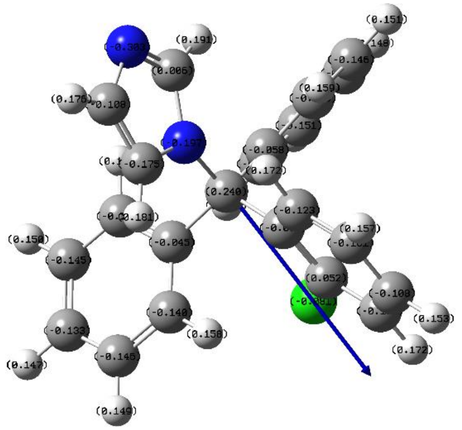

**Griseofulvine**	**Naftifine**	**Natamycin**
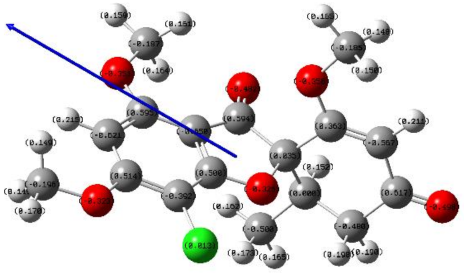	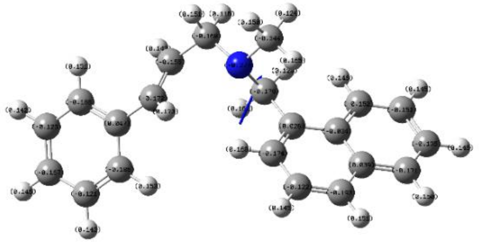	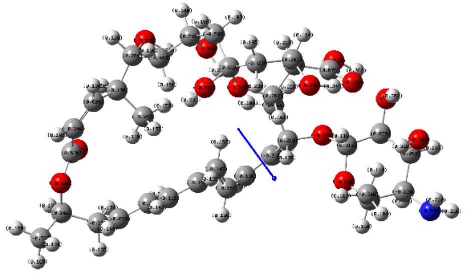
**Nystatin**	**Oxiconazole**	**Tolnaftate**
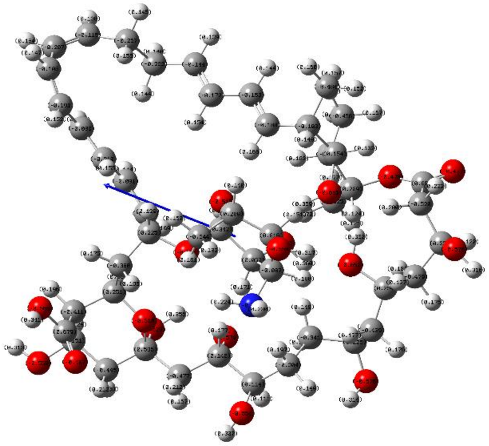	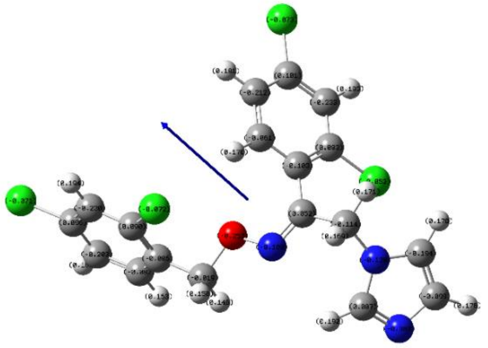	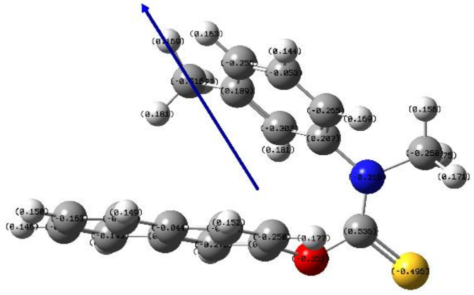

Azelaic acid	Abiraterone	Progesterone
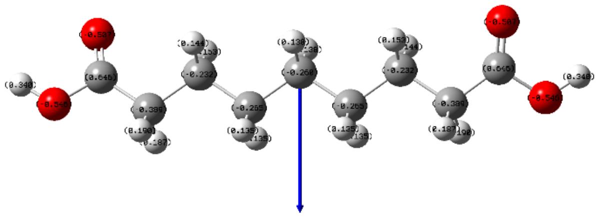	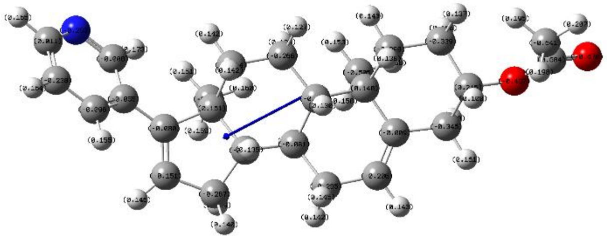	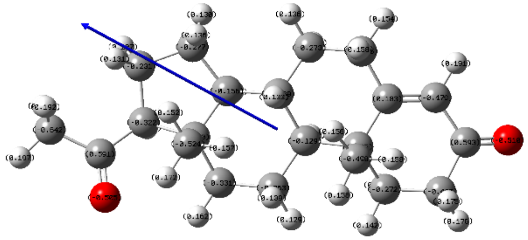
**Fosinopril**	**Furosemide**	
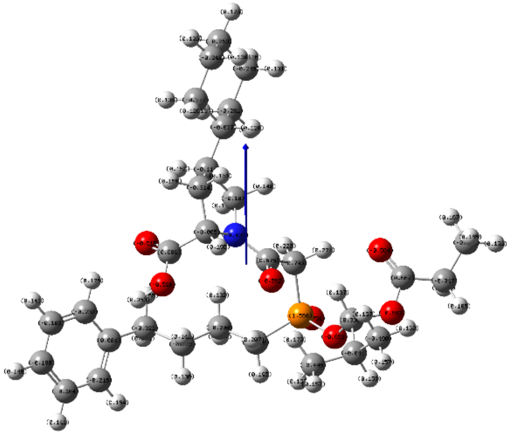	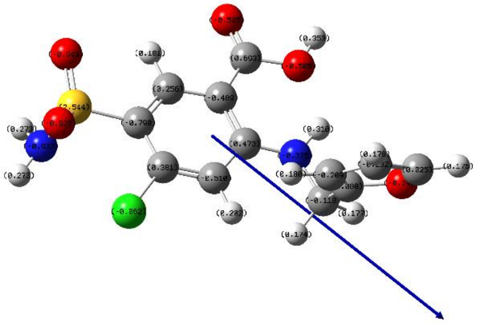	

7.3. Molecular electrostatic potential surfaces for all ligands (*i.e.*, drugs) in this manuscript

Meprobamate	Ciclopirox	Clotrimazole
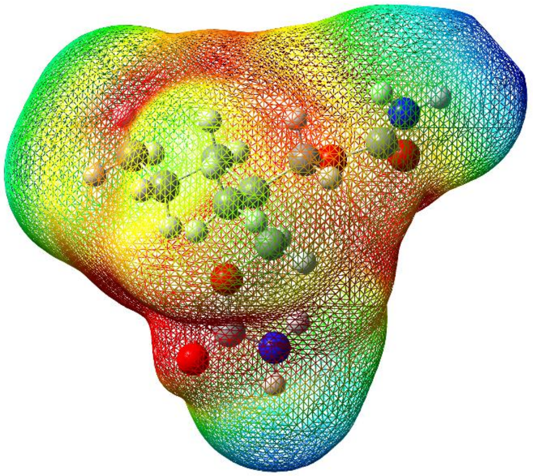	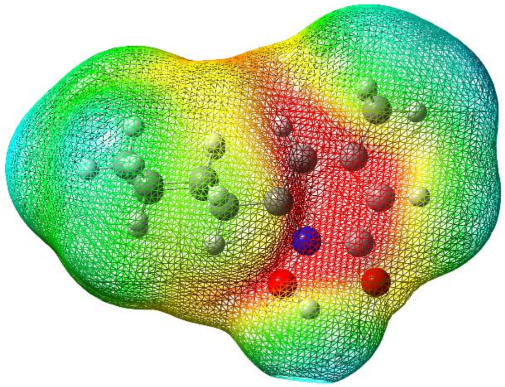	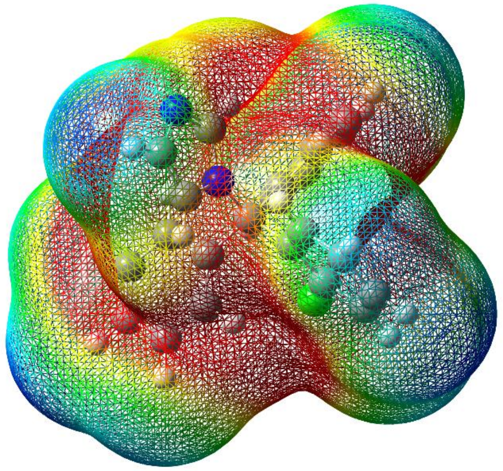
**Griseofulvine**	**Naftifine**	**Natamycin**
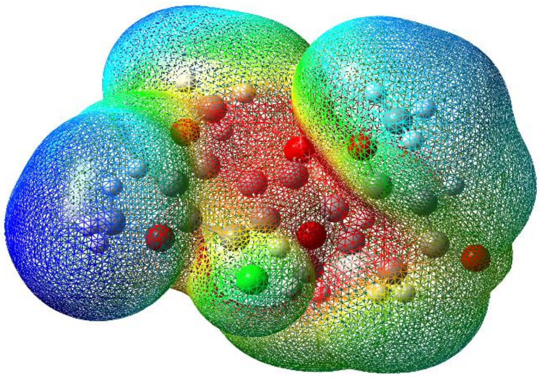	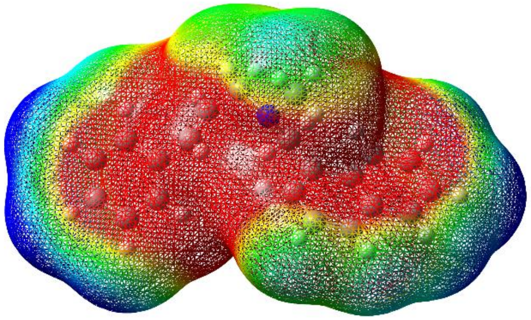	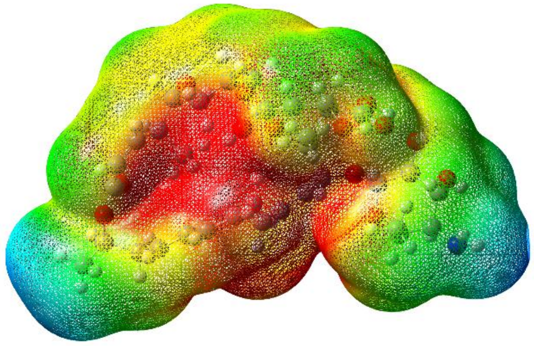

Nystatin	Oxiconazole	Tolnaftate
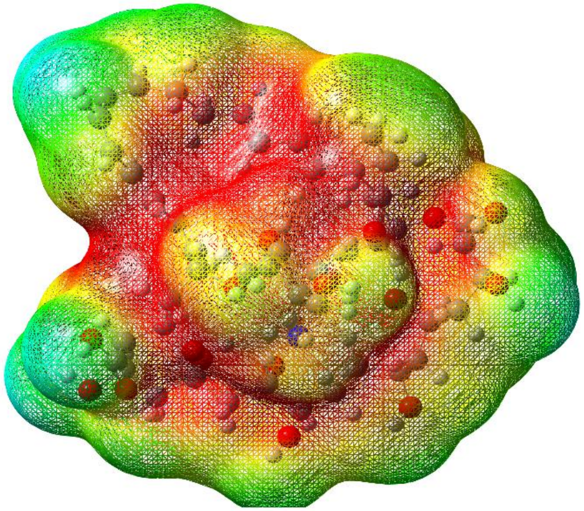	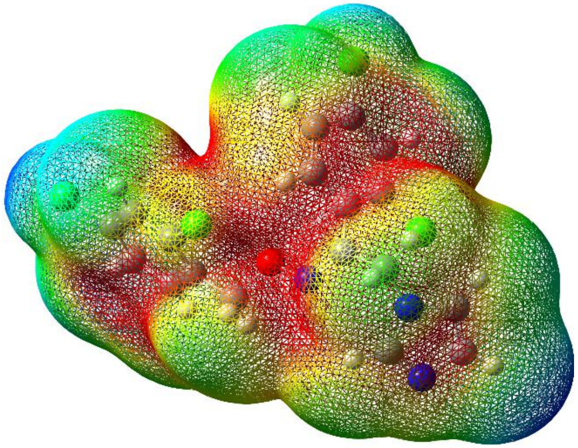	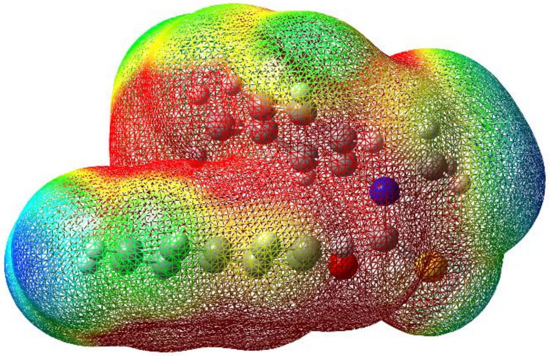
**Azelaic acid**	**Abiraterone**	**Progesterone**
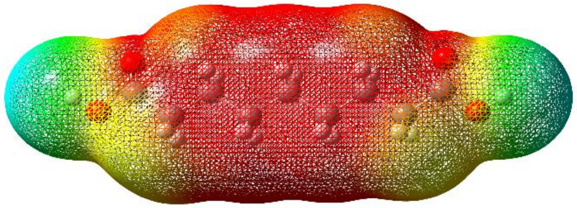	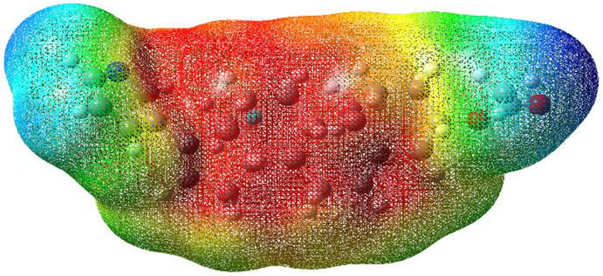	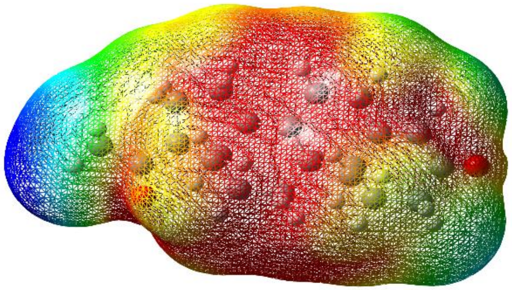

Fosinopril	Furosemide
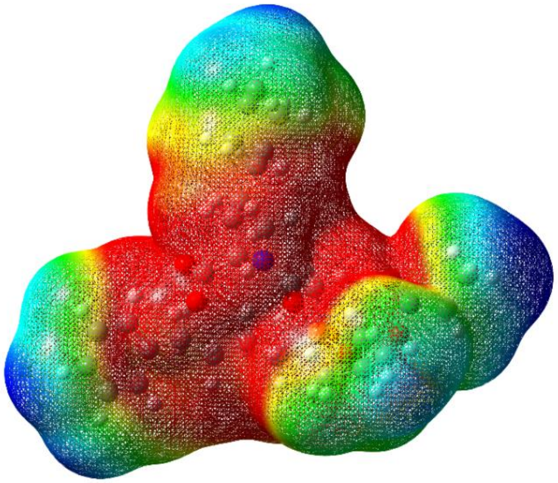	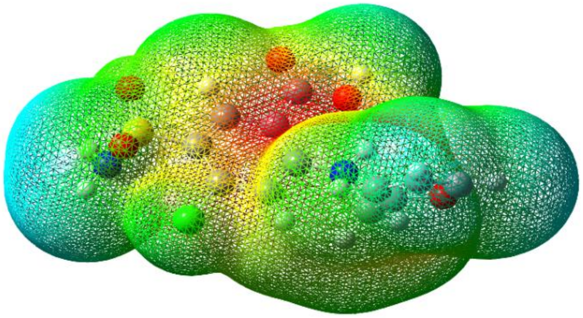

8. Docking results validation

We evaluate the performance of Autodock 4.2.6 by redocking and then expressing the results as root-mean-square deviation (RMSD) in Å. As presented by R.R. Nunes *et al*. (Nunes, R. R.; *et al*. Mem. Inst. Oswaldo Cruz 2019, 114(0), 1–10), the predictions of RMSD ≤ 2 Å indicate a successful docking. We performed all the calculations in duplicate and expressed the results as average. The redocking involved the overlapping of the ligands for calculating the RMSD with the Discovery Studio software.

Table S14. RMSD values (in Å) for **Azelaic acid** blind redocking, using the **Autodock 4.2.6** software.

**Target**	**Ligand**			
	Azelaic acid	Progesterone	Fosinopril	Furosemide
Estrogen receptor alpha	0.352	0.132	0.124	0.244
Estrogen receptor beta	0.355	0.212	0.255	0.262
Progesterone receptor	0.379	0.255	0.185	0.259
Steroid 17-alpha-hydroxylase/17,20 lyase	0.312	0.312	0.229	0.327
Androgen receptor	0.344	0.242	0.228	0.249
Mineralocorticoid receptor	0.227	0.346	0.309	0.381

Table S15. The representation of **Autodock 4.2.6** molecular redocking. The best docking pose first obtained for **Azelaic acid** with Autodock 4.2.6 is in red, and the redocked ligand is in black.

**Target**	**Azelaic acid**	**Target**	**Azelaic acid**
Estrogen receptor alpha	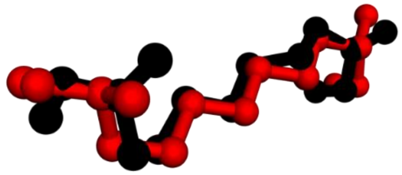	Steroid 17-alpha-hydroxylase/17,20 lyase	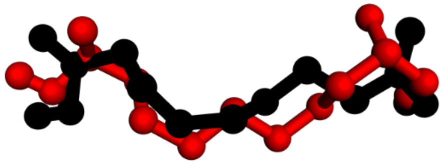
Estrogen receptor beta	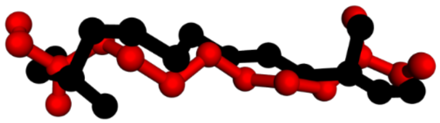	Androgen receptor	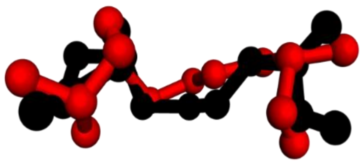
Progesterone receptor	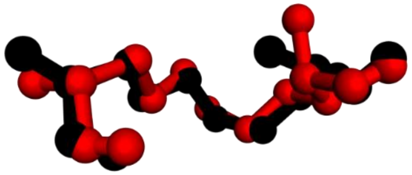	Mineralocorticoid receptor	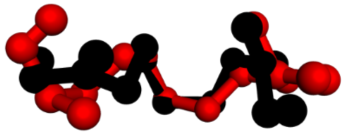

Table S16. RMSD values (in Å) for **Meprobamate** blind redocking, using the **Autodock 4.2.6** software.

**Target****	**Ligand***										
	L1	L2	L3	L4	L5	L6	L7	L8	L9	L10	L11
T1	0.309	0.453	nt	nt	nt	nt	nt	nt	nt	0.313	0.493
T2	0.3	nt	0.344	nt	nt	nt	nt	nt	nt	0.333	0.385
T3	0.350	0.473	nt	nt	nt	nt	nt	nt	nt	0.466	0.945
T4	0.383	nt	nt	0.376	0.347	nt	nt	nt	nt	0.365	0.599
T5	0.422	0.352	nt	nt	nt	0.355	0.483	nt	nt	0.432	0.435
T6	0.495	nt	nt	nt	nt	nt	nt	0.244	nt	0.543	0.384
T7	0.319	nt	nt	nt	nt	nt	nt	nt	0.247	0.245	0.434

Ligands*: L1 – Meprobamate, L2 – Clotrimazole, L3 – Oxiconazole, L4 – Naftifine, L5 – Tolnaftate, L6 – Nystatin, L7 – Natamycine, L8 – Ciclopirox, L9 – Griseofulvin, L10 – Fosinopril, L11 - Furosemide

Targets**: T1 – Lanosterol 14-alpha demethylase, T2 – Lanosterol synthase, T3 – Intermediate conductance calcium-activated potassium channel protein 4, T4 – Squalene monooxygenase, T5 – Squalene monooxygenase, T6 – Sodium/potassium-transporting ATPase subunit alpha, T7 – Sodium/potassium-transporting ATPase subunit alpha.

nt – not tested

Table S17. The representation of **Autodock 4.2.6** molecular redocking: the best docking pose first obtained for **Meprobamate** with Autodock 4.2.6 is in red, and the redocked ligand is in black.

Target	Meprobamate	Target	Meprobamate
Lanosterol 14-alpha demethylase	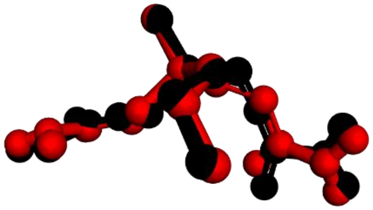	Squalene monooxygenase	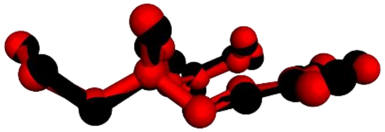
Lanosterol synthase	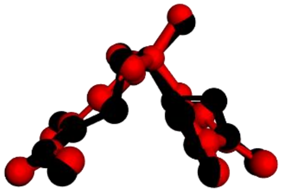	Ergosterol	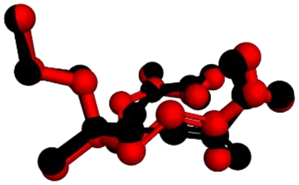
Intermediate conductance calcium-activated potassium channel protein 4	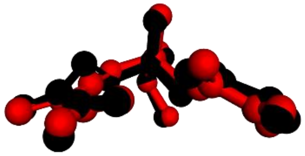	Sodium/potassium-transporting ATPase subunit alpha	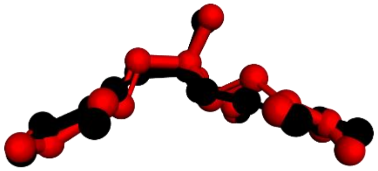
Tubulin	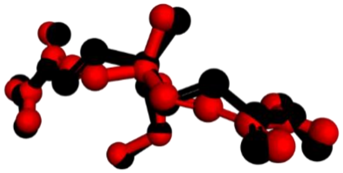		

Second, we performed a molecular docking comparative analysis between **Autodock 4.2.6** and **AutoDock Vina** to assess the docking method's repeatability and reproducibility. AutoDock Vina predicts a similar binding interaction between ligands-targets compared to the Autodock 4.2.6 software (in terms of RMDS mean values).

Table S18. The average RMSD values (in Å) for **Azelaic acid** blind redocking using the **AutoDock Vina** software.

**Target**	**Ligand**			
	Azelaic acid	Progesterone	Fosinopril	Furosemide
Estrogen receptor alpha	**0.299**	0.466	0.503	0.459
Estrogen receptor beta	**0.904**	0.534	0.593	0.546
Progesterone receptor	**0.561**	0.449	0.473	0.465
Steroid 17-alpha-hydroxylase/17,20 yase	**1.016**	0.359	0.584	0.685
Androgen receptor	**0.662**	0.456	0.443	0.467
Mineralocorticoid receptor	**0.914**	0.441	0.440	0.566

Table S19. The average RMSD values (in Å) for **Meprobamate** blind redocking using the **AutoDock Vina** software.

**Target****	**Ligand***										
	L1	L2	L3	L4	L5	L6	L7	L8	L9	L10	L11
T1	**0.387**	0.566	nt	nt	nt	nt	nt	nt	nt	0.303	0.459
T2	**0.313**	nt	0.328	nt	nt	nt	nt	nt	nt	0.393	0.346
T3	**0.373**	0.349	nt	nt	nt	nt	nt	nt	nt	0.473	0.465
T4	**0.338**	nt	nt	0.382	0.384	nt	nt	nt	nt	0.414	0.385
T5	**0.330**	0.356	nt	nt	nt	0.342	0.485	nt	nt	0.243	0.267
T6	**0.410**	nt	nt	nt	nt	nt	nt	0.236	nt	0.340	0.366
T7	**0.401**	nt	nt	nt	nt	nt	nt	nt	0.295	0.311	0.458

Ligands*: L1 – Meprobamate, L2 – Clotrimazole, L3 – Oxiconazole, L4 – Naftifine, L5 – Tolnaftate, L6 – Nystatin, L7 – Natamycine, L8 – Ciclopirox, L9 – Griseofulvin, L10 – Fosinopril, L11 - Furosemide

Targets**: T1 – Lanosterol 14-alpha demethylase, T2 – Lanosterol synthase, T3 – Intermediate conductance calcium-activated potassium channel protein 4, T4 – Squalene monooxygenase, T5 – Squalene monooxygenase, T6 – Sodium/potassium-transporting ATPase subunit alpha, T7 – Sodium/potassium-transporting ATPase subunit alpha.

nt – not tested
